# *Ph2* encodes the mismatch repair protein MSH7-3D that inhibits wheat homoeologous recombination

**DOI:** 10.1038/s41467-021-21127-1

**Published:** 2021-02-05

**Authors:** Heïdi Serra, Radim Svačina, Ute Baumann, Ryan Whitford, Tim Sutton, Jan Bartoš, Pierre Sourdille

**Affiliations:** 1 Genetics, Diversity and Ecophysiology of Cereals, UMR 1095, INRAE, Université Clermont Auvergne, Clermont-Ferrand, France; 2 Institute of Experimental Botany of the Czech Academy of Sciences, Centre of the Region Hana for Biotechnological and Agricultural Research, Olomouc, Czech Republic; 3 School of Agriculture, Food and Wine, University of Adelaide, Waite Campus, PMB1, Glen Osmond, SA Australia; 4 South Australian Research and Development Institute, Adelaide, SA Australia; 5 Present Address: Genetics, Reproduction and Development, CNRS, Inserm, Université Clermont Auvergne, Clermont-Ferrand, France

**Keywords:** Plant breeding, DNA recombination, Polyploidy in plants

## Abstract

Meiotic recombination is a critical process for plant breeding, as it creates novel allele combinations that can be exploited for crop improvement. In wheat, a complex allohexaploid that has a diploid-like behaviour, meiotic recombination between homoeologous or alien chromosomes is suppressed through the action of several loci. Here, we report positional cloning of *Pairing homoeologous* 2 *(Ph2)* and functional validation of the wheat DNA mismatch repair protein MSH7-3D as a key inhibitor of homoeologous recombination, thus solving a half-century-old question. Similar to *ph2* mutant phenotype, we show that mutating MSH7-3D induces a substantial increase in homoeologous recombination (up to 5.5 fold) in wheat-wild relative hybrids, which is also associated with a reduction in homologous recombination. These data reveal a role for MSH7-3D in meiotic stabilisation of allopolyploidy and provides an opportunity to improve wheat’s genetic diversity through alien gene introgression, a major bottleneck facing crop improvement.

## Abstract (web summary)

In the allohexaploid genome of wheat, meiotic recombination between homoeologues is suppressed through the action of several loci. Here, the authors report the cloning of the long sought-after gene *Ph2* and show its function in reduction of homoeologous recombination.

## Introduction

Crop wild relatives provide a valuable source of genes and allelic variants for abiotic stress tolerance, disease resistance and quality traits that are important for breeding, particularly in the context of human population growth and a changing climate. Remarkable progress has been made over the last 80 years, with notable boosts in the 1970s and 1980s^1^, in knowledge and resulting methodology to allow utilisation of wild relatives in wheat breeding. However, an important challenge still facing breeders now is the ability to routinely perform DNA-introgression, a process by which distantly related chromosomes exchange genetic information that is passed onto progeny. The transfer of chromatin between pairing maternal and paternal chromosomes relies on recombination, a process which occurs in all sexually reproducing species during meiosis^2^.

The genetics of chromosome pairing and meiotic recombination is complicated by the allopolyploid nature of many crops, a widespread feature in the plant kingdom^3^. For example, hexaploid bread wheat (*Triticum aestivum* L., AABBDD 2*n* = 6*x* = 42), which derives from two successive interspecific crosses involving three diploids^4,5^, has three sets of related homoeologous chromosomes. Genetic and cytogenetic studies have revealed the presence of several pairing homoeologous (*Ph*) loci that ensure wheat behaves as a diploid during meiosis, with only homologous chromosomes of the same sub-genome (AA, BB or DD) pairing and recombining. The two main loci controlling homoeologous recombination are located on chromosome-arms 5BL and 3DS, named *Ph1* and *Ph2*, respectively^6–9^.

Analysis of *Ph1* gene mutants in tetraploid *(ph1c)*^10,11^ and hexaploid *(ph1b)*^12^ first identified interstitial deletions involving an ~0.84-µm region and a 1.05-µm region around the gene, respectively^13,14^. Subsequent physical mapping localised *Ph1* to a 2.5 Mbp region on chromosome 5BL^15^. This region contains a meiotic gene *ZIP4* and a heterochromatin tandem repeat block, inserted within a cluster of *CDK2-like* genes^15–18^. ZIP4 is a ZMM protein involved in homologous recombination and may act as a hub through physical interactions with components of the chromosome axis and other ZMMs^19^. Recent evidence now points to *TaZIP4-B2* (the additional ZIP4 copy on 5BL) as being responsible for the effect of this locus on homoeologous recombination^20,21^. Although the exact mode of action is unknown, *TaZIP4-B2* seems to act as a focal point, facilitating physical interactions between components of the chromosome axis and crossover machinery^17^.

In comparison to *Ph1*, the causative gene sequence for *Ph2* is yet to be determined. Analysis of the irradiation-mutant *ph2a* in comparison to the syntenic region on rice chromosome 1 estimated the deletion to be at least 80 Mb in size^22^, but more likely to span a 120 to 125 Mb region^23^ on the terminal portion of 3DS. Research aiming to identify *Ph2* has resulted in the isolation of a number of candidate meiotic genes from this region on 3DS. These include the genes *WM1*^24,25^, *WM3*^26^, *WM5*^27^ and *TaMSH7*^28,29^. Despite these attempts, the region and its candidate meiotic gene content was deemed too large and complex to confidently identify the *Ph2* causative sequence using the *ph2a* deletion mutant alone. The chemically induced *ph2b* mutant^30^, thought to contain either a point mutation or small lesion at *Ph2*, offered the prospect of identifying the causative gene sequence.

Identifying the genetic control and underlying mechanism of action of *Ph2* would provide valuable knowledge, and enable novel resources to be developed for introgressing alien sequences from related species into bread wheat. The use of *ph2* mutation could be of particular interest to breeders and geneticists as it induces only a minimal disruption to endogenous homologous recombination^12,31,32^ but reinforces *ph1b*’s effect of promoting homoeologous recombination in some crosses^33^.

Here, we report the positional cloning of *Ph2* from a 121.16 Mb candidate region on 3DS. Based on the analysis of a set of specifically created 3DS deletion mutants^23^ combined with exome sequencing and transcriptome analysis of *ph2a* and *ph2b* mutants versus wild-type, we identify *TaMSH7-3D*, a gene encoding a plant specific DNA mismatch repair protein. Using independent ethyl methanesulfonate (EMS) generated *msh7-3D* mutants crossed with wheat wild relative *Aegilops variabilis*, we demonstrate that *msh7-3D* mutants recapitulate the *ph2* phenotype with a highly significant (5.5-fold) increase in homoeologous recombination and a reduction in homologous recombination. These data suggest that, in addition to *TaZIP4-B2*, *TaMSH7-3D* is an attractive target for facilitating alien gene introgression in pre-breeding and breeding programs.

## Results

### Molecular characterisation of *ph2a* and *ph2b* mutations

The *Ph2* pairing homoeologous locus is located on chromosome-arm 3DS within the terminally deleted region of irradiation-mutant *ph2a*^9,31^. Marker-based analysis of *ph2a* recently revealed that minimum deletion size is 120 Mb with a maximum of 125 Mb^23^. To precisely delineate the deletion breakpoint, *ph2a* was genotyped using a high-density SNP genotyping array (35 K SNP Affymetrix Axiom^*®*^). Genotyping data showed the deletion breakpoint on chromosome 3D located between markers AX-178057815 and AX-178057206 at the coordinates 120.722.379 and 121.539.725, respectively (Supplementary Fig. 1a). This was further refined using exome capture of *ph2a*: the deletion breakpoint is around the coordinate 121.163.000, upstream of the gene *Traes3D01G153800* (Supplementary Fig. 1b). Using the newly available Chinese Spring Reference Genome v1.0^34^, we identified 1577 genes within the deleted *ph2a* region.

To identify possible candidate genes for *Ph2*, we performed an exome capture of the EMS induced *ph2b* mutant, in which a point mutation was proposed as being responsible for the observed phenotype^30^. Comparison between *ph2b* exome sequence and the Chinese Spring reference genome highlighted 165 single nucleotide differences within the 121.16 Mb deleted *ph2a* region. These consisted mainly of G to A and C to T transitions, as would be expected from alkylation by EMS treatment. We detected 59 SNPs within genic regions (including 5′ and 3′ UTR and potential promoter regions), among these 36 were exonic mutations (13 synonymous, 21 non-synonymous and 2 non-sense mutations) and one likely to affect transcript splicing (Supplementary Table 1). Considering only those genes that contain exonic SNPs predicted to result in either non-synonymous amino acid changes, protein truncations (premature STOP codons) or alternate splicing, the total number of *Ph2* candidate genes was reduced to 24.

### *Ph2* locates within a 14.3-Mb region on 3DS

**Fig. 1:**
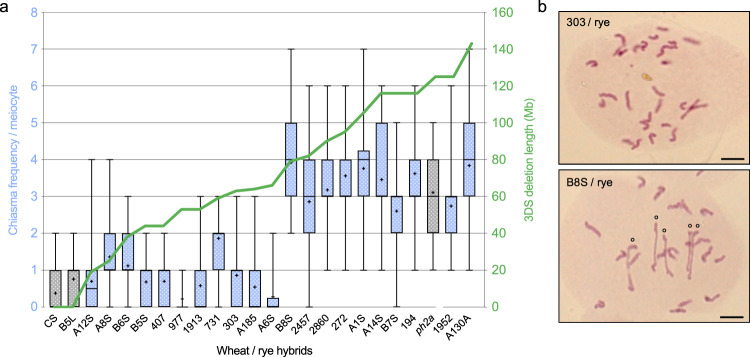
Physical mapping of *Ph2* using wheat/rye hybrids carrying 3DS terminal deletions. Meiotic phenotypes at metaphase I of 24 wheat/rye hybrids were analysed cytogenetically. This analysis includes three wheat/rye hybrid controls (in grey) derived from: wild-type wheat cv. Chinese Spring (CS), CS carrying a 357 Mb deletion of 3DL but no 3DS deletion (B5L) and CS *ph2a* mutant (*ph2a*). **a** Box plots showing minimum, first quantile, median (horizontal middle line), third quantile and maximum count of chiasma frequency per meiocyte for each hybrid. 50 meiocytes have been examined by hybrid and the mean values are represented by crosses. The green line indicates length (in Mb) of the 3DS terminal deletion carried by each hybrid. **b** Representative meiocytes at metaphase I of ‘303/rye’ and ‘B8S/rye’ haploid hybrids showing chromosome configurations. ‘303/rye’ meiocyte exhibits 28 univalents while ‘B8S/rye’ shows 18 univalents and 5 rod bivalents (o). Scale bars represent 10 μm for both panels. Source data underlying Fig. 1a are provided as a Source Data file.

Since these 24 candidate genes were dispersed over the entire length of the chromosomal region deleted in *ph2a*, we then sought to delineate *Ph2* spatially. With this purpose, we developed a series of 113 wheat deletion lines carrying terminal deletions of chromosome 3D^23^. Among these lines, a subset of 32 that possessed 3DS deletions ranging in size from 6.5 to 142.6 Mb were selected. The region between each adjacent deletion breakpoint of the tiled series did not exceed 14.3 Mb. Since the mutant phenotype for *Ph2* is easily discernible in ABDR haploid hybrids^35^, each selected deletion line (in 3D monosomic constitution) was crossed with rye (RR, 2*n* = 14) and hybrids carrying a mutant 3D chromosome (terminal deletion) were selected in the progeny using 3D specific markers. We screened for the presence of *Ph2* by characterising meiotic behaviour at metaphase I of 22 ABDR hybrids (each carrying a 3D chromosome with a terminal deletion). While haploid sets of wheat and rye homoeologous chromosomes rarely associated in wild-type hybrids (0.38 ± 0.10 chiasma/meiocyte), mean chiasma frequency was significantly increased in the *ph2a* mutant context (3.10 ± 0.13 chiasmata/meiocyte), indicating that formation of chiasmata between non homologous chromosomes occurs more frequently in the absence of *Ph2* (Fig. 1; Supplementary Table 2)^31^. Cytogenetic analyses of the generated wheat 3D-deletion line/rye hybrids revealed that individuals carrying a 3DS terminal deletion of 79.2 Mb or more exhibit a high chiasma frequency (ranging from 2.60 to 3.92 chiasmata/meiocyte), similar to that observed for *ph2a* (Fig. 1; Supplementary Table 2). However, hybrids carrying a 3DS terminal deletion shorter than 64.9 Mb (minimal breakage position of the A6S line) showed less than 2 chiasmata/meiocyte indicating the presence of *Ph2* and its ability to inhibit homoeologous recombination within these lines. Taken together, these data clearly demonstrate that *Ph2* is located within a 14.3 Mb genetic interval ranging from 64.9 to 79.2 Mb on chromosome-arm 3DS.

### *TaMSH7-3D* is a unique candidate for *Ph2*

**Fig. 2:**
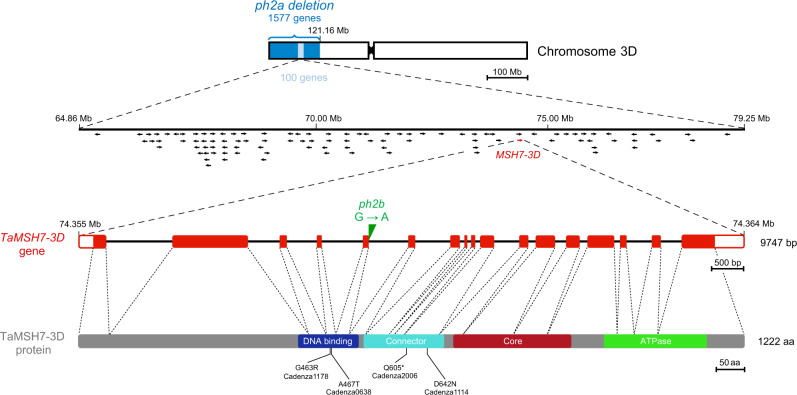
Positional cloning of *Ph2* identified *TaMSH7-3D* as the causative agent. Schematic representation of chromosome 3D showing *ph2a* deletion (dark blue). Further deletion-line mapping localised *Ph2* to a 14.3 Mb genetic interval (light blue) containing 100 genes (represented by arrows). Among them, the only gene identified to contain an exonic SNP predicted to either result in a non-synonymous amino acid (aa) change, protein truncation or alternate splicing in *ph2b* is *TaMSH7-3D* (TraesCS3D02G119400) at the coordinates 74.355.077 – 74.364.823 (highlighted in red). The G to A transition at position 74.359.312 in *ph2b* sequence is shown (in green) in the gene structural schematic for *TaMSH7-3D*. *TaMSH7-3D* contains 17 exons (red rectangles) and 16 introns (black lines) with a total length of 9747 bp. 5′ and 3′ UTR’s are represented by white rectangles. The schematic representation of the TaMSH7-3D protein is shown below. Regions encoding predicted protein domains are highlighted by coloured rectangles: N-terminal mismatch-recognition domain (aa 405-515), connector domain (aa 525-672), core domain (aa 689-905) and C-terminal ATPase domain (aa 967-1154) containing a Walker A motif. Thin vertical lines below indicate positions of the aa changes in the four TILLING *Tamsh7-3D* mutants used in this study and * represents stop mutation.

Among the 24 candidate genes identified by *ph2b* exome sequencing, only one located between positions 64.9 and 79.2 Mb on 3DS. This gene, *TraesCS3D02G119400*, contains 17 exons and 16 introns with a total length of 9747 bp and encodes the DNA mismatch repair protein TaMSH7-3D (Fig. 2). In *ph2b*, a G to A transition was detected at position 74.359.312 and confirmed by Sanger sequencing. It affects the first nucleotide of the splicing pattern GTAAGT at the junction between exon 5 and intron 5 and is predicted to compromise correct splicing of the transcript. No other unique mutation from *ph2b*-derived sequences was detected for this gene’s A or B homoeologues (*TraesCS3A02G117500* and *TraesCS3B02G136600*, respectively) nor for previously identified potential candidates for *Ph2*: the *WM1* gene family (*TraesCS3D02G034300,* *TraesCS3D02G034500*, *TraesCS3D02G034700*, *TraesCS3D02G034900*, *TraesCS3D02G035200*, *TraesCS3D02G035100*)^24,25^, *WM3* (*TraesCS3D02G152900*)^26^ or *WM5* (*TraesCS3D02G140300*)^27^. We were unable to confirm the presence of three previously identified SNPs in the *TaMSH7-3D* coding sequence of *ph2b*, none of which were deemed to result in a non-functional or malfunctioning protein^29^. To determine whether the G to A SNP we identified here affects intron-5 splicing (leading to a predicted loss of protein function and therefore the *ph2* phenotype), we performed a high-depth RNAseq from wild-type and *ph2b* mutant anthers staged from pre-meiotic interphase to metaphase I. RNAseq data from *ph2b* confirmed the presence of the splice junction mutation and showed that this mutation leads to the use of a downstream splice site. This results in a frame-shift and thereby creates a premature in-frame STOP codon (Supplementary Fig. 2). This STOP codon is predicted to result in a truncated, non-functional TaMSH7-3D protein missing major functional domains, specifically the core domain and the C-terminal ATPase domain (Fig. 2). Taken together, deletion-line mapping combined with exome and transcriptome sequencing of the *ph2b* mutant identifies *TaMSH7-3D* as a unique candidate for *Ph2*.

### Validation of *TaMSH7-3D* as the causative gene for *Ph2*

**Fig. 3:**
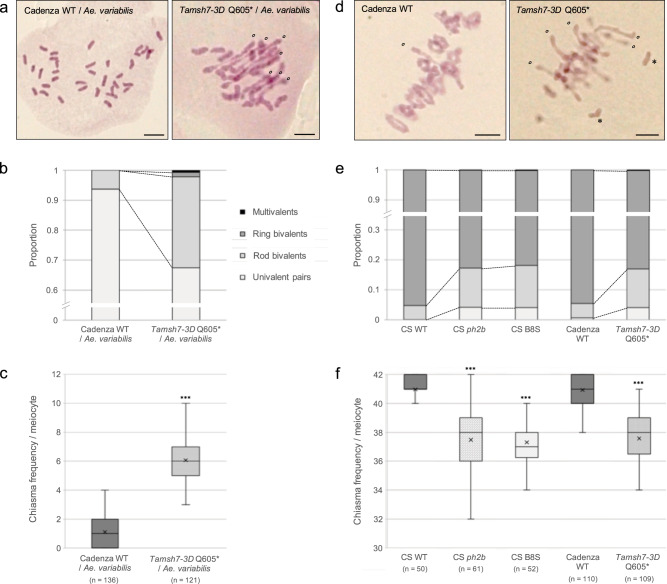
Loss-of-function *TaMSH7-3D* mutations promote homoeologous recombination and reduce homologous recombination. **a**, **b**, **c** Meiotic chromosome phenotype at metaphase I of wheat cv. Cadenza/*Aegilops variabilis* haploid hybrids. Hybrids have 35 homoeologous chromosomes and the presence of bivalents and/or multivalents at metaphase I are therefore markers of homoeologous recombination (i.e. crossovers established between homoeologous chromosomes). **d**, **e**, **f** Meiotic chromosome phenotype at metaphase I of wheat cv. Chinese Spring (CS) or Cadenza. In a wild-type (WT) context, most homologous chromosome pairs are connected by two distal crossovers that form ring bivalents at metaphase I. The presence of rod bivalents and/or univalents reveals a reduction in homologous recombination efficiency. **a**, **d** Chromosome configurations of representative meiocytes at metaphase I. Open circles and asterisks indicate rod bivalents and univalents, respectively. Scale bars represent 10 μm for all panels. **b**, **e** Stacked bar graphs showing mean proportions of each metaphase I chromosome configuration (univalent pairs, rod and ring bivalents, multivalents). **c**, **f** Box plots showing minimum, first quantile, median (horizontal middle line), third quantile and maximum count of chiasma frequency per meiocyte. Mean values are represented by crosses. *n* number of cells examined. To test for differences between each mutant and corresponding wild-type control, two-sided Mann–Whitney tests adjusted for multiple comparisons were performed. *** reports a *p* value <0.0001 (see Supplementary Tables 4 and 5 for exact *p* values). Source data underlying Fig. 3c and 3f are provided as a Source Data file.

To functionally validate that *TaMSH7-3D* affects homoeologous recombination, we took advantage of the Targeting Induced Local Lesions In Genome (TILLING) population of 1200 wheat mutant lines of the variety Cadenza and the corresponding databases cataloguing mutations identified through exome sequencing (www.wheat-tilling.com)^36,37^. Screening by BLAST search identified 127 possible mutants for *TaMSH7-3D* (*Traes_3DS_72259A292.1*) within the population. We selected four mutant lines with either a high probability of being knocked-out (*Tamsh7-3D* Q605*; stop codon gained; data described hereafter) or carrying missense mutations (Supplementary Table 3, Supplementary Figs. 3 and 4; data described in supplementary information). Considering the wheat variety Cadenza does not produce viable F1’s when crossed with rye, the selected *TaMSH7-3D* mutants, as well as a wild-type Cadenza (Cad wt), were crossed with the wheat wild relative *Aegilops variabilis* (UUSS, 2*n* = 4*x* = 28). The frequencies of univalents, rod and ring bivalents as well as multivalents were scored at meiotic metaphase I in the resulting F1 wheat/*Ae. variabilis* hybrids and were used to calculate total chiasma frequency per cell. Cad wt/*Ae. variabilis* hybrids exhibited on average 32.79 (± 0.18) univalents and 1.10 (± 0.09) rod bivalents corresponding to a mean chiasma frequency of 1.10 (± 0.09) per meiocyte at metaphase I (Supplementary Table 4). In contrast, *Tamsh7-3D* Q605*/*Ae. variabilis* hybrids exhibited a 5-fold increase in bivalent number (5.29 ± 0.15 rod and 0.26 ± 0.04 ring bivalents per meiocyte on average) and the presence of multivalents (0.12 ± 0.03) (Fig. 3a, b; Supplementary Table 4). This is associated with a highly significant 5.52-fold increase in mean chiasma frequency (6.07 ± 0.17; Mann–Whitney test, *p* = 7.43 × 10^−42^) (Fig. 3c; Supplementary Table 4). The absence of a full-length TaMSH7-3D protein thus induces a substantial increase in genome-wide homoeologous recombination within the hybrid context. This data clearly demonstrates that TaMSH7-3D inhibits recombination between homoeologous chromosomes.

Moreover, a previous study reported a slight reduction in homologous recombination efficiency in wheat in the absence of *Ph2*^32^. We first confirmed that chiasma frequency is significantly reduced in *ph2b* relative to Chinese Spring wild-type (37.48 ± 0.26 versus 40.98 ± 0.14, respectively; Mann–Whitney test, *p* = 3.04 × 10^−15^) due to an observed increase in univalent and rod bivalent frequencies for *ph2b* (Fig. 3e, f; Supplementary Table 5). As expected, *Tamsh7-3D* Q605* exhibited a similar phenotype (mean chiasma frequency of 37.58 ± 0.19) (Fig. 3d–f; Supplementary Table 5). Interestingly, rare trivalents and quadrivalents were also observed in *ph2b* and *Tamsh7-3D* Q605* meiocytes (but not in those of a wild-type background), revealing that homoeologous recombination occurs in wheat in the absence of *Ph2/TaMSH7-3D*, albeit in the presence of *Ph1*. To test for TaMSH7-3D dosage sensitivity, we quantified chiasma frequency when only one functional copy of *TaMSH7-3D* is present (i.e. *Tamsh-3D* Q605* heterozygote) relative to two. Heterozygous plants showed an intermediate phenotype between wild-type and *Tamsh-3D* Q605* homozygotes (Supplementary Table 5, Supplementary Fig. 5), indicative of a dosage dependent effect of TaMSH7-3D on homologous recombination.

Comparison of all genes containing EMS-derived SNPs in *Tamsh7-3D* Q605* and in *ph2b* identified 16 genes carrying mutations in both lines (Supplementary Table 6). Among these 16 genes, only *MSH7-3D* is located on 3DS (where *Ph2* gene function was previously shown to be present based on *ph2a* analysis^31^) and none of the 15 other genes carry mutation in *ph2a*. These data eliminate the possibility of another gene contributing to the *ph2* phenotype.

Taken together, these data demonstrate that *TaMSH7-3D* loss-of-function mutants have the capacity to recapitulate the *ph2* phenotype with a substantial increase in homoeologous recombination in wheat/*Ae. variabilis* hybrids and a slight reduction of homologous recombination in wheat. These findings reveal a key role for *TaMSH7-3D* in inhibiting recombination between homoeologous chromosomes and consequently, in assuring accurate chromosome segregation during meiosis.

### *Tamsh7-3D* reduces pollen viability but does not affect plant fertility

To assess whether meiotic behaviour disorders caused by *Tamsh7-3D* were associated with changes in fertility, we performed Alexander staining of pollen and scored the proportion of viable versus non-viable grains. Compared to wild-type, *Tamsh7-3D* Q605* showed a slightly higher proportion of non-viable pollen (*p* = 6 × 10^−6^, pairwise *t*-test with correction for multiple testing) (Supplementary Fig. 6, Supplementary Table 7). We also measured seed-set and observed that seed number per spike in *Tamsh7-3D* Q605* was not significantly reduced compared to wild-type (*p* = 0.43, pairwise *t*-test with correction for multiple testing) (Supplementary Fig. 6, Supplementary Table 8). These data demonstrate that *TaMSH7-3D* loss-of-function does not significantly affect wheat fertility (as comparable seed sets are observed in the mutants) although this mutation does disturb proper homologous recombination (and induces homoeologous recombination events in some meiocytes). These results are in agreement with studies of tomato and Arabidopsis, in which reduced *MSH7* expression or MSH7 loss-of-function (respectively) do not affect seed number^38,39^.

### *TaMSH7-3D* is expressed in anthers during meiotic prophase I

**Fig. 4:**
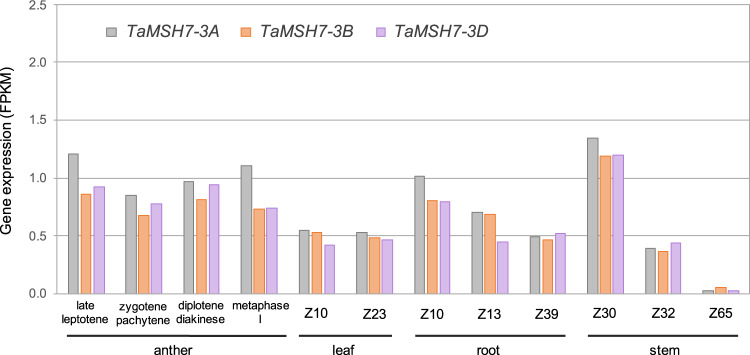
*TaMSH7-3A*, *TaMSH7-3B* and *TaMSH7-3D* genes are expressed in anthers at early meiosis and in somatic tissues. Relative expression (in FPKM: Fragments per kilo base of transcript per million mapped reads) of the three *TaMSH7* homoeologues in wild-type wheat anthers, leaves, roots and stems at various developmental stages. Stages according to Zadoks scale^69^: Z10, seedling; Z13: three leaves unfolded; Z23, main shoot and three tillers; Z30, pseudostem erection; Z32, two nodes; Z39, flag leaf ligule and collar visible, Z65, half of flowering complete. Gene expression in anthers was obtained following the method described by Lloyd et al.^59^ and data is available at http://wheat-urgi.versailles.inra.fr/Seq-Repository/Expression. Gene expression in leaves, roots and stems is from Pingault et al.^70^.

To precisely determine *TaMSH7-3D* expression over the course of early meiosis, transcript-profiling using a sub-staged meiotic time series was performed on whole-wheat anthers. Four meiotic stages were analysed: late leptotene, zygotene/pachytene, diplotene/diakinesis and metaphase I. RNA-seq data revealed that *TaMSH7-3D* (as well as *TaMSH7-3A* and *3B* homoeologues) is expressed for the entirety of prophase, which is in agreement with a role of *TaMSH7-3D* in control of homoeologous recombination at meiotic prophase I (Fig. 4). *TaMSH7-3D* expression however, is not restricted to meiosis considering similar transcript abundance was detected for each of the 3 A, 3B and 3D homoeologous copies in leaf, root and stem before flowering (Fig. 4)^40^. To investigate whether an absence of the pairing homoeologous gene *Ph1* could be compensated by an overexpression of *Ph2/TaMSH7-3D*, we compared *TaMSH7-3D* expression in wild-type versus the *ph1b* mutant background using RNA-seq data previously generated^41^. No significant change in *TaMSH7-3D* transcript abundance (or *TaMSH7-3A* and *3B)* was observed between wild-type and *ph1b* anthers at prophase I (Supplementary Fig. 7). This data indicates that the absence of *Ph1* does not feedback to cause significant changes in *TaMSH7* expression during meiosis in wheat.

### *TaMSH7-3D* is more highly conserved than 3A and 3B homoeologues both among wild and domesticated wheats

Comparison of *TaMSH7-3D* with its homoeologous copies revealed they share more than 97% sequence identity at the nucleotide sequence level, with *TaMSH7-3A* being equidistant to *TaMSH7-3B* and *TaMSH7-3D*. This is reflected in their deduced protein sequences, TaMSH7-3B and 3D are 96.3% identical to TaMSH7-3A and 97.2% identical to each other (Supplementary Table 9). About 25% of amino acid differences between the homoeologues are concentrated within the region from amino acid 760 to 880, thus in the MutS domain III corresponding to the core domain of the proteins (Supplementary Fig. 8).

Exome sequencing data revealed a high level of conservation of *TaMSH7-3D* among 436 bread wheat accessions studied. Only two haplotypes exist with the three identified SNPs exclusively localised within introns (Supplementary Table 10). In contrast, *TaMSH7-3A* and *3B* genes (inclusive of promoter regions) are more diverse with 12 and 30 polymorphisms identified within this population, respectively (Supplementary Table 10). By data mining NCBI’s small read archive, querying the 10+Genome Project data and DAWN^42^, we identified 14 accessions that contain a 28 bp deletion in *TaMSH7-3A*, likely leading to a non-functional truncated protein (Supplementary Fig. 9, Supplementary Table 11). Pedigree analysis allowed us to deduce the most likely ancestor as Red-Fife, from which the deletion was transmitted (Supplementary Fig. 10). Considering only polymorphisms located within exons, we calculated the number of variants found in the 436 wheat lines within each gene and reported it relative to exon length. 13.69, 6.74 and 0 variants per 10 kb of exons were found in *TaMSH7-3A, TaMSH7-3B* and *TaMSH7-3D*, respectively. Consistent with the low genetic diversity of the D-genome, *TaMSH7-3D* is very well conserved within this collection. The *TaMSH7-3A* and *TaMSH7-3B* copies are however highly polymorphic: *TaMSH7-3A* sits within the top 30% of most diverse A genome derived genes and *TaMSH7-3B* within the top 20% of most diverse B-genome derived genes. Taken together, these data demonstrate that *Ph2*/*TaMSH7-3D* is more highly conserved than its homoeologues among wild and domesticated wheats, consistent with a major role for this gene in homoeologous recombination inhibition.

MSH7 is also highly conserved more broadly amongst the grasses. Bread wheat MSH7 proteins indeed show more than 70% amino acid identity with MSH7 homologues of all studied *Poaceae* species (Supplementary Fig. 11, Supplementary Table 12). Protein sequence alignment of MSH7 homologues revealed that the main functional domains of the protein (MutS domain I, II, III and V) are particularly well conserved although the remaining regions of the protein display lower level of amino acid identity across species (Supplementary Fig. 12). This observation is in agreement with MSH7 function also being required for genome stability in more distantly related species.

## Discussion

By 1952, it had become clear that corresponding bread wheat chromosomes derived from each subgenome were genetically very closely related, as observed through tetrasomy and nullisomy^43^. However the inability of these chromosomes to recombine during meiosis remained a paradox until a role for genetic suppressors was highlighted^44^. In this study, we report on the identification and functional validation of the key homoeologous chromosome pairing suppressor *Ph2*, through a combination of high-throughput exome and transcriptome sequencing of known mutants (*ph2a* and *ph2b*), cytogenetic analyses of both a 3DS deletion line series and independent EMS-induced mutants. We demonstrate that: (1) *Ph2* locates within a 14.3-Mb region ranging from 64.9 to 79.2 Mb on 3DS; (2) *TaMSH7-3D* is the only gene localised within this region that contains an EMS-derived SNP susceptible to affect protein sequence in *ph2b*; (3) an additional mutant of *TaMSH7-3D* recapitulates the *ph2* phenotype in regards to homologous and homoeologous recombination; and (4) we were able to exclude all previously proposed candidates for *Ph2* (not localised within the 14.3-Mb newly refined *Ph2* locus and not mutated in *ph2b*) except for *TaMSH7-3D* which had been fortuitously identified. Complementation of *Tamsh7-3D* mutants with a functional copy of the gene with its endogenous promoter would provide a direct evidence of this conclusion but plant regeneration after stable transformation remains recalcitrant in the currently available mutant backgrounds (Chinese Spring and Cadenza). Taken together, the provided data indicates *TaMSH7-3D* as being the causative gene for *Ph2*, thus contributing to the resolution of a half-a-century-old question.

TaMSH7 (*MutS homolog 7*) is a plant specific member of the DNA mismatch repair (MMR) family. These highly conserved proteins play an essential role in maintaining genome stability by assuring the initial step of the MMR pathway, i.e. recognition of base–base mismatches and insertion/deletion mispairs generated during DNA replication and recombination^45^. MSH7 forms a heterodimer with MSH2 and the protein complex allows specific recognition of single-base mismatches including G/G, G/A, A/A and C/A mispairs and to a lesser extent G/T, as shown by biochemical studies of Arabidopsis MSH2-MSH7 (MutS*ɣ*) complex^46–48^. The two heterodimeric complexes MSH2-MSH3 (MutS*β*) and MSH2-MSH6 (MutS*α*), present in yeast, animals and plants, have different mismatch recognition properties and abilities to support MMR. MSH2-MSH3 senses large (2-16 nucleotides) insertion/deletion loops and interstrand crosslinks, whereas MSH2-MSH6 recognises single-base mismatches, including oxidative mispairs (dihydro-8-oxoguanine), methylated mispairs (O^6^meG:T and O^6^meG:C) and small (1-2 nucleotides) insertion/deletion loops^49,50^. MSH7 appears to have arisen early in plant evolution, most likely via duplication and divergence from a MSH6-like gene present in a primitive plant^46,51^, with this extra DNA lesion recognition protein likely contributing to efficient repair of various DNA damage caused by constant environmental exposure, for which plants are naturally subjected^48^.

We show that the absence of a functional TaMSH7-3D induces a 5.5-fold genome-wide increase in chiasma frequency in a bread wheat/*Ae. variabilis* hybrid context (Fig. 3a, c), providing evidence that this protein acts as a key inhibitor of homoeologous recombination. This finding is in line with a previous study assessing how frequently alien chromatin of wild tomato (*Solanum lycopersicoides*) is introgressed into cultivated forms (*Solanum lycopersicum*) following *MSH7* silencing^38^. This study demonstrated a modest yet significant increase of 16.1% in recombination rate between these divergent chromosomes. In Arabidopsis, loss of AtMSH7 (*msh7* T-DNA insertion line) was observed to increase meiotic homologous recombination rate by 97% relative to wild-type at the subtelomeric 420 genetic interval as assessed using a fluorescent seed reporter line^52^. This data contrasts with a slight but significant reduction in genome-wide homologous recombination frequency observed for *Tamsh7-3D* in wheat (Fig. 3d–f)^32^. In some *Tamsh7-3D*/*ph2* wheat meiocytes, such a reduction in homologous recombination is associated with the presence of rare multivalents resulting from homoeologous recombination (Fig. 3e; Supplementary Table 5)^31^. This observation suggests that in a wild-type context, TaMSH7-3D plays a role in recombination partner choice (homologous vs homoeologous) likely through promoting destabilization of recombination intermediates established between homoeologous chromosomes. These intermediates could be less stable than those established between homologous sequences because of the presence of mismatches. A role for MMR proteins in recognising mismatches created in heteroduplex DNA (following DNA-strand exchange) and promoting dissociation of invading strand DNA – a process known as heteroduplex rejection – has indeed been reported^53^. In rice, MSH7 interacts with MEICA, an orthologue of FLIP known to be a partner of FIGL1^54^. FIGL1/FLIP is a conserved complex that regulates the strand invasion step of meiotic recombination^55^. Direct interaction between these two proteins is thus consistent with a role for TaMSH7-3D during this critical step. By preventing divergent DNA sequences from recombining, TaMSH7-3D would play a crucial role in assuring the diploid-like meiotic behaviour of polyploid bread wheat required for accurate chromosome segregation during meiosis. In diploid species, MSH7 may also be involved in limiting ectopic (non-allelic) recombination, a driver of highly deleterious chromosomal rearrangements, and could potentially provide an immediate advantage to newly formed allopolyploids by assuring meiotic stability and consequently, fertility.

Identification of the two main genes controlling homoeologous recombination in bread wheat, *TaZIP4-B2*^20^ and *TaMSH7-3D* (this study), now offers a possibility of deciphering their direct mode of actions and interactions. Recent data from G. Moore laboratory revealed that TaZIP4-B2 promotes homologous bivalent formation by preventing recombination intermediates established between homoeologous chromosomes from becoming crossovers^17,56^. In contrast to MMR proteins, there is no indication of ZIP4 involvement in promoting heteroduplex rejection following DNA-strand exchange between divergent sequences. This thus suggests that TaMSH7-3D and TaZIP4-B2 could act sequentially with different modes of action and consequently that homoeologous recombination is controlled by a multilayered mechanism in polyploid bread wheat. *ph1* was found to be twice as strong as *ph2*^44^ and an additive effect of these mutations in promoting homoeologous recombination has been reported, for example in wheat/*Aegilops* hybrids^33^. Combining *Tazip4-B2* and *Tamsh7-3D* mutations may therefore offer an opportunity to further improve the efficiency and ease of introgression of wild relative chromosomal segments into wheat, providing opportunities for the development of genetically unique and desirable wheat varieties. Exploitation of *Tazip4-B2* and *Tamsh7-3D* EMS-derived double mutants that are in the elite background Cadenza, are likely to be of particular interest to pre-breeders compared to previously available Chinese Spring mutants (*ph1b*, *ph2a*), as time required to move an introgression into a breeding relevant genotype is reduced. Additionally, the utilisation of point mutations is likely to avoid possible meiotic instability that can be induced by large chromosomal deletions.

*TaMSH7-3D* has two highly similar homoeologous copies on chromosomes 3A and 3B, *TaMSH7-3A* and *TaMSH7-3B*, with which it shares 97.77% and 97.96% identity, respectively (Supplementary Table 9). Because of possible functional redundancy and their genomic locality, it is reasonable to assume that *TaMSH7-3A* and *TaMSH7-3B* could correspond to the homoeologous recombination suppressors previously identified on 3AS^35,57^ and 3BS^58^. This also takes into consideration that the loss of both 3AS and 3DS (*Ph2*) was observed to result in a level of chiasma frequency between homoeologous chromosomes similar to that caused by the deficiency of 5B (*Ph1*)^35^. An interesting question is what could be the cause for differences in phenotypic severity observed between homoeologues (3DS > 3 AS > 3BS)^58^? As *TaMSH7-3A*, *TaMSH7-3B* and *TaMSH7-3D* show comparable RNA abundance in wheat anthers during early meiosis (Fig. 4), a difference derived from transcriptional level is unlikely. Although TaMSH7-3A, TaMSH7-3B and TaMSH7-3D proteins are very similar (>96.3% of sequence identity), mutation prediction algorithms have hinted to potentially deleterious amino acid substitutions between homoeologues (e.g. L877S in TaMSH7-3B and R855H in TaMSH7-3A). However, these predictions are indicative and require experimental validation. Additionally, a shared 28 bp deletion predicted to lead to a non-functional TaMSH7-3A protein in 14 wheat related accessions (Supplementary Figs. 9 and S10, Supplementary Table 11) indicates that *TaMSH7-3A* has degenerated into a pseudogene. This could potentially reflect progressive duplicated gene loss – which is particularly rapid for meiotic genes – following polyploidization events described in Angiosperms^59^. Generation of CRISPR/Cas9 mutants for one or more *TaMSH7* copies will allow confirmation of their relative impact on homoeologous recombination as well as determine their combinatorial effects, opening an opportunity to further refine exotic chromatin introgression into elite wheats.

Taking advantage of newly available genetic and bioinformatic resources, this research has answered a 50-year-old question on the causative agent for *Ph2* by identifying TaMSH7-3D. This work provides fundamental insights into the molecular control of meiotic recombination in allopolyploids and opens a path towards more efficient and flexible access to genetic diversity, a major bottleneck currently facing crop improvement.

## Methods

### Plant material and growth conditions

Plant material used in this study included the following: wild-type hexaploid wheat (*Triticum aestivum* cv. Chinese Spring and cv. Cadenza); Chinese Spring *ph2a* and *ph2b* mutants; 32 Chinese Spring 3D-deletion lines (from Svačina *et al*.^23^) and four Cadenza *Tamsh7-3D* mutant lines (*Tamsh7-3D* A467T (Cadenza0638); *Tamsh7-3D* D642N (Cadenza1114); *Tamsh7-3D* G463R (Cadenza1178); *Tamsh7-3D* Q605* (Cadenza2006) (Supplementary Table 3; www.wheat-tilling.com)). Plants were genotyped by PCR amplification using primers specific to wild-type or EMS mutant alleles (Supplementary Table 13). Wild-type cv. Chinese Spring, *ph2a*, *ph2b* and the 3D-deletion lines were crossed with rye (*Secale cereale*; RR, 2*n* = 14; var. Dankowski nove) to produce wheat/rye haploid hybrids (ABDR, *n* = 28). Wild-type cv. Cadenza and the *Tamsh7-3D* mutant lines were crossed with *Aegilops variabilis* (UUSS, 2*n* = 4*x* = 28) to produce wheat/*Ae. variabilis* haploid hybrids (ABDUS, *n* = 35). Plants were grown in a controlled-environment room with the following conditions: 16 h light/8 h night photoperiod at 20°C day and 15°C night, with 70% humidity.

### Cytological analysis

Young spikes were collected from 7 to 10-week-old plants and carefully dissected to isolate anthers. For each dissected floret, one of the three developmentally equivalent anthers was squashed in aceto-carmine staining solution and meiocytes visualised using a ZEISS Optima microscope. When meiocytes at metaphase I were identified (for chiasma frequency analysis) or other defined stages (RNA analysis), the two remaining anthers were either fixed in 100% ethanol/acetic acid 3:1 (v/v) for 48 h and then subsequently transferred to 70% ethanol or snap frozen in liquid N_2_ for later RNA-based analyses. Fixed anthers can eventually be stored at 4°C for a few months. For cytological analysis of meiocytes at metaphase I, pollen mother cells (PMCs) were released from the anther by crushing it on a slide in a drop of aceto-carmine staining solution. Anther debris was carefully removed, and a coverslip placed on the slide. The slides were then heated until separation of the chromosomes and aceto-carmine solution replaced by acetic acid 45%. Coverslips were then vertically pressed to spread out the chromosomes. Chromosome configurations of ~50 PMC per anther were analysed under a ZEISS Axio Observer Z1 inverted microscope. For each cell, the number of univalents, rod bivalents (pair of chromosomes linked by a unique chiasma), ring bivalents (pair of chromosomes linked by two chiasmata), trivalents (three chromosomes linked by two chiasmata) and quadrivalents (four chromosomes linked by three or four chiasmata) were counted. Frequency of chiasmata (the cytological manifestation of meiotic crossovers) was then calculated. Significant differences between mutant and corresponding wild-type control chiasma frequencies were assessed using Mann–Whitney tests adjusted for multiple comparisons.

### Exome capture and bioinformatic analysis

DNA was extracted from leaf material (10 cm long young, fresh and healthy leaves) from wild-type bread wheat cv. Chinese Spring, *ph2a* and *ph2b* by first snap-freezing the tissue in liquid nitrogen and crushing it into a fine powder (using knitting needle). The powder was resuspended in 600 μL of extraction buffer (0.1 M TRIS-HCl pH 8, 10 mM EDTA, 0.1 M NaCl, 1% sarkosyl, 2% polyvinyl-poplypyrrolidone (insoluble) and followed by addition of 600 μL phenol-chloroform-isoamyl alcohol (25:24:1) and hand mixing. The phases were separated by centrifugation in an Eppendorf centrifuge and the aqueous phase was re-extracted. After the second phenol-chloroform-isoamyl alcohol extraction, the aqueous phase was extracted once with an equal volume of chloroform. DNA was precipitated by the addition of 0.1 vol of 3 M sodium acetate (pH 4.8) and 1 vol of propan-2-ol. The DNA pellet was washed with 70% ethanol and resuspended in 30 μL TE buffer [10 mM TRIS-HC1 (pH 8.0), 1 mM EDTA] containing 40 μg/ml RNase A.

Purified DNA samples were sent to Arbor Biosciences (USA) for whole exome capture and sequencing. Genomic DNA was sonicated using a Q800R sonicator (Qsonica, CT, USA) and size-selected using SPRI beads to modal insert lengths of roughly 400 bp. Then 200 ng of the resulting processed DNA was converted to Illumina Truseq-style libraries using in-house chemistry and six cycles of dual-8bp-barcode indexing amplification. Target enrichment reactions were performed in singleplex using 750 ng of each library and the Arbor Wheat Exome Beta probe set. The enrichment procedure followed the standard myBaits version 4.0 manual (https://arborbiosci.com/wp-content/uploads/2018/04/myBaits-Manual-v4.pdf), but with 0.75 µL IDT xGen Universal Blocking Oligos (Integrated DNA Technologies, USA) in lieu of 0.5 µL Block A. Following capture clean-up as described in the myBaits user manual, libraries were submitted for 150 bp paired-end sequencing on a partial NovaSeq S4 lane (Illumina). Following sample de-multiplexing using both barcodes per library, read pairs were taken to bioinformatic analysis.

Read qualities were inspected with FASTQC version 0.11.4^60^ before and after quality and adapter trimming. For the exome capture data, trimming was achieved with Trimmomatic version 0.36^61^, using the following parameters: -phred33 LEADING:5 TRAILING:5 SLIDINGWINDOW:4:20 MINLEN:50.

Cleaned exome capture reads were aligned to the CS Ref v1.0 by Bowtie2 version 2.3.0^62^ allowing a 2% mismatch rate with the following parameters: --end-to-end --very-sensitive --n-ceil L,0,0.1 --rdg 3,3 --rfg 3,3 --no-unal --mp 6,6 --np 4 --no-mixed --score-min L,0,-0.12. After alignment, PCR duplicates were detected and removed from bam files using an in-house Java application Wheatbio.jar (https://github.com/CroBiAd/TILLinG-mutants).

After read alignment, a pileup file was generated from the bam files of the exome capture data using the mpileup command (–min-MQ 20 -B -f) in samtools version 1.4.1^63^. The pileup file was subsequently used to identify genome-wide SNPs and indels. To determine the effect of the detected polymorphisms the CS Ref v1.0 annotation, specifically the high-confidence gene models, was relied upon. To avoid calling false positive polymorphisms, we demanded that each SNP or indel position was supported by a read depth of ≥4. To predict the consequences of mutations on protein sequences, we used SNPeff version 4.3^64^ and the annotation from CS Ref v1.0 choosing the longest predicted splice-form.

### RNA sequencing and processing

Anthers cytologically determined to be at metaphase I or earlier were pooled for RNA extraction according to Tucker et al.^65^. These purified early-meiotic anther RNA samples derived from *ph2b* and Chinese Spring were submitted to the Australian Genome Research Facility (AGRF, Australia) for library preparation and sequencing on the Illumina NovaSeq 6000 instrument. Stranded cDNA was generated from poly-adenylated RNA by TruSeq stranded mRNA library kits (Illumina). Samples were sequenced to give 150 bp paired-end reads aiming at around 130 Mill reads/sample.

RNASeq raw data was processed with fastp version 0.19.7^66^ within minimum length requirement of 60, trimming of poly G and removal of the first 10 bases. After trimming we had 144,818,345 clean paired-end reads for *ph2b* replicate 1 and 141,336,841 reads for *ph2b* replicate 2. For the wild-type Chinese Spring we obtained 130,912,185 and 135,248,954 reads for the two replicates, respectively. Trimmed RNASeq reads were aligned using STAR version 2.5.3^67^ to CS Ref v1.0 with the following parameters: --outFilterMismatchNoverLmax 0.02 --outFilterMatchNminOverLread 0.98 --outFilterMultimapNmax 5 --outFilterMultimapScoreRange 0 --outFilterScoreMinOverLread 0 --alignEndsType Local --alignIntronMax 10000 --alignMatesGapMax 10500 --alignSoftClipAtReferenceEnds No --outSJfilterOverhangMin 35 20 20 20 --outSJfilterCountTotalMin 10 3 3 3 --outSJfilterCountUniqueMin 5 1 1 1.

### Genetic diversity and phylogenetic analysis

The MSH7-3A and 3B sequences were used as queries in BLASTN searches against available genomic sequence data generated by the Wheat Initiative’s 10+ Genomes project (https://webblast.ipk-gatersleben.de/wheat_ten_genomes/) and for the UK varieties https://wheatis.earlham.ac.uk//grassroots-portal/blast. Australian varieties were inspected for variation in MSH7-3A and 3B in DAWN (http://crobiad.agwine.adelaide.edu.au/dawn/jbrowse/). Sequences for Cadenza and Paragon were retrieved and trimmed to exon2 for CLUSTALW alignment. Public databases were searched for the presence of the 28 bp deletion by either using the full MSH7-3A sequence or for NCBI-SRA data with a 300 bp subsequence covering the deleted region. In addition we queried DAWN^42^ at position chr3A:87373944..87374105 for the presence of the deletion in Bioplatforms Australia sequenced varieties (https://data.bioplatforms.com/organization/bpa-wheat-cultivars). Pedigree information was retrieved from GRIS (http://wheatpedigree.net/) when available or the literature otherwise.

Grass sequences homologous to MSH7 were identified by BLAST searches against a range of databases (Supplementary Table 12). Whenever gene models were non-existent or incomplete, putative homologs were manually derived from genomic DNA, and their protein sequences deduced.

DNA and protein sequences were aligned by MUSCLE in Geneious version 10 and percentage identities calculated. The unrooted phylogenetic tree was inferred using PhyML v20160115 ran with model JTT and parameters: --nclasses 4 –f m –alpha e –pinv e –bootstrap 100 –o tlr^68^. Branch supports are computed out of 100 bootstrapped trees.

### Reporting summary

Further information on research design is available in the Nature Research Reporting Summary linked to this article.

## Supplementary information

Supplementary Information

Peer Review File

Reporting Summary

Description of Additional Supplementary Files

Supplementary Data 1

## Data Availability

Data supporting the findings of this work are available within the paper and its Supplementary Information files. A reporting summary for this article is available as a Supplementary Information file. The datasets and plant materials generated and analysed during the current study are available from the corresponding author upon request. Exome capture and RNA Seq data have been deposited in the NCBI SRA database under BioProject PRJNA648242. Exome capture data have accession numbers SRR12315376 and SRR12315377, RNAseq data are under SRR13364311 and SRR13364312. The genomic sequences of *TaMSH7-3D* from *ph2b* mutant and *TaMSH7-3A* from Cadenza are available in Supplementary Data 1. Source data are provided with this paper.

## Source data

Source Data

## Code availability

Code is available at GitHub [https://github.com/CroBiAd/TILLinG-mutants].
