# Supplementary material for: Ph2 encodes the mismatch repair protein MSH7-3D that inhibits wheat homoeologous recombination
Source: Nat Commun. 2021 Feb 5;12:803. doi: 10.1038/s41467-021-21127-1 (PMC7865012; doi:10.1038/s41467-021-21127-1)
Supplement: Supplementary file 1 — Supplementary Information [file 41467_2021_21127_MOESM1_ESM.pdf]

Ph2 encodes the mismatch repair protein MSH7-3D that inhibits wheat homoeologous recombination

Serra *et al.*

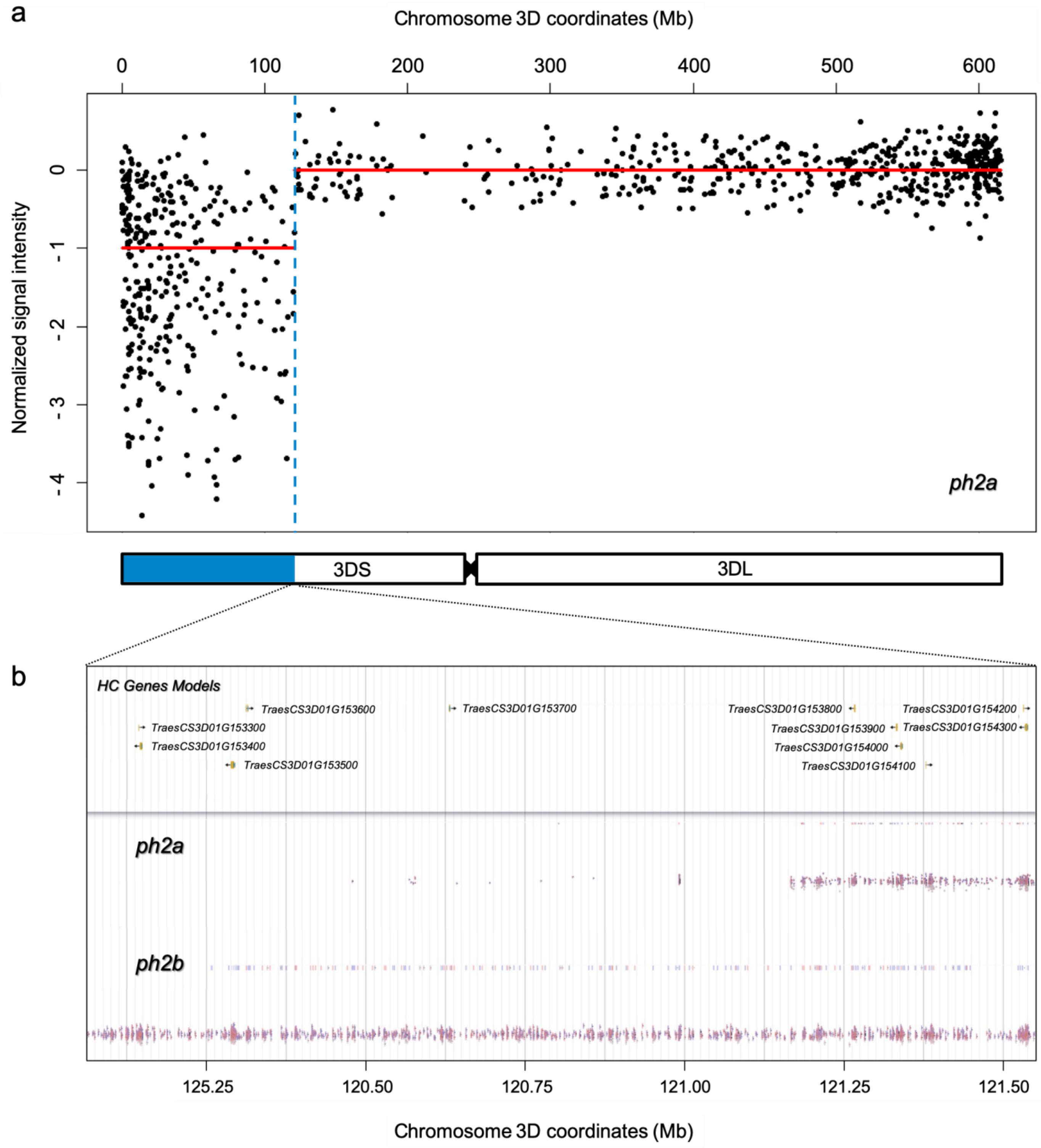

a
Chromosome 3D coordinates (Mb)
0
100
200
300
400
500
600
Normalized signal intensity
0
-1
-2
-3
-4
ph2a
3DS
3DL
b
HC Genes Models
TraesCS3D01G153300
TraesCS3D01G153400
TraesCS3D01G153500
TraesCS3D01G153600
TraesCS3D01G153700
TraesCS3D01G153800
TraesCS3D01G153900
TraesCS3D01G154000
TraesCS3D01G154100
TraesCS3D01G154200
TraesCS3D01G154300
ph2a
ph2b
125.25
120.50
120.75
121.00
121.25
121.50
Chromosome 3D coordinates (Mb)

Supplementary Figure 1. Identification of *ph2a* deletion breakpoint on chromosome 3D by high-density SNP genotyping and exome capture of *ph2a* mutant. (a) Normalized signal intensity plot of 828 markers from Affymetrix 35K SNP array located along chromosome 3D. *ph2a* signal intensities were normalized using wild-type Chinese Spring values for each marker. Each dot represents an individual marker. The deletion breakpoint of *ph2a* (dotted blue line) is detected at ~121 Mb (between markers AX-178057815 and AX-178057206 at the coordinates 120.722.379 and 121.539.725, respectively). Red lines represent normalized signal intensity means of the markers located upstream (mean = - 1.352) and downstream (mean = 0.009) of the deletion breakpoint. **(b)** Distribution of aligned captured reads from *ph2a* and *ph2b* visualized in JBrowse. The upper track shows the location and gene identifiers of high confidence genes on chromosome 3D from 125.06 to 121.55 Mb. The two tracks below show aligned reads of *ph2a* and *ph2b* as extended read clouds for each genotype. *ph2a* deletion breakpoint is upstream of gene Traes3D01G153800 at approximately position 121.16 Mb.

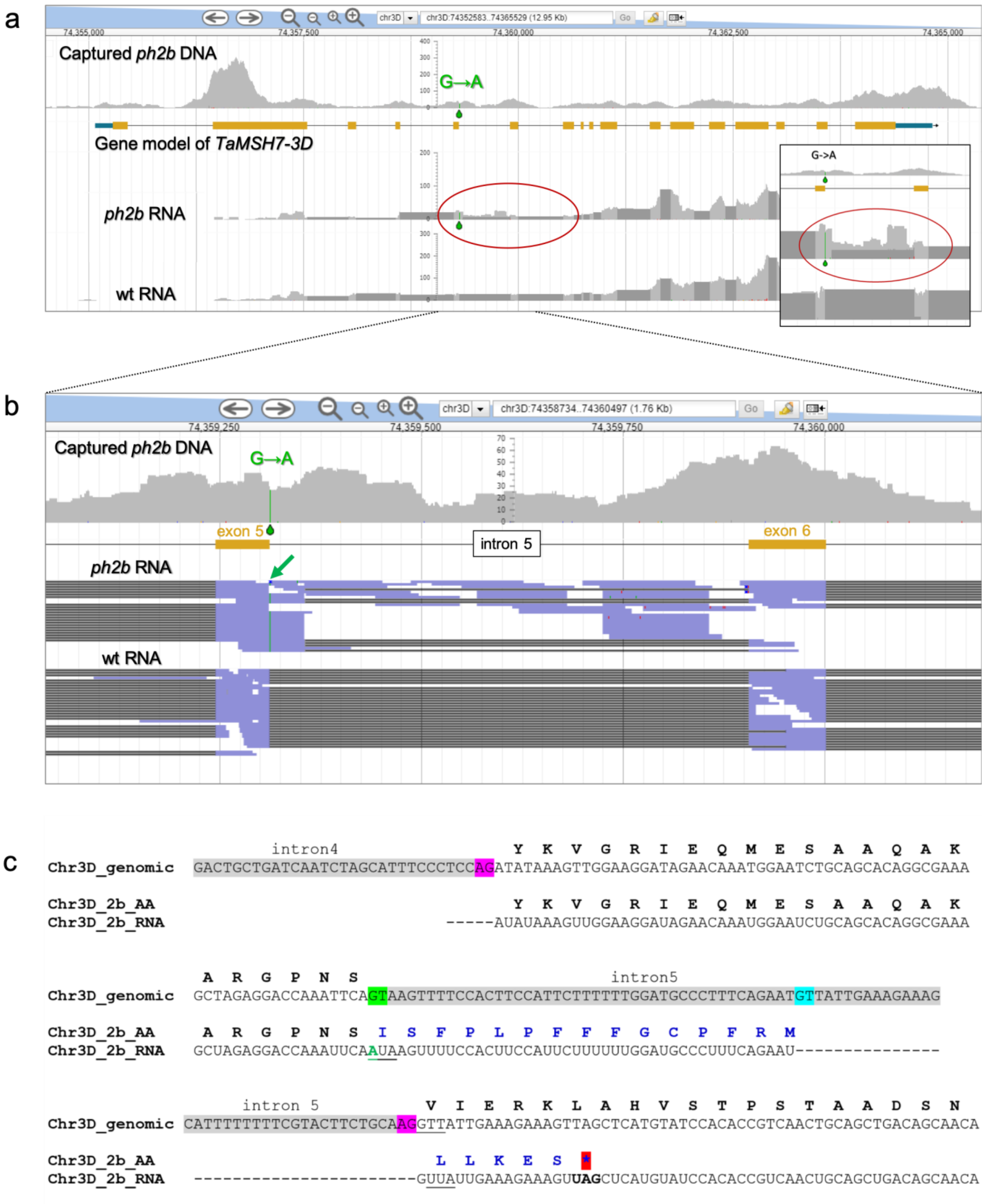

a
chr3D
chr3D:74352583..74365529 (12.95 Kb)
Go
74,355,000
74,357,500
74,360,000
74,362,500
74,365,000
Captured ph2b DNA
G→A
Gene model of TaMSH7-3D
ph2b RNA
wt RNA
G->A
b
chr3D
chr3D:74358734..74360497 (1.76 Kb)
Go
74,359,250
74,359,500
74,359,750
74,360,000
Captured ph2b DNA
G→A
exon 5
intron 5
exon 6
ph2b RNA
wt RNA
c
intron4
Y K V G R I E Q M E S A A Q A K
Chr3D_genomic GACTGCTGATCAATCTAGCATTTCCCTCCAGATATAAAGTTGGAAGGATAGAACAAATGGAATCTGCAGCACAGGCGAAA
Chr3D_2b_AA Y K V G R I E Q M E S A A Q A K
Chr3D_2b_RNA -----AUAUAAAGUUGGAAGGAUAGAACAAAUGGAAUCUGCAGCACAGGCGAAA
A R G P N S
intron5
Chr3D_genomic GCTAGAGGACCAAATTCAGTAAGTTTTCCACTTCCATTCTTTTTTGGATGCCCTTTCAGAATGTTATTGAAAGAAAG
Chr3D_2b_AA A R G P N S I S F P L P F F F G C P F R M
Chr3D_2b_RNA GCUAGAGGACCAAAUUCAAUAAGUUUUCCACUUCCAUUCUUUUUUGGAUGCCCUUUCAGAAU--------------
intron 5
V I E R K L A H V S T P S T A A D S N
Chr3D_genomic CATTTTTTTTCGTACTTCTGCAAGGTTATTGAAAGAAAGTTAGCTCATGTATCCACACCGTCAACTGCAGCTGACAGCAACA
Chr3D_2b_AA L L K E S *
Chr3D_2b_RNA ----------------------GUUAUUGAAAGAAAGUUAGCUCAUGUAUCCACACCGUCAACUGCAGCUGACAGCAACA

Supplementary Figure 2. The G to A transition within *TaMSH7-3D* gene in *ph2b* affects transcript splicing. (a) Exome capture read coverage and RNASeq coverage of *ph2b* and wild-type across the *TaMSH7-3D* gene. The top track shows the coverage distribution of aligned exome capture reads of *ph2b.* The G → A mutation at the splice junction is indicated by a green line and droplet. The track below displays the gene model of *TaMSH7-3D* with exons represented by yellow rectangles and introns by black lines. The two lower tracks show the coverage distribution of RNA reads of *ph2b* and Chinese Spring wild-type, dark shading indicates spliced reads. The mutated splice junction leads to changes in the splicing pattern when compared to wild-type; highlighted by the red oval. Close-up of intron 5 is shown in the right panel inset. **(b)** Exome capture distribution and RNASeq alignment of *ph2b* and wild-type across exons 5 and 6 of the *TaMSH7-3D* gene. The G → A mutation at the splice junction is identified by the green arrow in the RNA data. Aligned RNA reads of *ph2b* and Chinese Spring wild-type are shown in purple. Spliced reads are connected by black lines across the intron. Wrongly spliced reads across intron 5 are visible in *ph2b* but absent from wild-type. **(c)** Alignment of portions of *TaMSH7-3D* genomic reference sequence and corresponding RNA sequences from *ph2b*. Deduced amino acid sequence is shown above the nucleotide sequences. The left splice junction of intron 5 (in green) is lost in *ph2b*; hence the transcript is spliced at a downstream GT (in blue) and then fused with the correct junction at the next exon (in pink). The *ph2b TaMSH7-3D* transcript thus extends into the intron and also gains a frameshift (+1) which leads to a prematurely terminated protein due to a now in-frame STOP codon (*, in red).

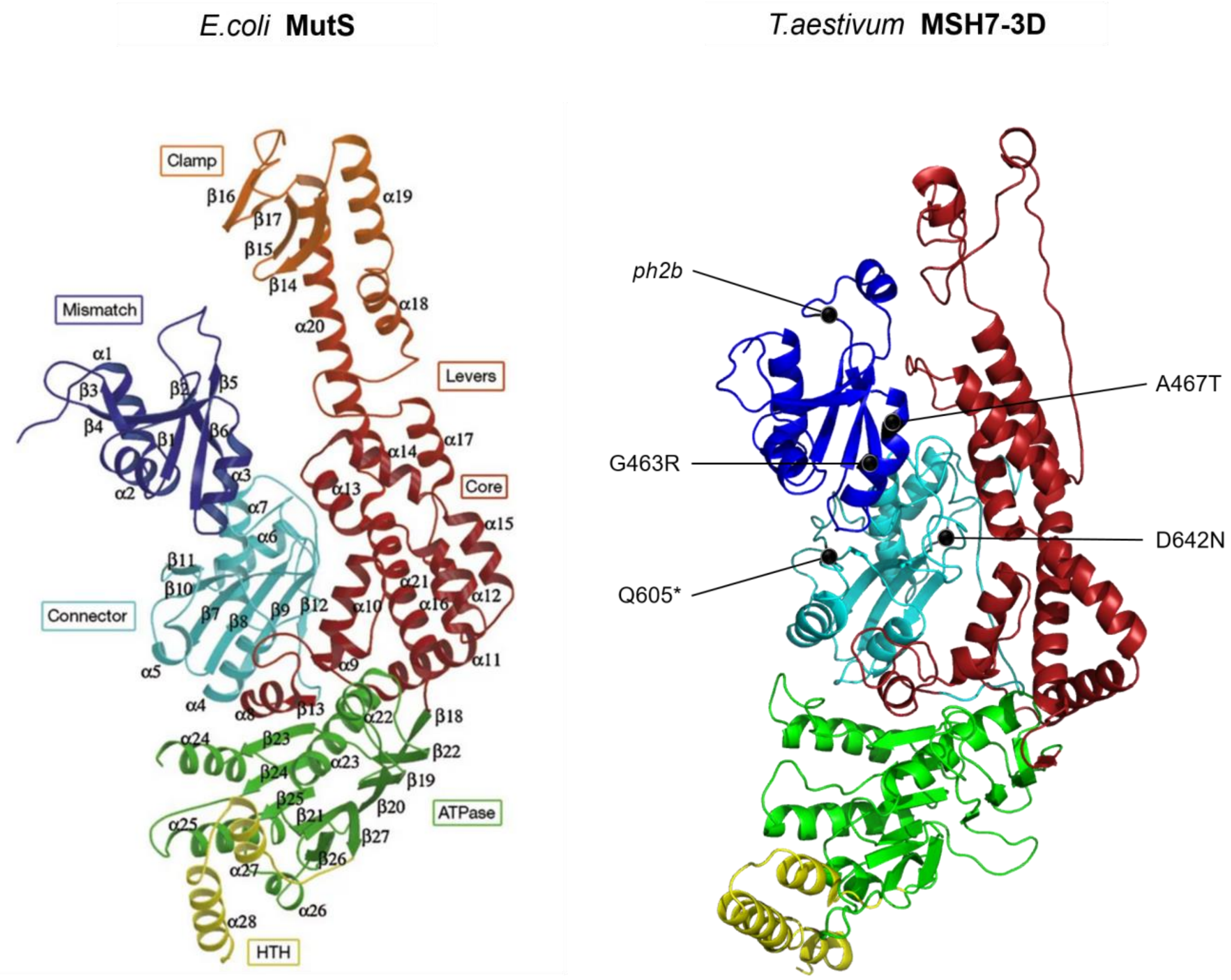

Supplementary Figure 3. Location of *Tamsh7-3D* mutations on *T. aestivum* MSH7-3D crystal structure. *E. coli* MutS and *T. aestivum* MSH7-3D mismatch-binding domains are coloured dark blue, the connector domains light blue, the core domains (that embrace but do not touch the DNA) and levers from red to orange, the clamp domain orange, the ATPase domains green and the helix-turn-helix (HTH) domains involved in dimer contacts yellow. The *E. coli* MutS crystal structure is from Lamers *et al.* [1].

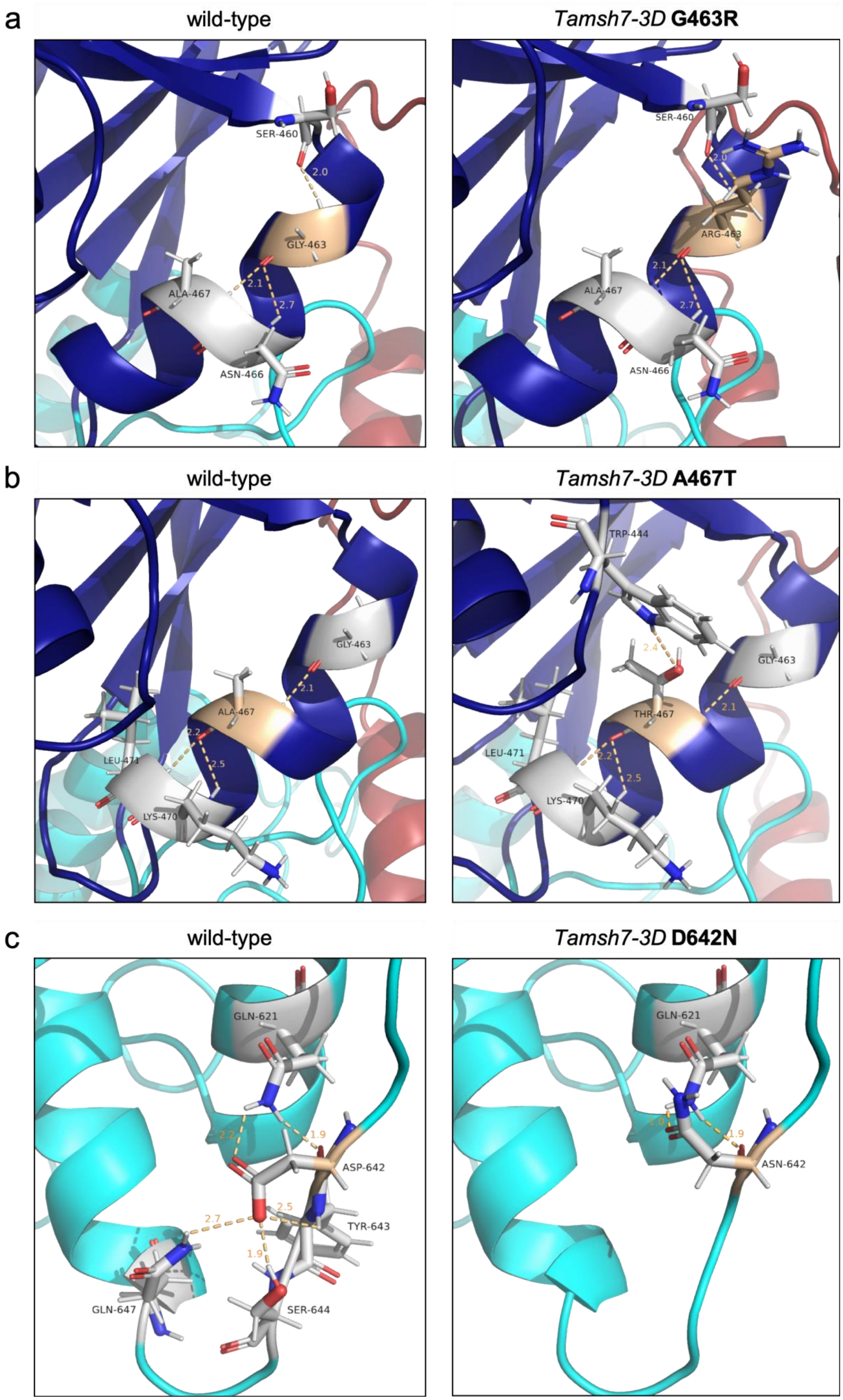
a
wild-type
Tamsh7-3D G463R
SER-460
2.0
GLY-463
ALA-467
2.1
2.7
ASN-466
ARG-463
b
wild-type
Tamsh7-3D A467T
GLY-463
ALA-467
2.1
2.2
2.5
LEU-471
LYS-470
TRP-444
2.4
THR-467
c
wild-type
Tamsh7-3D D642N
GLN-621
2.2
1.9
ASP-642
2.7
2.5
TYR-643
1.9
GLN-647
SER-644
ASN-642

Supplementary Figure 4. EMS-mutations and effects on local protein structure. The figures show a close up of the wild-type and mutated amino acid in the three Cadenza non-synonymous substitution mutants: (a) *Tamsh7-3D* G463R (Cadenza1178), (b) *Tamsh7-3D* A467T (Cadenza0638) and (c) *Tamsh7-3D* D642N (Cadenza1114). The affected amino acids are highlighted in beige and their side chains are visible. Mutations were created in PyMol and the rotamers with highest probability are shown (G463R: 16.6% of 17 possible rotamers; A467T: 96.6% of 2 rotamers; D642N: 30.4% of 15 rotamers). Polar interactions between amino acid side chains are shown in the figures, with distances given in Angstrom. Atoms are colour-coded: C-grey, H-white, N-blue, O-red. **(a)** Investigation of the interaction of the both wild-type and mutated amino-acid showed that Gly-463 resides within 5Å to [459]ISES-IDNAV [468]; when mutated to Arg, W-444 also is included; however, polar contacts stay the same. **(b)** Ala-467 is within 5Å to L-430, L-441, W-444, I-459 and [463]GIDN-VEKELL[472]; this neighborhood stays unchanged for Thr-467 except for the creation of a polar contact with W-444. **(c)** Asp-642 lies within 5Å of Q-621, [640]AL-YS..Q…I[651]; replacement with Asn-642 results in an additional contact to V-624 the loss of several polar contacts as seen in the figure.

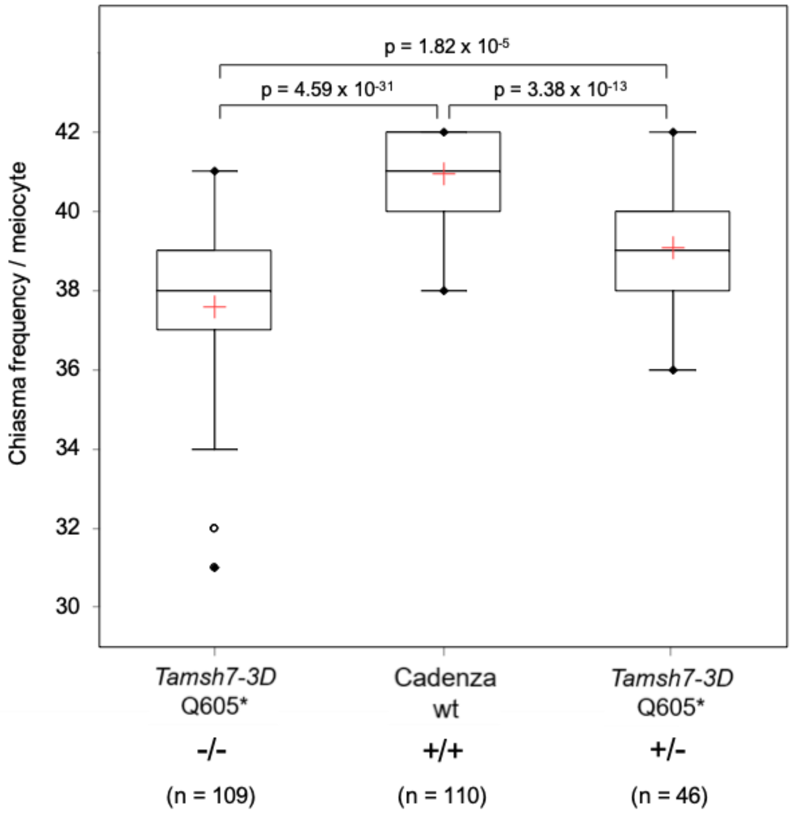

Supplementary Figure 5. Meiotic chromosome phenotype at metaphase I of wild-type and *Tamsh7-3D* Q605* homozygous and heterozygous plants. Box plots showing minimum, first quantile, median (horizontal middle line), third quantile and maximum count of chiasma frequency per meiocyte. Mean values are represented by red crosses. n = number of cells examined. Two-sided Mann-Whitney tests adjusted for multiple comparisons were performed to test for significant differences. Source data are provided as a Source Data file.

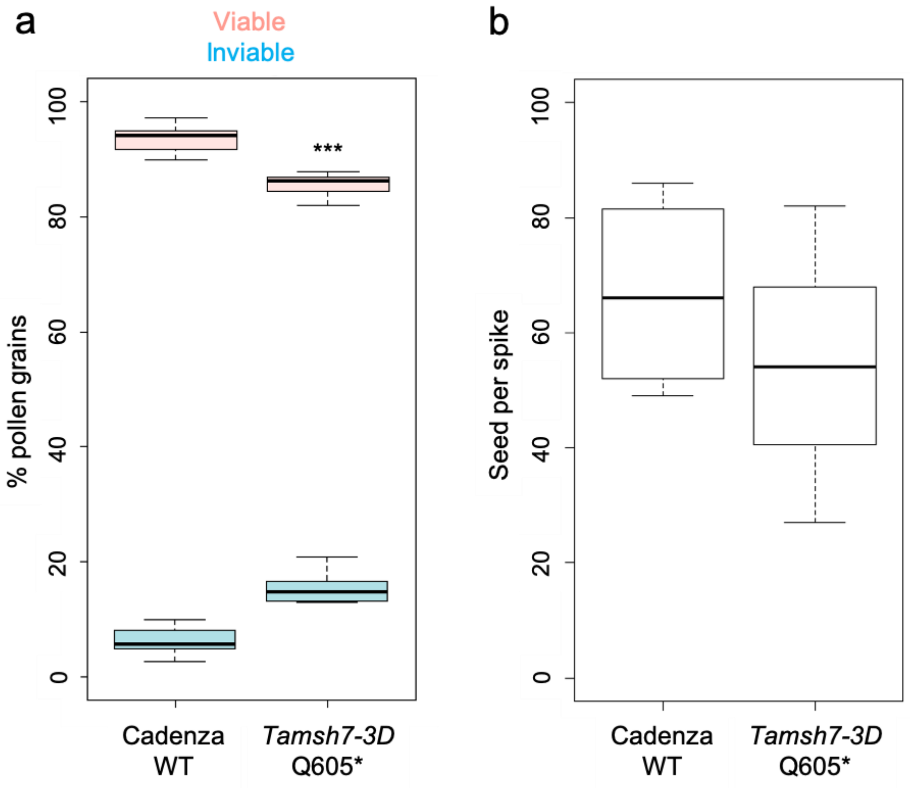

Supplementary Figure 6. Seed and pollen fertility in wild-type and *Tamsh7-3D* Q605*. (a) Boxplots showing minimum, first quantile, median (horizontal middle line), third quantile and maximum count of pollen viability using Alexander staining. Pollen viability from 8 anthers per genotype were assessed using Alexander staining of at least 600 pollen grains from mature flowers. *** two-sided t-test *p* value = 6×10^{-6} **(b)** Boxplots showing minimum, first quantile, median (horizontal middle line), third quantile and maximum count of seed set per spike for the indicated genotypes. Seed number from the first 6 spikes of 4 separate plants per genotype were counted.

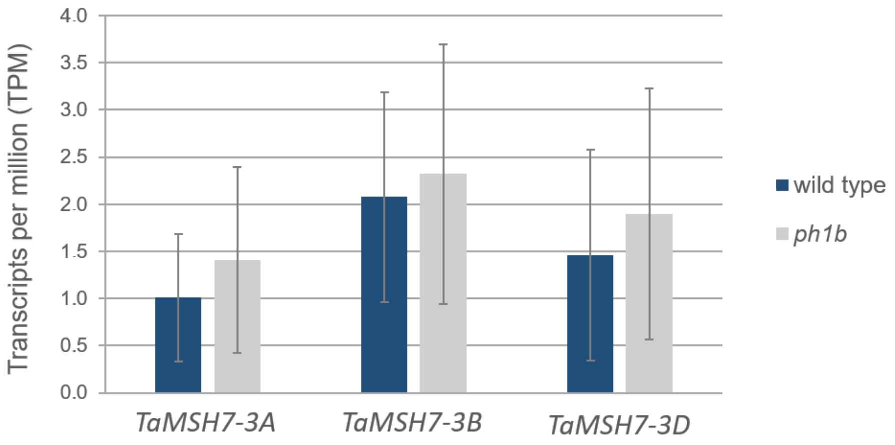

Supplementary Figure 7. Relative expression of *TaMSH7-3A*, *TaMSH7-3B* and *TaMSH7-3D* in *Triticum aestivum* cv. Chinese Spring in presence (wild-type) **or absence** (*ph1b*) **of the *Ph1* locus.** RNA-seq was performed on anthers containing pollen mother cells at early meiotic prophase I, for which there were three replicates per genotype. Histogram bars represent the mean ± standard error of mean. Data derived from Martin *et al.* [2].

```
MSH7-3A      MQPRRQQQQSILPFLHPRQGPAQEAPGAGRTPERPPRPPAASSVDGIMERLVRPPSQGRN      60
MSH7-3B      MQPRR-QQQSLLSFLHPRQSPAQEALGAGTPPERPPRPPAASSVDGIMERLVRPPSQGRN      59
MSH7-3D      MQPRR-QQQSIRSFLHPRPSPAQEASGAGTTPERPPRPPAASSVDGIMERLVRPPSQGRN      59
             ***** ****:  ***** .***** ***  *****************************

MSH7-3A      KDAAQIRNAERALPGRNEDTSNEQPSASFPVRHNGKYSRGTVLFAEHSTDTTPPQEPLKF      120
MSH7-3B      KDAAQIRNAERALPGKNEDTSNEQPSASFPVRYNGKYSRGTVLFAEHSTDTTPPQEPLKF      119
MSH7-3D      KDATQIRNAERALPGKNEDTSNEQPSASFPVRYNGKYSRGTVLFAEHSTDTTPPQEPLKF      119
             ***:***********:****************:***************************

MSH7-3A      SARSSTDEFVRASTLFPELGYP-TLLQECPKKLSSECPSNQYVQANSVFEAFDVQTPSQD      179
MSH7-3B      SARSSTDEFVKASTLFPELGSDQTLLQECPKKLSSECPSNQYVQANSVFEAFDVQTPSQD      179
MSH7-3D      SARSSTDEFVRASTLFPEIGSDQTLLQECPKKLSSECPSNQYVQANSVFEAFDVQTPSQD      179
             **********:*******:*   *************************************

MSH7-3A      PLKRIFSGPFHGADTPLSEYRSYPIPLQHPSKKSSSGSSSGEYLRAVTPLGLDSNDTPIA      239
MSH7-3B      PLKRIFSGPFHGADTPLSEYRSYPIPLQHPSKNLSSGSSSGEYLRAVTPLGLDSNDTPTA      239
MSH7-3D      PLKRIFSGPFHGADTPLSEYRSYPIPLQHPSKKLSSGSSSGEYLRAVTPLGLDSNDTPTA      239
             ********************************: ************************ *

MSH7-3A      KHSKKLFSGSSDHSYIKATNLFPEFDSNGTPLQNHLNKFSVSMNGKHIGAPATLFPELDS      299
MSH7-3B      KHSKKLFSGSSDHSYIKATNLFPEFDSNGTPLQNHSNKFSVSMNGKHIGAAATLFPELDS      299
MSH7-3D      KHSKKLFSGSSDHSYIKATNLFPEFDSNGTPLQNHSNKFSVSMNSKNIGAPATLFPELDS      299
             *********************************** ********.*:*** *********

MSH7-3A      VLLKPETPVTRAVAPRGKRVQQDQCMTANNSQSPLWGSNKKVKSAHCSPAGKMAHDEMAE      359
MSH7-3B      VLLKPETPVTRAVAPRGKRVQQDQRMTANNSQSPLWGSNKKVKSAHCSPPGKMVHDEMAE      359
MSH7-3D      VLLKPETPVTQAVAPRGKRVQQDQCMTANNCQSPLWGSNKKVKSAHCSPAGKMVHDEMAE      359
             **********:************* *****.****************** ***.******

MSH7-3A      SARSKFEWLNPLNIRDANKRRPDDPLYDKRTLFIPPDALRKMSTSQKQYWTIKCKYMDVL      419
MSH7-3B      SARSKFEWLNPLNIRDANKRRPDDPLYDKRTLFIPPDALRKMSTSQKQYWSIKCKYMDVL      419
MSH7-3D      SARSKFEWLNPLNIRDANKRRPDDPLYDKRTLFIPPDALRKMSTSQKQYWTIKCKYMDVL      419
             **************************************************:*********
                                 MutS domain I
MSH7-3A      LFFKVGKFYELYEVDAEIGQKELDWKMTISGVGKCRQVGISESGIDDAVEKLLARGYKVG      479
MSH7-3B      LFFKVGKFYELYEVDAEIGQKELDWKMTISGVGKCRQVGISESGIDDAVEKLLARGYKVG      479
MSH7-3D      LFFKVGKFYELYEVDAEIGQKELDWKMTISGVGKCRQVGISESGIDNAVEKLLARGYKVG      479
             **********************************************:*************

MSH7-3A      RIEQMESAAQAKSRGPNSVIERKLAHVSTPSTAADSNIGPDAVHLLALKEVTLASNGSRV      539
MSH7-3B      RIEQMESAAQAKARGPNSVIERKLAHVSTPSTAADSNIGPDAVHLLALKEVTLASNGSRL      539
MSH7-3D      RIEQMESAAQAKARGPNSVIERKLAHVSTPSTAADSNIGPDAVHLLALKEVTLASNGSRV      539
             ************:**********************************************:

MSH7-3A      YGFAFLDYAALKIWVGSLQDDDSSAALGALLVQVSPREIIYESSGLSRESRKSMIKYASA      599
MSH7-3B      YGFAFLDYAALKIWVGSLQDDDSSAALGALLVQVSPREIIYESSGLSRESRKSMIKYASA      599
MSH7-3D      YGFAFLDYAALKIWVGSLQDDDSSAALGALLVQVSPREIIYESSGLSRESRKSMIKYASA      599
             ************************************************************
                                 MutS domain II
MSH7-3A      GSVKMQLTPLPGTGFSDASQIQMLVHSKGYFKASTDSWLSALDYSVNRDAVIFALGGLIG      659
MSH7-3B      GSVKMQLTPLPGTDFSDASQIQMLAHSKGYFKASTDSWLSALDYSVNRDAVICALGGLIG      659
MSH7-3D      GSVKMQLTPLPGTDFSDASQIQMLVHSKGYFKASTDSWLSALDYSVNQDAVICALGGLIG      659
             *************.**********.**********************:**** *******

MSH7-3A      HLTRLMLDDALKNGEVLPYNVYQTCLRMDGQTLVNLEIFGNNFDGGSSGTLYKHLNHCIT      719
MSH7-3B      HLTRLMLDDALKNGEVLPYNVYQTCLRMDGQTLVNLEIFGNNFDGGSSGTLYKHLNHCIT      719
MSH7-3D      HLTRLMLDDALKNGEVLPYNVYQTCLRMDGQTLVNLEIFGNNFDGGSSGTLYKHLNHCIT      719
             ************************************************************

MSH7-3A      ASGKRLLRRWICHPLKDVDAINRRLDVVEGFIQHCGVGSITLYYLRKIPDLERLLGRIRS      779
MSH7-3B      ASGKRLLRRWICHPLKDVDAINRRLDIVEGFIQHCGVGSITLEHLRKIPDLERLLGRVRS      779
MSH7-3D      ASGKRLLRRWICHPLKDVDAINRRLDIVEGFIQHCGVGSVTLEHLRKIPDLERLLGRVRS      779
             **************************:************:** :*************:**
                                 MutS domain III
MSH7-3A      TVGLTSAVLLPFVGEKILKRRIKTFGMLIKGLRVGIDLLSALRRDDHGIPALSKSVDIPT      839
MSH7-3B      TVGLTSAVLLPFVGEKILKRRIKTFGMLIKGLRVAIDLLSALHREDHGIPALSKSVDIPT      839
MSH7-3D      TVGLTSAVLLPFVGEKILKRRIKTFCMLIKGLRVAIDLLSALRREDHGIPALSKSVDIPT      839
             ************************* ********.*******:*:***************
```

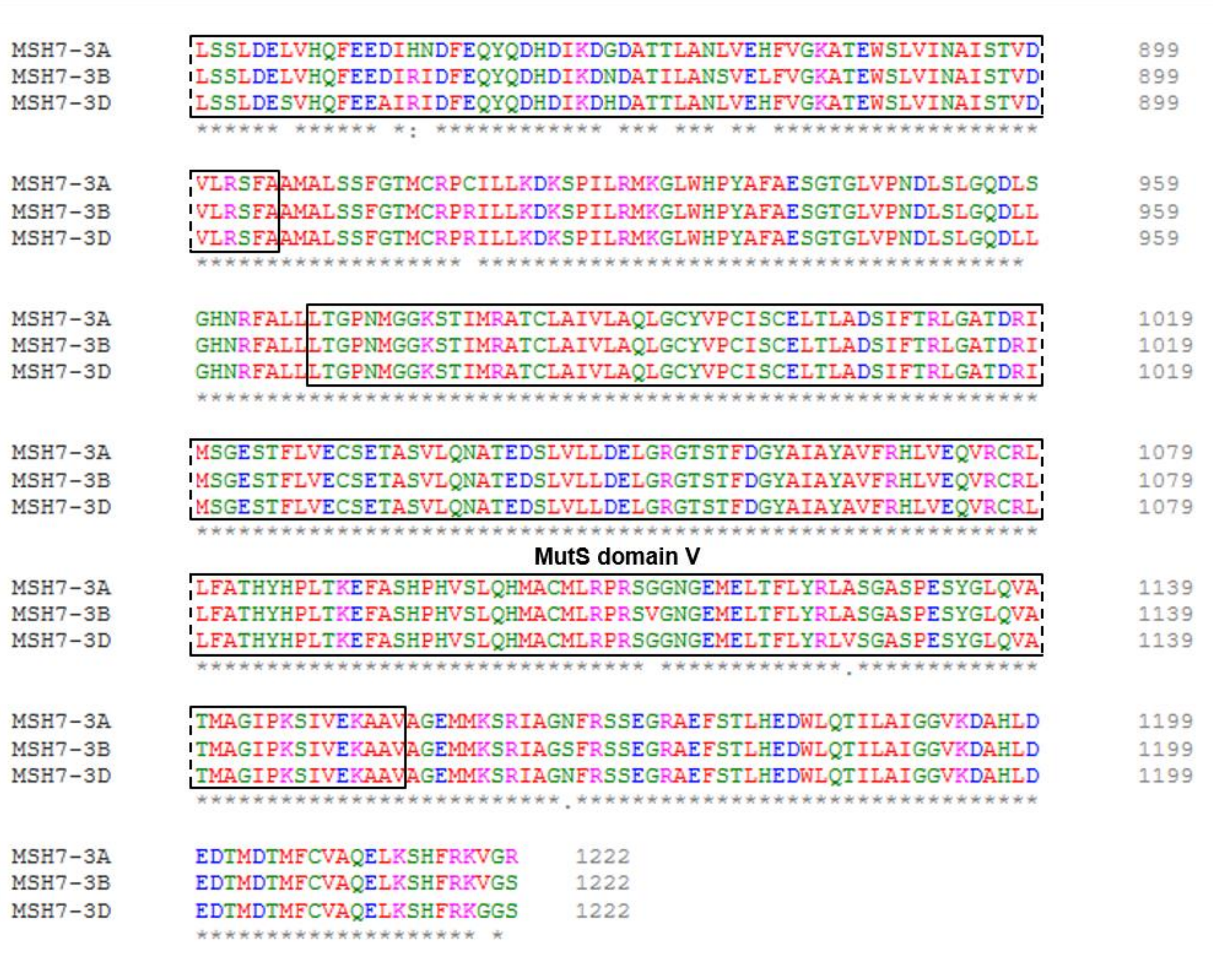

Supplementary Figure 8. Alignment of TaMSH7-3A, TaMSH7-3B and TaMSH7-3D protein sequences. Dashes denote gaps. Amino acid positions are shown on the right. Stars below the alignment denote identical amino acids across the three sequences. Boxes highlight the conserved protein domains [3]: (1) MutS domain I (amino acid: 405-515): N-terminal mismatch-recognition domain; (2) MutS domain II (amino acid 525-672): connector domain; (3) MutS domain III (amino acid 689-905): core domain composed of two separate subdomains that join together to form a helical bundle; (4) MutS domain V (amino acid 967-1154): ATPase domain containing a Walker A motif.

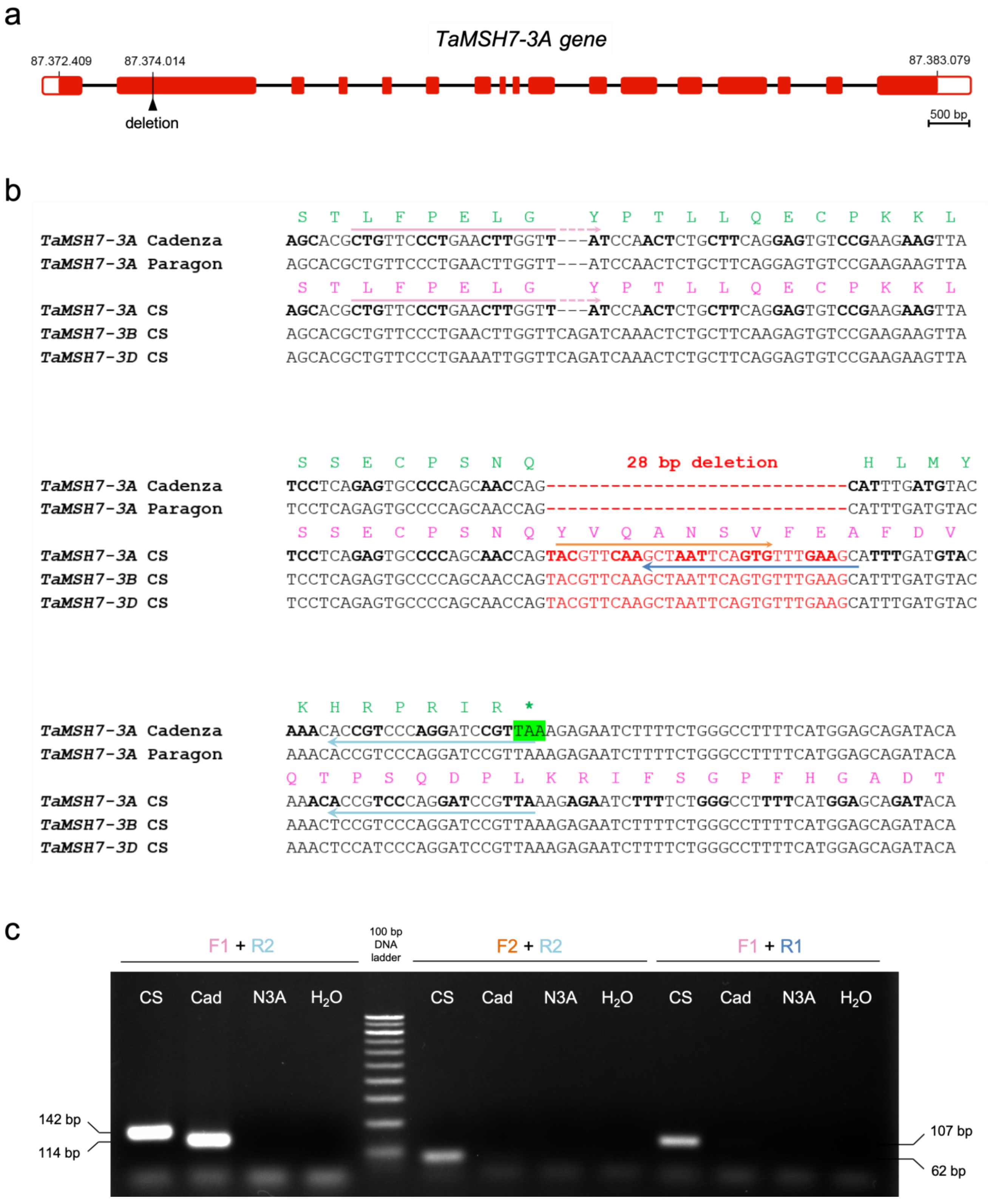
a
TaMSH7-3A gene
87.372.409
87.374.014
87.383.079
deletion
500 bp
b
S T L F P E L G Y P T L L Q E C P K K L
TaMSH7-3A Cadenza AGCACGCTGTTCCCTGAACTTGGTT---ATCCAACTCTGCTTCAGGAGTGTCCGAAGAAGTTA
TaMSH7-3A Paragon AGCACGCTGTTCCCTGAACTTGGTT---ATCCAACTCTGCTTCAGGAGTGTCCGAAGAAGTTA
S T L F P E L G Y P T L L Q E C P K K L
TaMSH7-3A CS AGCACGCTGTTCCCTGAACTTGGTT---ATCCAACTCTGCTTCAGGAGTGTCCGAAGAAGTTA
TaMSH7-3B CS AGCACGCTGTTCCCTGAACTTGGTTCAGATCAAACTCTGCTTCAAGAGTGTCCGAAGAAGTTA
TaMSH7-3D CS AGCACGCTGTTCCCTGAAATTGGTTCAGATCAAACTCTGCTTCAGGAGTGTCCGAAGAAGTTA
S S E C P S N Q 28 bp deletion H L M Y
TaMSH7-3A Cadenza TCCTCAGAGTGCCCCAGCAACCAG----------------------------CATTTGATGTAC
TaMSH7-3A Paragon TCCTCAGAGTGCCCCAGCAACCAG----------------------------CATTTGATGTAC
S S E C P S N Q Y V Q A N S V F E A F D V
TaMSH7-3A CS TCCTCAGAGTGCCCCAGCAACCAGTACGTTCAAGCTAATTCAGTGTTTGAAGCATTTGATGTAC
TaMSH7-3B CS TCCTCAGAGTGCCCCAGCAACCAGTACGTTCAAGCTAATTCAGTGTTTGAAGCATTTGATGTAC
TaMSH7-3D CS TCCTCAGAGTGCCCCAGCAACCAGTACGTTCAAGCTAATTCAGTGTTTGAAGCATTTGATGTAC
K H R P R I R *
TaMSH7-3A Cadenza AAACACCGTCCCAGGATCCGTTAAAGAGAATCTTTTCTGGGCCTTTTCATGGAGCAGATACA
TaMSH7-3A Paragon AAACACCGTCCCAGGATCCGTTAAAGAGAATCTTTTCTGGGCCTTTTCATGGAGCAGATACA
Q T P S Q D P L K R I F S G P F H G A D T
TaMSH7-3A CS AAACACCGTCCCAGGATCCGTTAAAGAGAATCTTTTCTGGGCCTTTTCATGGAGCAGATACA
TaMSH7-3B CS AAACTCCGTCCCAGGATCCGTTAAAGAGAATCTTTTCTGGGCCTTTTCATGGAGCAGATACA
TaMSH7-3D CS AAACTCCATCCCAGGATCCGTTAAAGAGAATCTTTTCTGGGCCTTTTCATGGAGCAGATACA
c
F1 + R2
100 bp DNA ladder
F2 + R2
F1 + R1
CS Cad N3A H2O
CS Cad N3A H2O
CS Cad N3A H2O
142 bp
114 bp
107 bp
62 bp

Supplementary Figure 9. Identification of a 28 bp deletion within *TaMSH7-3A* that probably leads to non-functional protein in 14 wheat accessions. (a) Gene structure for *TaMSH7-3A* (TraesCS3A01G117500) showing the location of the deletion within exon 2. Exons are shown as red rectangles, 5' and 3' UTR as white rectangles and introns as black lines. **(b)** Alignment of portions of *TaMSH7-3A* exon 2 genomic sequences from Cadenza and Paragon, two English cultivars that contain the deletion, and the corresponding regions in *TaMSH7* homoeologues from Chinese Spring (CS), that do not contain the deletion. The 28 bp deletion is represented by red dashes. Deduced amino acid sequences are shown above nucleotide sequences (in green for Cadenza and Paragon TaMSH7-3A proteins and in pink for Chinese Spring TaMSH7-3A protein). The deletion in Cadenza and Paragon sequences induces a frameshift which leads to a prematurely terminated protein due to a now in-frame STOP codon (*). Locations of primers used for PCR amplifications are shown by coloured arrows (F1 in pink, F2 in orange, R1 in dark blue and R2 in light blue). **(c)** Ethidium bromide stained gels confirming the presence of the deletion within *TaMSH7-3A* gene in Cadenza (Cad) accession. Primers used for PCR amplifications are indicated above the gel. N3A: Chinese Spring nullitetrasomic line for chromosome 3A.

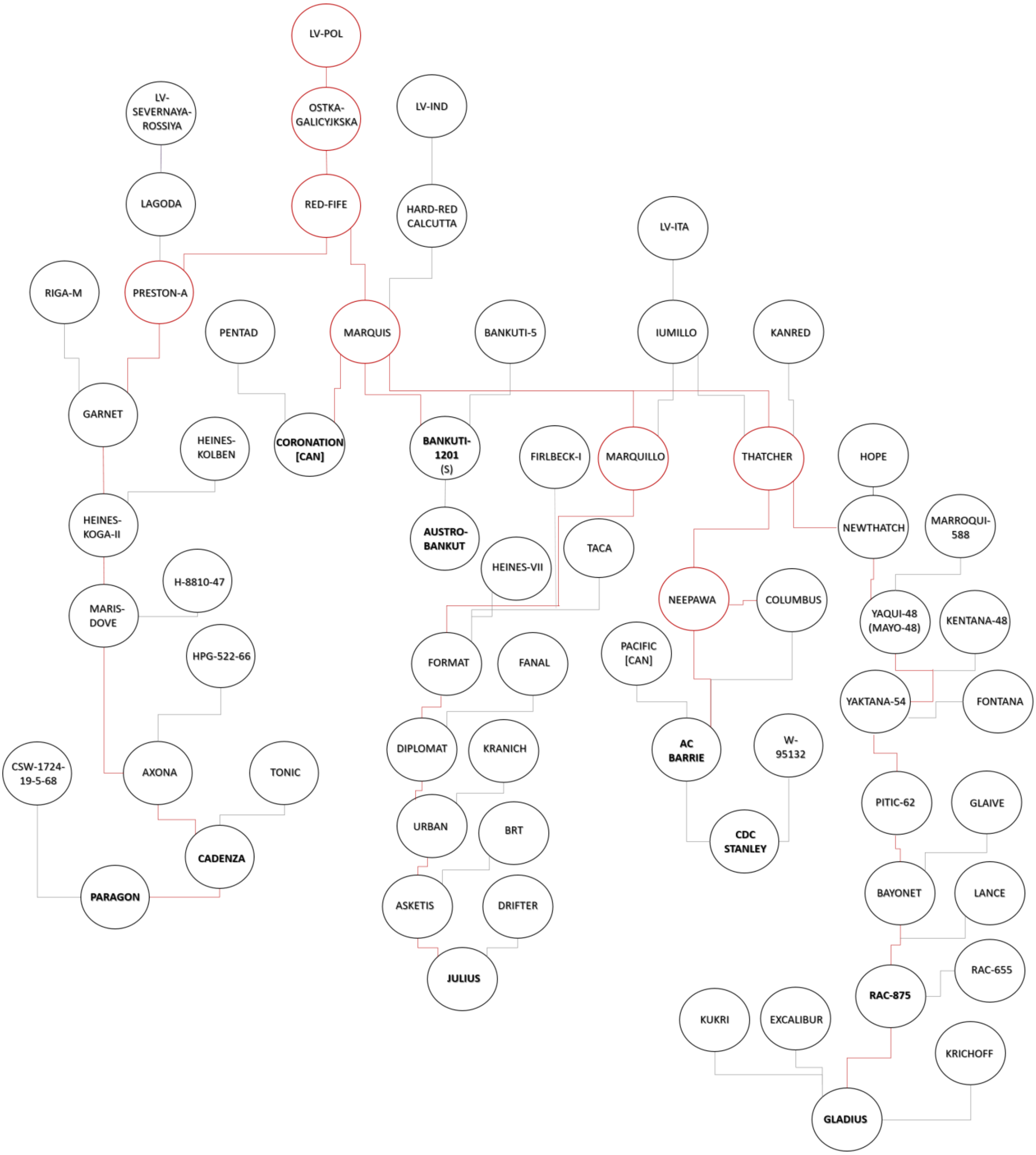

Supplementary Figure 10. **Full or partial pedigrees for several of the wheat accessions that were identified as carrying the 28 bp deletion in *TaMSH7-3A***. Accessions for which the deletion has been ascertained from sequence data are shown in bold font, and accessions that were key for the transmission are encircled in red. The origin of the deletion can be traced back to LV-Pol from which it was transmitted to RED-FIFE. Red connecting lines indicate possible gene flow.

a

	PvMSH7	PhMSH7	SvMSH7	SiMSH7	ZmMSH7	SbMSH7	OtMSH7	OjMSH7	OiMSH7	BdMSH7	BsMSH7	HvMSH7	ScMSH7	TdiMSH7-3B	TaMSH7-3B	TduMSH7-3B	AtaMSH7-3D	TaMSH7-3D	TdiMSH7-3A	TduMSH7-3A	TaMSH7-3A	TuMSH7-3A
PvMSH7		91,3	86,6	86,6	79,1	80,7	73,3	73,2	72,7	75,1	75,3	73,9	74,4	73,9	73,9	73,9	73,7	73,6	74,2	74,2	74,2	73,9
PhMSH7	91,3		88,2	88,2	79,3	81,7	74,4	74,5	74,0	76,3	76,7	74,7	75,6	74,8	74,8	74,7	75,0	74,9	75,1	75,1	75,1	74,8
SvMSH7	86,6	88,2		99,8	77,2	78,9	73,0	72,7	72,3	73,6	73,8	72,7	73,7	73,1	73,1	73,0	73,0	72,9	73,4	73,4	73,4	73,1
SiMSH7	86,6	88,2	99,8		77,3	78,9	73,0	72,6	72,2	73,7	73,9	72,8	73,8	73,1	73,1	73,1	73,1	73,0	73,3	73,3	73,4	73,1
ZmMSH7	79,1	79,3	77,2	77,3		88,7	70,6	70,0	69,5	71,9	71,7	70,0	70,7	70,4	70,4	70,3	70,5	70,5	70,7	70,7	70,7	70,6
SbMSH7	80,7	81,7	78,9	78,9	88,7		71,4	71,9	71,5	73,5	73,5	71,9	72,9	72,6	72,6	72,5	72,6	72,5	72,5	72,5	72,6	72,4
OtMSH7	73,3	74,4	73,0	73,0	70,6	71,4		72,4	72,0	73,6	74,0	71,4	71,8	71,7	71,7	71,6	72,1	72,2	71,7	71,7	71,7	71,4
OjMSH7	73,2	74,5	72,7	72,6	70,0	71,9	72,4		99,1	76,0	76,5	75,8	76,3	76,2	76,2	76,1	76,4	76,4	76,3	76,3	76,3	76,2
OiMSH7	72,7	74,0	72,3	72,2	69,5	71,5	72,0	99,1		75,7	76,1	75,5	75,9	75,8	75,8	75,7	76,1	76,1	75,9	75,9	75,9	75,7
BdMSH7	75,1	76,3	73,6	73,7	71,9	73,5	73,6	76,0	75,7		95,0	83,7	84,7	84,1	84,1	84,0	84,4	84,5	83,7	83,7	83,7	83,5
BsMSH7	75,3	76,7	73,8	73,9	71,7	73,5	74,0	76,5	76,1	95,0		84,1	84,8	84,5	84,5	84,4	84,9	84,9	84,0	84,0	84,0	83,7
HvMSH7	73,9	74,7	72,7	72,8	70,0	71,9	71,4	75,8	75,5	83,7	84,1		94,8	94,0	94,0	94,0	94,4	94,3	93,8	93,8	93,8	93,6
ScMSH7	74,4	75,6	73,7	73,8	70,7	72,9	71,8	76,3	75,9	84,7	84,8	94,8		96,6	96,6	96,6	96,5	96,4	95,8	95,8	95,8	95,6
TdiMSH7-3B	73,9	74,8	73,1	73,1	70,4	72,6	71,7	76,2	75,8	84,1	84,5	94,0	96,6		100	99,9	97,3	97,2	96,3	96,3	96,3	96,2
TaMSH7-3B	73,9	74,8	73,1	73,1	70,4	72,6	71,7	76,2	75,8	84,1	84,5	94,0	96,6	100		99,9	97,3	97,2	96,3	96,3	96,3	96,2
TduMSH7-3B	73,9	74,7	73,0	73,1	70,3	72,5	71,6	76,1	75,7	84,0	84,4	94,0	96,6	99,9	99,9		97,2	97,1	96,2	96,2	96,2	96,1
AtaMSH7-3D	73,7	75,0	73,0	73,1	70,5	72,6	72,1	76,4	76,1	84,4	84,9	94,4	96,5	97,3	97,3	97,2		99,9	96,4	96,4	96,4	96,2
TaMSH7-3D	73,6	74,9	72,9	73,0	70,5	72,5	72,2	76,4	76,1	84,5	84,9	94,3	96,4	97,2	97,2	97,1	99,9		96,3	96,3	96,3	96,2
TdiMSH7-3A	74,2	75,1	73,4	73,3	70,7	72,5	71,7	76,3	75,9	83,7	84,0	93,8	95,8	96,3	96,3	96,2	96,4	96,3		100	99,8	99,7
TduMSH7-3A	74,2	75,1	73,4	73,3	70,7	72,5	71,7	76,3	75,9	83,7	84,0	93,8	95,8	96,3	96,3	96,2	96,4	96,3	100		99,8	99,7
TaMSH7-3A	74,2	75,1	73,4	73,4	70,7	72,6	71,7	76,3	75,9	83,7	84,0	93,8	95,8	96,3	96,3	96,2	96,4	96,3	99,8	99,8		99,7
TuMSH7-3A	73,9	74,8	73,1	73,1	70,6	72,4	71,4	76,2	75,7	83,5	83,7	93,6	95,6	96,2	96,2	96,1	96,2	96,2	99,7	99,7	99,7	

b

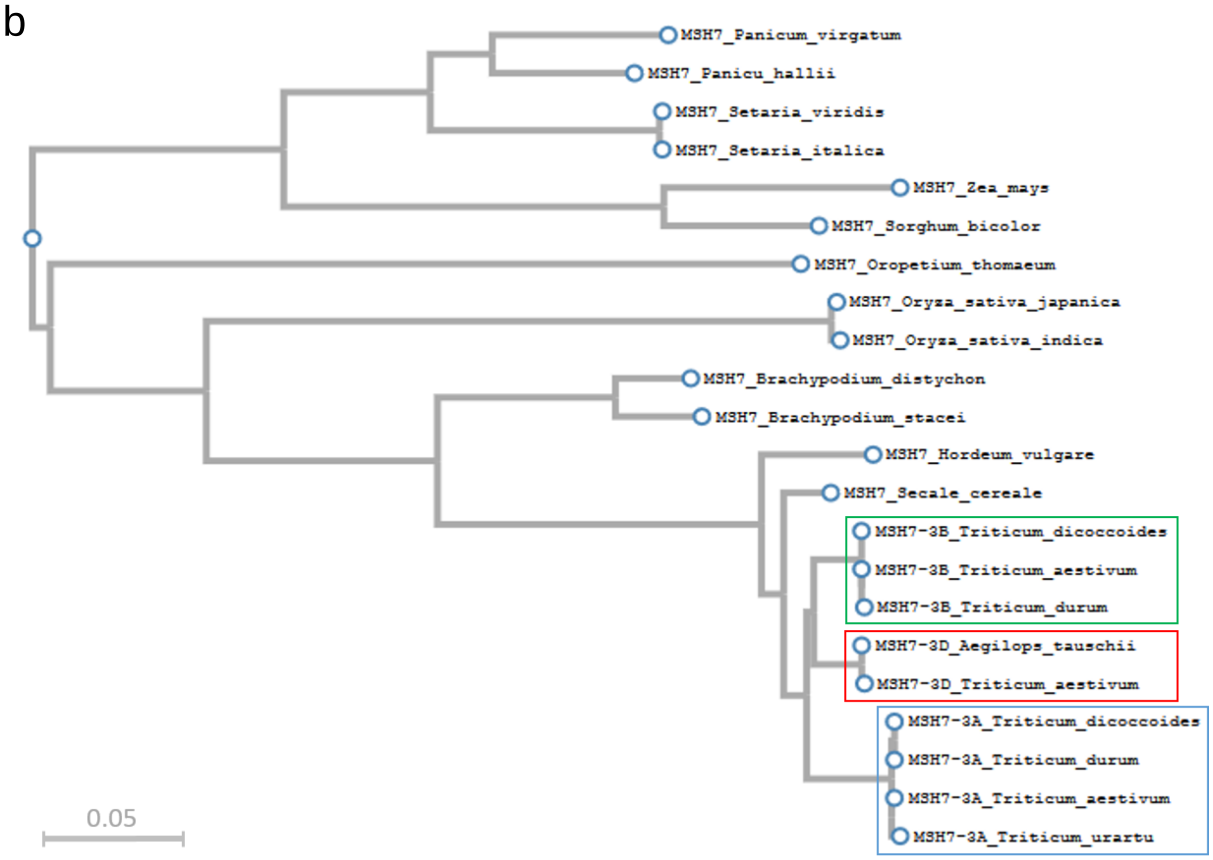

Supplementary Figure 11. Protein sequence identity matrix and phylogenetic tree of MSH7 homologues among grasses. (a) Pairwise percentages of amino acid sequence identities across MSH7 homologues in cereals. *Pv: Panicum virgatum; Ph: Panicum hallii; Sv: Setaria viridis; Si: Setaria italica; Zm: Zea mays; Sb: Sorghum bicolor; Ot: Oropetium thomaeum; Oj: Oryza sativa ssp. Japonica; Oi: Oryza sativa ssp. Indica; Bd: Brachypodium distachyon; Bs: Brachypodium stacei; Hv: Hordeum vulgare* cv. Morex; *Sc: Secale cereale, Tdi: Triticum turgidum ssp. dicoccoides* cv. Zavitan; *Ta: Triticum aestivum* cv. Chinese Spring; *Tdu: Triticum turgidum ssp. durum* cv. Svevo. **(b)** Consensus neighbour-joining tree, generated from 100 bootstrapped trees showing relationships among MSH7 protein sequences in grasses. The scale bar indicates the number of amino acid substitutions per site.

```
OjMSH7         MQPRRRQQQQQSILSFLQKKPAA-------AAGEEGATPERPPRPPA-ASVAGIMERLVR   52
OiMSH7         MQPRRRQQQQQSILSFLQKKPAA-------AAGEEGATPERPPRPPA-ASVAGIMERLVR   52
BdMSH7         MQPRR---QQQSILSFLQPRRSPAGEDALGAGTDTGTPPERPPRPPA-ASVDGIMERLVR   56
BsMSH7         MQPWR---QQQSILSFLQPRRSPAGEDALGTG----TTPERPPRPPA-ASVDGIMERLVR   52
HvMSH7         MQPRR---QQQSILSFLHPRQSPGQE-ALG----AGATPERPPRPPAPSSVDGIMERLVR   52
TuMSH7-3A      MQPRR--QQQQSILPFLHPRQGPAQE-APG----AGRTPERPPRPPAASSVDGIMERLVR   53
TaMSH7-3A      MQPRR--QQQQSILPFLHPRQGPAQE-APG----AGRTPERPPRPPAASSVDGIMERLVR   53
TduMSH7-3A     MQPRR--QQQQSILPFLHPRQGPAQE-APG----AGRTPERPPRPPAASSVDGIMERLVR   53
TdiMSH7-3A     MQPRR--QQQQSILPFLHPRQGPAQE-APG----AGRTPERPPRPPAASSVDGIMERLVR   53
ScMSH7         MQPRR---QQQSILSFLHPRQSPAQE-ALG----AGTPPEKPPRPPAASSVEGIMERLVR   52
TaMSH7-3B      MQPRR---QQQSLLSFLHPRQSPAQE-ALG----AGTPPERPPRPPAASSVDGIMERLVR   52
TdiMSH7-3B     MQPRR---QQQSLLSFLHPRQSPAQE-ALG----AGTPPERPPRPPAASSVDGIMERLVR   52
TduMSH7-3B     MQPRR---QQQSLLSFLHPRQSPAQE-ALG----AGTPPERPPRPPAASSVDGIMERLVR   52
TaMSH7-3D      MQPRR---QQQSIRSFLHPRPSPAQE-ASG----AGTTPERPPRPPAASSVDGIMERLVR   52
AtaMSH7-3D     MQPRR---QQQSIRSFLHPRPSPAQE-ASG----AGTTPERPPRPPAASSVDGIMERLVR   52
OtMSH7         MQP-RRQ-QQQSILSFLQKQPPRRDP----DPAGDGTTPEKPPRPLA-GSISGIMERLVR   53
SbMSH7         MEPRRRQ-QQQSILSFLQKQPPS-WR----VPSGEGTPPEKPTRPPM-GSIAGILERLVR   53
ZmMSH7         MEPRRRQ-QQQSILSFLQKQPPS-WR----DPSGEGTPPEKPPRPPM-GSVAGIMERLVR   53
SiMSH7         MQP-RRQ-QQQSILSFLQKP-PR-DP----AGAGEGTPPEKPPRPPA-GSVAGIMERLVR   51
SvMSH7         MQP-RRQ-QQQSILSFLQKP-PR-DP----AGAGEGTPPEKPPRPPA-GSVAGIMERLVR   51
PhMSH7         MQP-RRQ-QQQSILSFLQKP-PR-DR----AGAGEGTPPEKPPRHPA-GSVAGIMERLVR   51
PvMSH7         MQP-RRQ-QQQSILSFLQKP-PL-DR----AGAGEGTPPEKPPRPPA-GSVASIMERLVR   51
               *:* *   ****:  **:                    **:* *    .*: .*:*****

OjMSH7         PPRQQQQ----GRDQDASQARHVEQRALPVKNQTTSNECSSALFLESCNA----GDNKAA   104
OiMSH7         PQRQQQQ----GRDQDASQARHVEQRALPVKNQTTSNECSSALFLESCNA----GDNKAA   104
BdMSH7         PPSQ-------GRNKDVSQVKHV-EISLPVKSQDPSHECPSALFSGPYKGKYSRETTLET   108
BsMSH7         PPSQ-------GRNKDASQVKHV-EISLPVKSQDPSHECPSALFSGPYNGKYSRETTLET   104
HvMSH7         PPSQ-------GRNKDAAQIRNA-ERALPGKNEDTSNERSSAVFPVPYNGNYSRGT----   100
TuMSH7-3A      PPSQ-------GRNKDAAQIRNA-ERALPGRNEDTSNEQPSASFPVRHNGKYSRGT----   101
TaMSH7-3A      PPSQ-------GRNKDAAQIRNA-ERALPGRNEDTSNEQPSASFPVRHNGKYSRGT----   101
TduMSH7-3A     PPSQ-------GRNKDAAQIRNA-ERALPGRNEDTSNEQPSASFPVRHNGKYSRGT----   101
TdiMSH7-3A     PPSQ-------GRNKDAAQIRNA-ERALPGRNEDTSNEQPSASFPVRHNGKYSRGT----   101
ScMSH7         PPSQ-------GRNKDAAQIRNA-ERALPGKNEDTSNERPSASFPVPYNSKYSRGT----   100
TaMSH7-3B      PPSQ-------GRNKDAAQIRNA-ERALPGKNEDTSNEQPSASFPVRYNGKYSRGT----   100
TdiMSH7-3B     PPSQ-------GRNKDAAQIRNA-ERALPGKNEDTSNEQPSASFPVRYNGKYSRGT----   100
TduMSH7-3B     PPSQ-------GRNKDAAQIRNA-ERALPGKNEDTSNEQPSASFPVRYNGKYSRGT----   100
TaMSH7-3D      PPSQ-------GRNKDATQIRNA-ERALPGKNEDTSNEQPSASFPVRYNGKYSRGT----   100
AtaMSH7-3D     PPSQ-------GRNKDATQIRNA-ERALPGKNEDTSNEQPSASFPVRYNGKYSRGT----   100
OtMSH7         PPQQP------GRIQDAAQVRHVDEKVLPFRNQIPSKGQVSVLSSGLCTGDQNRG-----   102
SbMSH7         PPPPPPQ----ARNQYASQVRHLEGKNLPVENQVLSNECSGALFSRPIYGEDSRT-----   104
ZmMSH7         PPPPPQASHGWARNQYDSQVRHSEGKNLPVENQVLSNECLGALFSRPIYGEHSRT-----   108
SiMSH7         PPPRPQPPQG-SRNQDASQAGHFSGKTLPGRIRVPSDGHSSALSSGSWNGEYGRA-----   105
SvMSH7         PPPRPQPPQG-SRNQDASQAGHFSGKTLPGRIRVPSDGHSSALSSGSWNGEYGRA-----   105
PhMSH7         PPP-PQPSQG-SRNQDASQVGQLDGKTLPGRNQVPSNWRSSALFSGPCNGQYSGA-----   104
PvMSH7         PPP-LS---Q-GRNQDASQVGHLDGKTLTGRNQVPSNGHSTALFSGPCNGEYSGA-----   101
               *          .* :  :*  :     *  . .  *.    .       .

OjMSH7         MLIAEEGSNMTPLQEPLKS-LWPSKDDFVRASTLSPELGLYQNLPPHCPKKLPFESSNNN   163
OiMSH7         MLIAEEGSNMTPLQEPLKS-LWPSKDDFVRASTLSPELGLDQNLPPHCPKKLPFESSNNN   163
BdMSH7         TLFAEHGTNSIPLQEPLKYSMRSSTDEFVRACTLFPEHGSDQT-LQEYPKKFSSESPNNK   167
BsMSH7         KLFAEHGTNIIPLQEPLKSSTRSSADEFVRASTLFPEHGSDQT-LQEYPEKFSSESPNNK   163
HvMSH7         MLFAEHSMDTTPPQEPLKFTARSSTDEFVRASTLFPELGSDQTPLHVCPKKLSSERPNNQ   160
TuMSH7-3A      VLFAEHSTDTTPPQEPLKFSARSSTDEFVRASTLFPELGYP-TLLQECPKKLSSECPSNQ   160
TaMSH7-3A      VLFAEHSTDTTPPQEPLKFSARSSTDEFVRASTLFPELGYP-TLLQECPKKLSSECPSNQ   160
TduMSH7-3A     VLFAEHSTDTTPPQEPLKFSARSSTDEFVRASTLFPELGYP-TLLQECPKKLSSECPSNQ   160
TdiMSH7-3A     VLFAEHSTDTTPPQEPLKFSARSSTDEFVRASTLFPELGYP-TLLQECPKKLSSECPSNQ   160
ScMSH7         MLFAEHSMDTTPPQEPLKFSARSSTDEFVRASTLFPELGSDQTLLQECSKKSSSDCPSNQ   160
TaMSH7-3B      VLFAEHSTDTTPPQEPLKFSARSSTDEFVKASTLFPELGSDQTLLQECPKKLSSECPSNQ   160
TdiMSH7-3B     VLFAEHSTDTTPPQEPLKFSARSSTDEFVKASTLFPELGSDQTLLQECPKKLSSECPSNQ   160
TduMSH7-3B     VLFAEHSTDTTPPQEPLKFSARSSTDEFVKASTLFPELGSDQTLLQECPKKLSSECPSNQ   160
TaMSH7-3D      VLFAEHSTDTTPPQEPLKFSARSSTDEFVRASTLFPEIGSDQTLLQECPKKLSSECPSNQ   160
AtaMSH7-3D     VLFAEHSTDTTPPQEPLKFSARSSTDEFVRASTLFPEIGSDQTLLQECPKKLSSECPSNQ   160
OtMSH7         NEFSEQGAGVSSIYEPPTNSLRSSKDDFVKASILFPEMSSNCTPLKEHPEKISSESPNNR   162
SbMSH7         ILSSEGGGDMAPSQEPQMHSLRSSTDEFIGASTLLPELGSNQAPIQEYPKKLFSESHT-N   163
ZmMSH7         ILSPEGGADMSPSQEPQKHSLRSSTDESTRA---------TKEVILEHPKKLFSESHT-N   158
SiMSH7         TMFPKQGSGIIPSQEPQKYPLRSSTDEFVQASSLVPEFGPNQTPLQAR------------   153
SvMSH7         TMFPKQGSGIIPSQEPQKYPLRSSTDEFVQASSLVPEFGPNQTPLQAR------------   153
PhMSH7         TMFQKHGSGMTPSQEPQMYPLRSSTDEFVQAS-ALPEFGLNQTPRQQHPKKLVSESPNNG   163
PvMSH7         TMFPKQGLGMTPAQEPQKYPLRSSTDEFFQASMLLPEFGLNQSPLQEHPKKLLSESPNNG   161
                   : . .     **       * *:   *
```

```
OjMSH7          CIGANSS--FEEFDVQTPQD-TSKTVFWRSSRGADTPLTESDSDQTPLQHPSKF-SFVSP   219
OiMSH7          CIGANSS--FEEFDVQTPQD-TSKTVFWRSSRGADTPLTESDSDQTPLQHPSKF-SFVSP   219
BdMSH7          YIQATSI--FEEFDVQTPSQNPLKRIFSGPSHGADTPLSEYGSDQTLLQHPSKKLPLVSS   225
BsMSH7          YIQATSI--FEEFDLQTPSQNPLKRIFSGPSHGADTPLSEYGSDQTLLQHPSKKLPLVSS   221
HvMSH7          YAQDNSV--FEAFDVQTPSQEPLKRIFSGPFHGADTPLSEYRSYPIPLQHPSKKLSLGSS   218
TuMSH7-3A       YVQANSV--FEAFDVQTPSQDPLKRIFSGPFHGADTPLSEYRSYPIPLQHPSKKSSSGSS   218
TaMSH7-3A       YVQANSV--FEAFDVQTPSQDPLKRIFSGPFHGADTPLSEYRSYPIPLQHPSKKSSSGSS   218
TduMSH7-3A      YVQANSV--FEAFDVQTPSQDPLKRIFSGPFHGADTPLSEYRSYPIPLQHPSKKSSSGSS   218
TdiMSH7-3A      YVQANSV--FEAFDVQTPSQDPLKRIFSGPFHGADTPLSEYRSYPIPLQHPSKKSSSGSS   218
ScMSH7          YVQANSV--FEAFDVQTPSQDPLKRIFSGPFHAADTPLSEYRSYPIPLQHPLKKLSSGSS   218
TaMSH7-3B       YVQANSV--FEAFDVQTPSQDPLKRIFSGPFHGADTPLSEYRSYPIPLQHPSKNLSSGSS   218
TdiMSH7-3B      YVQANSV--FEAFDVQTPSQDPLKRIFSGPFHGADTPLSEYRSYPIPLQHPSKNLSSGSS   218
TduMSH7-3B      YVQANSV--FEAFDVQTPSQDPLRRIFSGPFHGADTPLSEYRSYPIPLQHPSKNLSSGSS   218
TaMSH7-3D       YVQANSV--FEAFDVQTPSQDPLKRIFSGPFHGADTPLSEYRSYPIPLQHPSKKLSSGSS   218
AtaMSH7-3D      YVQANSV--FEAFDVQTPSQDPLKRIFSGPFHGADTPLSEYRSYPIPLQHPSKKLSSGSS   218
OtMSH7          CIRATSL--FEEFDVQTPQ-NPSKRIFLGPSHDDDTPLSEGGSDQTLLQYP-SKFSLNSA   218
SbMSH7          CIQATAL--NENFDVQTPSQVASKKIFPGLAHGADTPLTGYGSDQTLLQHSSKKFSLVSA   221
ZmMSH7          CIHATAL--NENFGVQTPSQVASKKIFPGLAHGADTPLTGYGSEQTLLQHSSKKFSLVSA   216
SiMSH7          -----SL--FEDFDVQTPSQVSSKKVFLGPAHGADTPLTESGSDRTHLQHSAKKFSLVSA   206
SvMSH7          -----SL--FEDFDVQTPSQVSSKKVFLGPAHGADTPLTESGSDRTHLQHSAKKFSLVSA   206
PhMSH7          CIQATSSFADLDFDIQTPSQFSSKKIFLGPAHGADTPLTEYGSDQTHLQHSAKKFSLVSA   223
PvMSH7          CIQSTSLFED--FGIQTPSQVSSKKIFLGPAHGADTPLTDHGSDRTHLQHLAKKFSLVSA   219
                     :      *.:***.    : :*    :  ****:   *    **:  .     *

OjMSH7          NGEYVRGAT-LFALDSNYTPRRESSEKLSSGPSDLPYIKATKLFTEFDSNGTPSQNHLKK   278
OiMSH7          NGEYVRGAT-LFALDSNYTPRRESSEKLSSGPSDLPYIKATKLFTEFDSNGTPSQNHLKK   278
BdMSH7          SGEYVRAAT-LFGLDSNDTHTPKHAKKLFSRSSEPSYIKGTNLFAEFDSHGTPLQNHSKH   284
BsMSH7          SGEYVRAAT-LFGLDSNDSHTPKHARKLFSRSSEPSYIKGTNLFAEFDSNGTPLQNHSKN   280
HvMSH7          SGEYVRAVT-PLRLDSNDTPTVKHSKKLFSGSSDHSYIKATNLFAEFDSNGTPLQNHSNK   277
TuMSH7-3A       SGEYLRAVT-PLGLDSNDTPIAKHSKKLFSGSSDHSYIKATNLFPEFDSNGTPLQNHLNK   277
TaMSH7-3A       SGEYLRAVT-PLGLDSNDTPIAKHSKKLFSGSSDHSYIKATNLFPEFDSNGTPLQNHLNK   277
TduMSH7-3A      SGEYLRAVT-PLGLDSNDTPIAKHSKKLFSGSSDHSYIKATNLFPEFDSNGTPLQNHLNK   277
TdiMSH7-3A      SGEYLRAVT-PLGLDSNDTPIAKHSKKLFSGSSDHSYIKATNLFPEFDSNGTPLQNHLNK   277
ScMSH7          SGEYLRAVT-PLGLDSNDTPTAKHSKKLFSGSSDHSYIKATNLFPEFDSNGTPLQNHSNK   277
TaMSH7-3B       SGEYLRAVT-PLGLDSNDTPTAKHSKKLFSGSSDHSYIKATNLFPEFDSNGTPLQNHSNK   277
TdiMSH7-3B      SGEYLRAVT-PLGLDSNDTPTAKHSKKLFSGSSDHSYIKATNLFPEFDSNGTPLQNHSNK   277
TduMSH7-3B      SGEYLRAVT-PLGLDSNDTPTAKHSKKLFSGSSDHSYIKATNLFPEFDSNGTPLQNHSNK   277
TaMSH7-3D       SGEYLRAVT-PLGLDSNDTPTAKHSKKLFSGSSDHSYIKATNLFPEFDSNGTPLQNHSNK   277
AtaMSH7-3D      SGEYLRAVT-PLGLDSNDTPTAKHSKKLFSGSSDHSYIKATNLFPEFDSNGTPLQNHSNK   277
OtMSH7          NAGYARTPT-TFALHSNDTPTEQTSKKL-SRSSDLLYIKPTNLFAEIDSNRTPSQNHLTN   276
SbMSH7          NGQYTRAAVTGFGQNSNTTRAEEPSNMLCSGSSDPLYIRPTNLFAELEANETPLKNHSKN   281
ZmMSH7          NGKYTRAAVTPFEQNSNNTRAEELSKM-RSGSSDMLYLKPTNLSAELETNGTPLKNHSKN   275
SiMSH7          NDEYTRAAT-TFVLNSNDTRTEEHLNKLCPGSSDPLYIKATNLFAEFEANATPLKNHSKN   265
SvMSH7          NDEYTRAAT-TFVLNSNDTRTEEHLNKLCPGSSDPLYIKATNLFAEFEANETPLKNHLKN   265
PhMSH7          NGEYTRAAT-TFVLNSNDIHTEESSKKLCPESSVPLYIKATNLFAELEANESPLKNNSKS   282
PvMSH7          NGEYARAAT-TSVLNSNDIHTEEPSKKLCPESSVPLYIKATNLFAELEANESPLKNNSKS   278
                .  * *  .     .**     :  .      *   *:: *:*  *:::: :* :*: .

OjMSH7          LSSVPKNDKRIGAGAELFSEFDPSPLKPETPVMRAVIPRLKRVQEDQRVTTNDSCSPFWG   338
OiMSH7          LSSVPKNDKRIGAGAELFSEFDPSPLKPETPVMRAVIPRLKRVQEDQRVTTNDSCSPFWG   338
BdMSH7          SS-VFMNGKHNGAPATLFPELDSFSLKPEMPMTRAVTPRAKRVQQDQCVTTKNQHPPLWG   343
BsMSH7          SS-VFMNGKHIGAPATLFPELDSFSLKPETPMTRAVTPRAKRVQQDQCVTAKNQHSPLWG   339
HvMSH7          FS-VSMNG--IGAPATLFPELDSVLLKPETPVTRAAAPRGKRVQQDQCMTANNSHSPLWG   334
TuMSH7-3A       FS-VSMNGKHIGAPATLFPELDSVLLKPETPVTRAVAPRGKRVQQDQCMTANNSQSPLWG   336
TaMSH7-3A       FS-VSMNGKHIGAPATLFPELDSVLLKPETPVTRAVAPRGKRVQQDQCMTANNSQSPLWG   336
TduMSH7-3A      FS-VSMNGKHIGAPATLFPELDSVLLKPETPVTRAVAPRGKRVQQDQCMTANNSQSPLWG   336
TdiMSH7-3A      FS-VSMNGKHIGAPATLFPELDSVLLKPETPVTRAVAPRGKRVQQDQCMTANNSQSPLWG   336
ScMSH7          FS-VSMNGKHIGAPATLFPELDSVLLKPETPVTRAVAPRGKRVQQDQCMTANNSQSPLWG   336
TaMSH7-3B       FS-VSMNGKHIGAAATLFPELDSVLLKPETPVTRAVAPRGKRVQQDQRMTANNSQSPLWG   336
TdiMSH7-3B      FS-VSMNGKHIGAAATLFPELDSVLLKPETPVTRAVAPRGKRVQQDQRMTANNSQSPLWG   336
TduMSH7-3B      FS-VSMNGKHIGAAATLFPELDSVLLKPETPVTRAVAPRGKRVQQDQRMTANNSQSPLWG   336
TaMSH7-3D       FS-VSMNSKNIGAPATLFPELDSVLLKPETPVTQAVAPRGKRVQQDQCMTANNCQSPLWG   336
AtaMSH7-3D      FS-VSMNSKNIGAPATLFPELDSVLLKPETPVTQAVAPRGKRVQQDQCMTANNCQSPLWG   336
OtMSH7          FSSVFMNGKPIGAASTTFPDLDSSPLKPKTPAMGATISRFKRVREEQNVTADYQLSP-WQ   335
SbMSH7          -SSLLTNSKYNLASAMLFPGLDSSPLKPETPAMQPVIPRLKRVQEDQSVDANNQCPPLGA   340
ZmMSH7          -SSLLMDGKYNITSAALFPELDSSPLKPETPAMQAAIPRLKRVQEDQGVDANNQCPPLWA   334
SiMSH7          -SSLLMNDKHIGAAATIFPELDSSPLKPETPAMRAVIPRLKRVQEEQGVAANKPCSPLWV   324
SvMSH7          -SSLLMNDKHIGAAATIFPELDSSPLKPETPAMRAVIPRLKRVQEEQGVAANKPCSPLWV   324
PhMSH7          -SSFLMNDKHIGATAALFPDLDSSPLKPETPATQAVIPRLKRVQEEQGVADNKQSFPLWV   341
PvMSH7          -SSFLMSDKHIGATSALFPELDSSPLKPETPAMRAVIPRLKRVQEEQGVADNKQCLPLWV   337
                 * .  ..    : :  *  :*   ***: *    .  * ***:::* :  .    *
```

```
OjMSH7         PNKKVKPAQCSPVENKVHDEMAESARSKFEWLNPSNIRDANRRRLADPLYDKTTLFIPPD   398
OiMSH7         PNKKVKPAQCSPVENKVHDEMAESARSKFEWLNPSNIRDANRRRLADPLYDKTTLFIPPD   398
BdMSH7         SNKKVKSAHCSPAKKMVHDEMAEIARSKFEWLNPLNIRDGNKRRPEDPLYDKRTLFIPPD   403
BsMSH7         SNKKVKSAHCSPVKKMVHDEMAESARSKFEWLNPVNIRDGNKKRPEDPLYDKRTLFIPPD   399
HvMSH7         SNKKVKSAHCSPAGKMVNDEMAESARSKFEWLNPLNIRDANKRRPGDPLYDKRTLFIPPD   394
TuMSH7-3A      SNKKVKSAHCSPAGEMAHDEMAESARSKFEWLNPLNIRDANKRRPDDPLYDKRTLFIPPD   396
TaMSH7-3A      SNKKVKSAHCSPAGKMAHDEMAESARSKFEWLNPLNIRDANKRRPDDPLYDKRTLFIPPD   396
TduMSH7-3A     SNKKVKSAHCSPARKMAHDEMAESARSKFEWLNPLNIRDANKRRPDDPLYDKRTLFIPPD   396
TdiMSH7-3A     SNKKVKSAHCSPARKMAHDEMAESARSKFEWLNPLNIRDANKRRPDDPLYDKRTLFIPPD   396
ScMSH7         SSKKVKSAHCSPAGKMVHDEMAESARSKFEWLNPLNIRDANKRRPDDPLYDKRTLFIPPD   396
TaMSH7-3B      SNKKVKSAHCSPPGKMVHDEMAESARSKFEWLNPLNIRDANKRRPDDPLYDKRTLFIPPD   396
TdiMSH7-3B     SNKKVKSAHCSPPGKMVHDEMAESARSKFEWLNPLNIRDANKRRPDDPLYDKRTLFIPPD   396
TduMSH7-3B     SNKKVKSAHCSPPGKMVHDEMAESARSKFEWLNPLNIRDANKRRPDDPLYDKRTLFIPPD   396
TaMSH7-3D      SNKKVKSAHCSPAGKMVHDEMAESARSKFEWLNPLNIRDANKRRPDDPLYDKRTLFIPPD   396
AtaMSH7-3D     SNKKVKSAHCSPAGKMVHDEMAESARSKFEWLNPLNIRDANKRRPDDPLYDKRTLFIPPD   396
OtMSH7         VLNKMKPAHPHPLEKKIHAEMAESARSKFEWLNPLNIRDANRRCPNDPLYDKSTLFIPPD   395
SbMSH7         LNKKMKSAHCS-IERKDHDEMADSARSKFEWLNPFAIRDANRRRPNDPLYDKSTLFIPPD   399
ZmMSH7         MNKKMKSAHCS-IEKKDHDEMAGSARSKFEWLNSFAIRDANKRRPNDPLYDKSTLFIPPD   393
SiMSH7         SNKKMKSANCSPIEKKDRDEMADSARRKFEWLNPSTIRDANRRRPDDPLYDKSTLFIPPD   384
SvMSH7         SNKKMKSANCSPIEKKDRDEMADSARRKFEWLNPSTIRDANRRRPDDPLYDKSTLFIPPD   384
PhMSH7         SNKKMKSAHCSPVEKKDHDEMTDSARSKFEWLNPSNIRDANRRRPDDPLYDKSTLFIPPD   401
PvMSH7         SNKKMKSAHCSPVEKK--DEMADSARSKFEWLNPSTIRDANRRRPDDPLYDKSTLFIPPD   395
                 :*:* *:     .    **:  ** ******   ***.*::   ****** *******

                         MutS domain I
OjMSH7         ALRKMSTSQKQYWNIKCKYMDVVLFFKVGKFYELYEVDAEIGQKELDWKMTISGVGKCRQ   458
OiMSH7         ALRKMSTSQKQYWNIKCKYMDVVLFFKVGKFYELYEVDAEIGQKELDWKMTISGVGKCRQ   458
BdMSH7         ALRKMSTSQKQYWTTKCKYMDVVLFFKVGKFYELYEVDAEIGQKELDWKMTISGVGKCRQ   463
BsMSH7         ALRKMSKSQKQYWTIKCKYMDVVLFFKVGKFYELYEVDAEIGQKELDWKMTISGVGKCRQ   459
HvMSH7         ALRKMSTSQKQYWTIKCKFMDVLLFFKVGKFYELYEVDAEIGQKELDWKMTISGVGKCRQ   454
TuMSH7-3A      ALRKMSTSQKQYWTIKCKYMDVLLFFKVGKFYELYEVDAEIGQKELDWKMTISGVGKCRQ   456
TaMSH7-3A      ALRKMSTSQKQYWTIKCKYMDVLLFFKVGKFYELYEVDAEIGQKELDWKMTISGVGKCRQ   456
TduMSH7-3A     ALRKMSTSQKQYWTIKCKYMDVLLFFKVGKFYELYEVDAEIGQKELDWKMTISGVGKCRQ   456
TdiMSH7-3A     ALRKMSTSQKQYWTIKCKYMDVLLFFKVGKFYELYEVDAEIGQKELDWKMTISGVGKCRQ   456
ScMSH7         ALRKMSTSQKQYWTIKCKYMDVLLFFKVGKFYELYEVDAEIGQKELDWKMTISGVGKCRQ   456
TaMSH7-3B      ALRKMSTSQKQYWSIKCKYMDVLLFFKVGKFYELYEVDAEIGQKELDWKMTISGVGKCRQ   456
TdiMSH7-3B     ALRKMSTSQKQYWSIKCKYMDVLLFFKVGKFYELYEVDAEIGQKELDWKMTISGVGKCRQ   456
TduMSH7-3B     ALRKMSTSQKQYWSIKCKYMDVLLFFKVGKFYELYEVDAEIGQKELDWKMTISGVGKCRQ   456
TaMSH7-3D      ALRKMSTSQKQYWTIKCKYMDVLLFFKVGKFYELYEVDAEIGQKELDWKMTISGVGKCRQ   456
AtaMSH7-3D     ALRKMSTSQKQYWTIKCKYMDVLLFFKVGKFYELYEVDAEIGQKELDWKMTISGVGKCRQ   456
OtMSH7         ALRKMSTSQKQYWNIKCRYMDVVLFFKVGKFYELYELDAEIGQKELDWKMTVSGVGKCRQ   455
SbMSH7         ALRKMSTSQKQYWNIKCKYMDVVLFFKVGKFYELYELDAEIGQKEFDWKMTANGVGKCRQ   459
ZmMSH7         ALRKMSTSQKQYWNIKCKYMDVVLFFKVGKFYELYELDAEIGQKELDWKMTVSGVGKCRQ   453
SiMSH7         ALRKMSTSQKQYWNIKCKYMDVVLFFKVGKFYELYELDAEIGQKELDWKMTVSGVGKCRQ   444
SvMSH7         ALRKMSTSQKQYWNIKCKYMDVVLFFKVGKFYELYELDAEIGQKELDWKMTVSGVGKCRQ   444
PhMSH7         ALRKMSASQKQYWNIKCKYMDVVLFFKVGKFYELYELDAEVGQKELDWKMTASGVGKCRQ   461
PvMSH7         ALRKMSTSQKQYWNIKCKYMDAVLFFKVGKFYELYEIDAEIGQKELDWKMTVSGVGKCRQ   455
               ****** ******. **::**.:*************:***:****:*****  .*******

                         MutS domain I
OjMSH7         VGISESGIDVAVEKLLARGYKVGRIEQMESADQAKSRGSNSVILRKLVHVSTPSTVGDSN   518
OiMSH7         VGISESGIDVAVEKLLARGYKVGRIEQMESADQAKSRGSNSVILRKLVHVSTPSTVGDSN   518
BdMSH7         VGISESGIDDAVEKLLARGYKVGRIEQMESAVQAKARGPNSRIDRKLVNVSTPSTAADSN   523
BsMSH7         VGISESGIDDAVEKLLARGYKVGRIEQMESAVQAKARGPNSVIDRKLVHVSTPSTAADSN   519
HvMSH7         VGISESGIDNAVEKLLARGYKVGRIEQMESAAQAKVRGPNSVIERKLAHVSTPSTAADSN   514
TuMSH7-3A      VGISESGIDDAVEKLLARGYKVGRIEQMESAAQAKSRGPNSVIERKLAHVSTPSTAADSN   516
TaMSH7-3A      VGISESGIDDAVEKLLARGYKVGRIEQMESAAQAKSRGPNSVIERKLAHVSTPSTAADSN   516
TduMSH7-3A     VGISESGIDDAVEKLLARGYKVGRIEQMESAAQAKSRGPNSVIERKLAHVSTPSTAADSN   516
TdiMSH7-3A     VGISESGIDDAVEKLLARGYKVGRIEQMESAAQAKSRGPNSVIERKLAHVSTPSTAADSN   516
ScMSH7         VGISESGIDDAVEKLLARGYKVGRIEQMESAAQAKARGPNSVIERKLAHVSTPSTAAGSN   516
TaMSH7-3B      VGISESGIDDAVEKLLARGYKVGRIEQMESAAQAKARGPNSVIERKLAHVSTPSTAADSN   516
TdiMSH7-3B     VGISESGIDDAVEKLLARGYKVGRIEQMESAAQAKARGPNSVIERKLAHVSTPSTAADSN   516
TduMSH7-3B     VGISESGIDDAVEKLLARGYKVGRIEQMESAAQAKARGPNSVIERKLAHVSTPSTAADSN   516
TaMSH7-3D      VGISESGIDNAVEKLLARGYKVGRIEQMESAAQAKARGPNSVIERKLAHVSTPSTAADSN   516
AtaMSH7-3D     VGISESGIDNAVEKLLARGYKVGRIEQMESAAQAKARGPNSVIERKLAHVSTPSTAADSN   516
OtMSH7         VGISESGIDNAVDKLVARGYKVGRIEQMESSDQAKARGSNSVIERKLVTVSTPSTAADSN   515
SbMSH7         VGISESGIDDAVDKLVARGYKVGRIEQMESANQAKARGLHSVIERKLVHVSTPSTAAD-N   518
ZmMSH7         VGISESGIDDAVDKLIARGYKVGRIEQMESANQAKARGVHSVIERKLVHVSTPSTAVD-N   512
SiMSH7         VGISESGIDAAADKLVARGYKVGRIEQMESANQAKARGSNAVIERKLLNVSTPSTAVDSN   504
SvMSH7         VGISESGIDAAADKLVARGYKVGRIEQMESANQAKARGSNAVIERKLLNVSTPSTAVDSN   504
PhMSH7         VGISESGIDDAVDKLVARGYKVGRIEQMESANQAKARGSNSVIERKLLHVSTPSTAVDSN   521
PvMSH7         VGISESGIDDAVDKLVARGYKVGRIEQMESANQAKARGSNSVIERKLLHVSTPSTAVDGN   515
               ********* *.:**:**************: *** ** :: * ***  ******. . *
```

MutS domain II

```
OjMSH7        IGADAVHLLSLKEITLASNGSRVYGFAFLDYAALKIWVGSVHDDDTFAALGALLVQVSPK   578
OiMSH7        IGADAVHLLSLKEITLASNGSRVYGFAFLDYAALKIWVGSVHDDDTFAALGALLVQVSPK   578
BdMSH7        IGADAVHLLALKEVTLASNGSRVYGFAFLDYAALKIWVGSLHDDDSSAALGALLVQVSPR   583
BsMSH7        IGADAVHLLALKEVTLASNGSRVYGFAFLDYAALKIWVGSLHDDDSSAALGALLVQVSPR   579
HvMSH7        IGPDAVHLLALKEVTLASNGSRVYGFAFLDYAALKIWVGSLQDDDSSAALGALLVQVAPR   574
TuMSH7-3A     IGPDAVHLLALKEVTLASNGSRVYGFAFLDYAALKIWVGSLQDDDSSAALGALLVQVSPR   576
TaMSH7-3A     IGPDAVHLLALKEVTLASNGSRVYGFAFLDYAALKIWVGSLQDDDSSAALGALLVQVSPR   576
TduMSH7-3A    IGPDAVHLLALKEVTLASNGSRVYGFAFLDYAALKIWVGSLQDDDSSAALGALLVQVSPR   576
TdiMSH7-3A    IGPDAVHLLALKEVTLASNGSRVYGFAFLDYAALKIWVGSLQDDDSSAALGALLVQVSPR   576
ScMSH7        IGPDAVHLLALKEVTLASSGSRVYGFAFLDYAALKIWVGSLQDDDSSAALGALLVQVSPR   576
TaMSH7-3B     IGPDAVHLLALKEVTLASNGSRLYGFAFLDYAALKIWVGSLQDDDSSAALGALLVQVSPR   576
TdiMSH7-3B    IGPDAVHLLALKEVTLASNGSRLYGFAFLDYAALKIWVGSLQDDDSSAALGALLVQVSPR   576
TduMSH7-3B    IGPDAVHLLALKEVTLASNGSRLYGFAFLDYAALKIWVGSLQDDDSSAALGALLVQVSPR   576
TaMSH7-3D     IGPDAVHLLALKEVTLASNGSRVYGFAFLDYAALKIWVGSLQDDDSSAALGALLVQVSPR   576
AtaMSH7-3D    IGPDAVHLLALKEVTLASNGSRVYGFAFLDYAALKIWVGSLQDDDSSAALGALLVQVSPR   576
OtMSH7        IGPDAVHLLALKELTVASNGCRVFGFAFLDYAALKIWMGSFHDDDLLAALGALLVQVSPR   575
SbMSH7        IGTDAVHLLALKEVTLASSSSQVYGFAFLDYAALKIWVGSVQDDDSSAALGALLMQVSPR   578
ZmMSH7        IGTDAVHLLALKEVTLASSGFQVFGFAFLDYAALKIWVGSVQDDDSSAALGALLMQVSPR   572
SiMSH7        IGTDAVHLLALKEVTLSSSSSRVYGFAFLDYAALKIWVGSLHDDDSSAALGALLVQVSPR   564
SvMSH7        IGTDAVHLLALKEVTLSSSSSRVYGFAFLDYAALKIWVGSLHDDDSSAALGALLVQVSPR   564
PhMSH7        IGTDAVHLLALKEVTLSSSGSRVYGFAFLDYAALKIWVGSLHDDDSSAALGALLVQVSPR   581
PvMSH7        IGTDAVHLLALKEVTLSSSGSRVYGFTFLDYAALKIWVGSLHDDDSSAALGALLVQVSPR   575
              ** ******:***:*::*.. :::**:**********:**.:***  *******:**:*:
```

MutS domain II

```
OjMSH7        EIIYETSGLSKETHRLIKKYASAGSVKMQLTPLYGLYFSDVSEIQTLIDSRGYFKASTSS   638
OiMSH7        EIIYETSGLSKETHRLIKKYASAGSVKMQLTPLYGLYFSDVSEIQTLIDSRGYFKASTSS   638
BdMSH7        EIIYESSGLSRESHKCMTKYASAGSVKMQLTPLSRTDFSDSSQIRMSVHSKGYFKASTDS   643
BsMSH7        EIIYESSGLSRESRKCMTKYASAGSVKMQLTPLSGTDFSGSSQIRMSVHSKGYFKASTDS   639
HvMSH7        EIIHEFSGLSRESRKSMIKYASAGSVKMQLTPLPGTDFSDASQIQMLVHSKGYFKASTDS   634
TuMSH7-3A     EIIYESSGLSRESRKSMIKYASAGSVKMQLTPLPGTGFSDASQIQMLVHSKGYFKASTDS   636
TaMSH7-3A     EIIYESSGLSRESRKSMIKYASAGSVKMQLTPLPGTGFSDASQIQMLVHSKGYFKASTDS   636
TduMSH7-3A    EIIYESSGLSRESRKSMIKYASAGSVKMQLTPLPGTGFSDASQIQMLVHSKGYFKASTDS   636
TdiMSH7-3A    EIIYESSGLSRESRKSMIKYASAGSVKMQLTPLPGTGFSDASQIQMLVHSKGYFKASTDS   636
ScMSH7        EIIYESSGLSRESRKSVSKYASAGSVKMQLTPLPGTDFSDASQIQMLVHSKGYFKASTDS   636
TaMSH7-3B     EIIYESSGLSRESRKSMIKYASAGSVKMQLTPLPGTDFSDASQIQMLAHSKGYFKASTDS   636
TdiMSH7-3B    EIIYESSGLSRESRKSMIKYASAGSVKMQLTPLPGTDFSDASQIQMLAHSKGYFKASTDS   636
TduMSH7-3B    EIIYESSGLSRESRKSMIKYASAGSVKMQLTPLPGTDFSDASQIQMLAHSKGYFKASTDS   636
TaMSH7-3D     EIIYESSGLSRESRKSMIKYASAGSVKMQLTPLPGTDFSDASQIQMLVHSKGYFKASTDS   636
AtaMSH7-3D    EIIYESSGLSRESRKSMIKYASAGSVKMQLTPLPGTDFSDASQIQMLVHSKGYFKASTDS   636
OtMSH7        EVIYETSGLSKETHTSMRKYASAGSVKMQLTPLSTIDFSDAAQIRMLVHSKGYFKASKDS   635
SbMSH7        ELIYETSGITKETQRTIRKYASAGSVKMQLTPLSGIDFSDASQIRMLIHSKGFFNASTES   638
ZmMSH7        ELIYETSGISKETQRTIRKYASAGSVKMQLTPLSGIDFSDAAQIRNLIHSKGFFNASTES   632
SiMSH7        EIIYETSGLSKETHKAIRKYASAGSVKMQLTPLPGIDFSDVSQIRMLIHSKEYFTASAES   624
SvMSH7        EIIYETSGLSKETHKAIRKYASAGSVKMQLTPLPGIDFSDVSQIRMLIHSKEYFTASAES   624
PhMSH7        EIIYETSGLSKETHKAIRKYASAGSVKMQLTPLPGIDFSDISQIQMLIHSKEYFTASAES   641
PvMSH7        EIIYETSGLSKETHKAIRKYASAGSVKMQLTPLPGIDFSDVSQIRMLIHSNEYFTASAES   635
              *:*:* **:::*::  : ***************    **. ::*:   .*. :*.** .*
```

MutS domain II

```
OjMSH7        WLSALNSSVNKDAVICALGGLVSHLTRLMLEDALKNGEVLAYHVYRTCLRMDGQTLVNLE   698
OiMSH7        WLSALNSSVNKDAVICALGGL--------LEDALKNGEVLAYHVYRTCLRMDGQTLVNLE   690
BdMSH7        WLSALDYSMNQDAVICALGGLIGHLTRLMLDDALKNGEVLPYKVYQTCLRMDGQTLVNLE   703
BsMSH7        WLSALDYTMNQDAVICALGGLIGHLTRLMLDDALKNGEVLPYKVYQTCLRMDGQTLVNLE   699
HvMSH7        WSSALDYLENRDAVICALGGLIGHLTRLMLDDALKNGEVLPYNVYQTCLRMDGQTLVNLE   694
TuMSH7-3A     WLSALDYSVNRDAVIFALGGLIGHLTRLMLDDALKNGEVLPYNVYQTCLRMDGQTLVNLE   696
TaMSH7-3A     WLSALDYSVNRDAVIFALGGLIGHLTRLMLDDALKNGEVLPYNVYQTCLRMDGQTLVNLE   696
TduMSH7-3A    WLSALDYSVNRDAVIFALGGLIGHLTRLMLDDALKNGEVLPYNVYQTCLRMDGQTLVNLE   696
TdiMSH7-3A    WLSALDYSVNRDAVIFALGGLIGHLTRLMLDDALKNGEVLPYNVYQTCLRMDGQTLVNLE   696
ScMSH7        WLSALDYSVNRDAVICALGGLIGHLTRLMLDDALKNGEVLPYNVYQTCLRMDGQTLVNLE   696
TaMSH7-3B     WLSALDYSVNRDAVICALGGLIGHLTRLMLDDALKNGEVLPYNVYQTCLRMDGQTLVNLE   696
TdiMSH7-3B    WLSALDYSVNRDAVICALGGLIGHLTRLMLDDALKNGEVLPYNVYQTCLRMDGQTLVNLE   696
TduMSH7-3B    WLSALDYSVNRDAVICALGGLIGHLTRLMLDDALKNGEVLPYNVYQTCLRMDGQTLVNLE   696
TaMSH7-3D     WLSALDYSVNQDAVICALGGLIGHLTRLMLDDALKNGEVLPYNVYQTCLRMDGQTLVNLE   696
AtaMSH7-3D    WLSALDYSVNRDAVICALGGLIGHLTRLMLDDALKNGEVLPYNVYQTCLRMDGQTLVNLE   696
OtMSH7        WLSALDASVNQDAAVCALGGLIGHVTRLMLDDALTNGEILPYHVYSTSLRMDGQTLVNLE   695
SbMSH7        WLSALDCAVNRDVVICALGGLIGHLTRLMLHDALKNGEVLPYHVYKTCLRMDGQTLVNLE   698
ZmMSH7        WLSALDCTMNQDVVICALGGLIGHLTRLMLHDALKNGEVLPYHVYKTCLRMDGQTLVNLE   692
SiMSH7        WLSALDCALNRDAIICALGGLIGHLTRLMLHDALKNGEVLSYHVYKTCLRMDGQTLVNLE   684
SvMSH7        WLSALDCALNRDAIICALGGLIGHLTRLMLHDALKNGEVLSYHVYKTCLRMDGQTLVNLE   684
PhMSH7        WLSALDCTLNRDAVICALGGLIGHLTRLMLHDALKNGEVLPYHVYKTCLRMDGQTLVNLE   701
PvMSH7        WSSALDCVFNRDAVICALGGLIGHLTRLMLHDALKNGEVLPYHVYKTCLRMDGQTLVNLE   695
              * ***:   *:*. : *****        *.***.***:* *:** *.************
```

MutS domain III

```
OjMSH7       IFSNNFDGGSSGTLYKHLNHCITPCGKRLLRRWICHPLKDIDAINERLDIVEGFIQNCGL  758
OiMSH7       IFSNNFDGGSSGTLYKHLNHCITPCGKRLLRRWICHPLKDIDAINERLDIVEGFIQNCGL  750
BdMSH7       IFSNNFDGGSSGTLYKHLNHCITASGKRLLRRWICHPLKDVDAINSRLDIVEGFIQNCGV  763
BsMSH7       IFSNNFDGGSSGTLYKHLNHCITASGKRLLRRWICHPLKDVDAITSRLDIVEGFIQNCGV  759
HvMSH7       IFSNNFDGGSSGTLYKHLNHCITASGKRLLRRWICHPLKDVDAINRRLDIVEGFIQHCGV  754
TuMSH7-3A    IFGNNFDGGSSGTLYKHLNHCITASGKRLLRRWICHPLKDVDAINRRLDVVEGFIQHCGV  756
TaMSH7-3A    IFGNNFDGGSSGTLYKHLNHCITASGKRLLRRWICHPLKDVDAINRRLDVVEGFIQHCGV  756
TduMSH7-3A   IFGNNFDGGSSGTLYKHLNHCITASGKRLLRRWICHPLKDVDAINRRLDVVEGFIQHCGV  756
TdiMSH7-3A   IFGNNFDGGSSGTLYKHLNHCITASGKRLLRRWICHPLKDVDAINRRLDVVEGFIQHCGV  756
ScMSH7       IFSNNFDGGSSGTLYKHLNHCITASGKRLLRRWICHPLKDVDAINRRLDIVEGFIQHCGV  756
TaMSH7-3B    IFGNNFDGGSSGTLYKHLNHCITASGKRLLRRWICHPLKDVDAINRRLDIVEGFIQHCGV  756
TdiMSH7-3B   IFGNNFDGGSSGTLYKHLNHCITASGKRLLRRWICHPLKDVDAINRRLDIVEGFIQHCGV  756
TduMSH7-3B   IFGNNFDGGSSGTLYKHLNHCITASGKRLLRRWICHPLKDVDAINRRLDIVEGFIQHCGV  756
TaMSH7-3D    IFGNNFDGGSSGTLYKHLNHCITASGKRLLRRWICHPLKDVDAINRRLDIVEGFIQHCGV  756
AtaMSH7-3D   IFGNNFDGGSSGTLYKHLNHCITASGKRLLRRWICHPLKDVDAINRRLDIVEGFIQHCGV  756
OtMSH7       IFRNSFDSGPSGTLYKHLNHCITAFGKRLLRSWICHPLKDVDAINRRLDIVEGFTQNSGL  755
SbMSH7       IFSNNFNGGSSGTLYKHLNHCVTASGKRLLRRWICHPLKDIDAINKRLDVVEGFIQNCGL  758
ZmMSH7       IFSNNFNGGSSGTLYKHLNHCVTASGKRMLRRWICHPLKDIDAINKRLDVVEGFIQNCGL  752
SiMSH7       IFSNNFDGGSSGTLYKHLNQCVTASGKRLLRRWICHPLKDIDAINKRLDVVEAFIQNCGL  744
SvMSH7       IFSNNFDGGSSGTLYKHLNQCVTASGKRLLRRWICHPLKDIDAINKRLDVVEAFIQNCGL  744
PhMSH7       IFSNNFDGGSSGTLYKHLNHCVTASGKRLLRRWICHPLKDIDAINKRLDVVEAFIQNCGL  761
PvMSH7       IFSNNFDGGSSGTLYKHLNHCVTSSGKRLLRRWICHPLKDIDAINERLDVVEAFIQNCGL  755
             ** *.*:.* *********:*:*  ***:** ********:***. ***:**.* *:.*:
```

MutS domain III

```
OjMSH7       GSVTLEHLRKVPDLERLLGRVKSTVGLSSAVLLPFVGEKILKRRIKTFGMLVKGLRVGID  818
OiMSH7       GSVTLEHLRKVPDLERLLGRVKSTVGLSSAVLLPFVGEKILKRRIKTFGMLVKGLRVGID  810
BdMSH7       GSITLEYLRKIPDLERLLGRVRSTVGLTSDVMLPFVGERMLKRRIKTFGMLIKGLRVGID  823
BsMSH7       GSITLEHLRKIPDLERLLGRVRSTVGSTSDVMLPFVGERILKRRIKTFGKLIKGLGVGID  819
HvMSH7       GSITLEHLRKIPDLERLLGRVRSTIGLTSAVLLPFVGDKILKRRIKTFGMLIKGLRVGID  814
TuMSH7-3A    GSITLYYLRKIPDLERLLGRIRSTVGLTSAVLLPFVGEKILKRRIKTFGMLIKGLRVGID  816
TaMSH7-3A    GSITLYYLRKIPDLERLLGRIRSTVGLTSAVLLPFVGEKILKRRIKTFGMLIKGLRVGID  816
TduMSH7-3A   GSITLYYLRKIPDLERLLGRIRSTVGLTSAVLLPFVGEKILKRRIKTFGMLIKGLRVGID  816
TdiMSH7-3A   GSITLYYLRKIPDLERLLGRIRSTVGLTSAVLLPFVGEKILKRRIKTFGMLIKGLRVGID  816
ScMSH7       GSIVLEHLRKIPDLERLLGRVRSTVGLTSAVLLPFVGEKILKRRIKTFGMLIKGLRVGID  816
TaMSH7-3B    GSITLEHLRKIPDLERLLGRVRSTVGLTSAVLLPFVGEKILKRRIKTFGMLIKGLRVAID  816
TdiMSH7-3B   GSITLEHLRKIPDLERLLGRVRSTVGLTSAVLLPFVGEKILKRRIKTFGMLIKGLRVAID  816
TduMSH7-3B   GSITLEHLRKIPDLERLLGRVRSTVGLTSAVLLPFVGEKILKRRIKTFGMLIKGLRVAID  816
TaMSH7-3D    GSVTLEHLRKIPDLERLLGRVRSTVGLTSAVLLPFVGEKILKRRIKTFCMLIKGLRVAID  816
AtaMSH7-3D   GSVTLEHLRKIPDLERLLGRVRSTVGLTSAVLLPFVGEKILKRRIKTFCMLIKGLRVAID  816
OtMSH7       SSFTLESLRKIPDLERLLGRVRSTVGLSSTARLPFVGEKILKRRIKTFCTLIKGLKVGIK  815
SbMSH7       GPTTLGYLRKIPDLERLLGQVRSTVGLSSLLQLPFIGEKILKRRIKTFTMLINGLRNGID  818
ZmMSH7       GPTTLGYLQKIPDLERLLGQVRSTVGLSSLLQLPFIGEKIIKKRIKTFIMLINGLRNGID  812
SiMSH7       GPTTLGYLRKIPDLERLLGQVKSTVGLSSSIQLPFVGERILKKRIRTFIMLINGLRNGLD  804
SvMSH7       GPTTLGYLRKIPDLERLLGQVKSTVGLSSSIQLPFVGERILKKRIRTFIMLINGLRNGLD  804
PhMSH7       GPTTLVYLRKIPDLERLLGQVRSTVGLPSSVKLPFVGERILKKRIKTFIMLINGLRNGLD  821
PvMSH7       GPITRGYLHKIPDLERLLGQVRSTVGLSSSVTLPFVGERILKKRIKTFIMLINGLRNGLD  815
             .  .    *:*:********:::**:*  *   ***:*::::*:**:**  *::**  .:.
```

MutS domain III

```
OjMSH7       LLDILQRQDHGISALSKAVDIPTLSSLGELIHHFEEAIDDDFPR-YQDHSVKDDDANTLA  877
OiMSH7       LLDILQRQDHGISALSKAVDIPTLSSLGELIHHFEEAIDDDFPR-YQDHSVKDDDANTLA  869
BdMSH7       LLSILQREDHGISALSKSVDIPTLSSLGELIHQFEEAIDNDFPR-YQDHDIKDDDANTLA  882
BsMSH7       LLSILQREDHGISALSKSVDIPTLSSLGELIHQFEEAIDNDFPR-YQDHDIKDDDANTLA  878
HvMSH7       LLSALRKDDHGIPALSKSVDIPTLSSLDELVHQFEEAIRNDFEQ-FQDHDIKDDDAITLA  873
TuMSH7-3A    LLSALRRDDHGIPALSKSVDIPTLSSLDELVHQFEEDIRNDFEQ-YQDHDIKDGDATTFA  875
TaMSH7-3A    LLSALRRDDHGIPALSKSVDIPTLSSLDELVHQFEEDIHNDFEQ-YQDHDIKDGDATTLA  875
TduMSH7-3A   LLSALRRDDHGIPALSKSVDIPTLSSLDELVHQFEEDIRNDFEQ-YQDHDIKDGDATTLA  875
TdiMSH7-3A   LLSALRRDDHGIPALSKSVDIPTLSSLDELVHQFEEDIRNDFEQ-YQDHDIKDGDATTLA  875
ScMSH7       LLSALRREDHGIPALSKSVDIPTLSSLDELVHQFEEAIRNDFEQ-YQDHDIKDDDATTLA  875
TaMSH7-3B    LLSALHREDHGIPALSKSVDIPTLSSLDELVHQFEEDIRIDFEQ-YQDHDIKDNDATILA  875
TdiMSH7-3B   LLSALHREDHGIPALSKSVDIPTLSSLDELVHQFEEDIRIDFEQ-YQDHDIKDNDATILA  875
TduMSH7-3B   LLSALHREDHGIPALSKSVDIPTLSSLDELVHQFEEDIRIDFEQ-YQDHDIKDNDATILA  875
TaMSH7-3D    LLSALRREDHGIPALSKSVDIPTLSSLDESVHQFEEAIRIDFEQ-YQDHDIKDHDATTLA  875
AtaMSH7-3D   LLSALRREDHGIPALSKSVDIPTLSSLDESVHQFEEAIRIDFEQ-YQDHDIKDHDATTLA  875
OtMSH7       LLNDLQRGDYGVSALSKVVEIPTLSSLDELINNFDDAIDKEFPKD-QDCSVKEDDHNTLI  874
SbMSH7       LLSDLQRADHGVLALYKIVEIPSLNCLCELIHTFEERIQNDFPCG-QDPDVDNNGANNLV  877
ZmMSH7       LLNDLQRADHGILALYKIVDIPSLSYLPELIHKFEERMQNEFPCG-QVSDVNANGANDLA  871
SiMSH7       LLNDLQRADHGVSALYKVVEIPTLSSLHELIHQFEKRVQEEFPC-YQDLGVEDSDGDTLA  863
SvMSH7       LLNDLQRADHGVSALYKVVEIPTLSSLHELIHQFEKRVQEEFPC-YQDLGVEDSDGDTLA  863
PhMSH7       LLNDLQRADHGVSALYKAVEIPALSSLRELIHQFEENVQNEFP-YYQDLDVKDDDGNTLA  880
PvMSH7       LLNDLQRADHGASALYKAVEIPTLSSLHELIHKFEEEVQNQFPHYYQDNDVKDGDRNKLA  875
             **. *:: *:*  ** * *:**:*. * * :: *:. :  :*     *  .:.  .   :
```

MutS domain III

```
OjMSH7          MLVDLLVGKASEWSLVINALSTIDVLRSFAAMALSSFGTTCRPNILLKGKAPVLQMKGLW   937
OiMSH7          MLVDLLVGKASEWSLVINALSTIDVLRSFAAMALSSFGTTCRPNILLKGKAPVLQMKGLW   929
BdMSH7          ILVELFVGKASEWSFVINAISNIDVLRSFGAMALSSFGTMCRPQILLKDKVPILRMKGLW   942
BsMSH7          LLVELFVGKASEWSLVINVISNIDVLRSFGAMALSSFGAVCRPKILLKDKVPILRMKGLW   938
HvMSH7          ILVELFVGKASEWSLVINAISTVDVLRSFASMALSSFGTMCRPRILLKDKSPILRMKGLW   933
TuMSH7-3A       NLVEHFVGKATEWSLVINAISTVDVLRSFAAMALSSFGTMCRPCILLKDKSPILRMKGLW   935
TaMSH7-3A       NLVEHFVGKATEWSLVINAISTVDVLRSFAAMALSSFGTMCRPCILLKDKSPILRMKGLW   935
TduMSH7-3A      NLVEHFVGKATEWSLVINAISTVDVLRSFAAMALSSFGTMCRPCILLKDKSPILRMKGLW   935
TdiMSH7-3A      NLVEHFVGKATEWSLVINAISTVDVLRSFAAMALSSFGTMCRPCILLKDKSPILRMKGLW   935
ScMSH7          ILVELFVGKASEWSLVINAISTVDVLRSFAAMALSSFGTMCRPRILLKDKSPILRMKGLW   935
TaMSH7-3B       NSVELFVGKATEWSLVINAISTVDVLRSFAAMALSSFGTMCRPRILLKDKSPILRMKGLW   935
TdiMSH7-3B      NSVELFVGKATEWSLVINAISTVDVLRSFAAMALSSFGTMCRPRILLKDKSPILRMKGLW   935
TduMSH7-3B      NSVELFVGKATEWSLVINAISTVDVLRSFAAMALSSFGTMCRPRILLKDKSPILRMKGLW   935
TaMSH7-3D       NLVEHFVGKATEWSLVINAISTVDVLRSFAAMALSSFGTMCRPRILLKDKSPILRMKGLW   935
AtaMSH7-3D      NLVEHFVGKATEWSLVINAISTVDVLRSFAAMALSSFGTMCRPRILLKDKSPILRMKGLW   935
OtMSH7          VLVELFVGKSSEWSLVINALSTVDVLRSFAAVEVSSFGTMCRPHVLVKDNKPILRMKGLW   934
SbMSH7          RLVELFIGKASEWSLVINAVSTIDVLRSFAAMTLSSFGAMCRPQVLLKDDVPILRMKGLW   937
ZmMSH7          ALMDVFIGKASEWSLVINAVSTIDVLRSFAAMTLSSFGAMCRPQVLLKDDVPVLRMKGLW   931
SiMSH7          LLVGLFVRKASEWSLVINAVSTIDVLRSFAAMTLSSFGTMCKPHILLKDDVPILRMKGLW   923
SvMSH7          LLVGLFVRKASEWSLVINAVSTIDVLRSFAAMTLSSFGTMCKPHILLKDDVPILRMKGLW   923
PhMSH7          VLVELFIGKASEWSLVINAVSTIDVLRSFAATTLSSFGAMCRPHILLKDDVPILRMKGLW   940
PvMSH7          VLVEDFVGKASEWSLVINAVSTIDVLRSFAEMTLSSFGAMCRPHVILKDDVPILRMKGLW   935
                  :  :: *::***:***.:*.:******.   :****: *:* :::*.. *:*:*****
```

MutS domain V

```
OjMSH7          HPYAFAESVNGLVPNDLSLGQNLSGQNRFALLLTGPNMGGKSTIMRATCLAIVLAQLGCY   997
OiMSH7          HPYAFAESVNGLVPNDLSLGQDLSGQNRFALLLTGPNMGGKSTIMRATCLAIVLAQLGCY   989
BdMSH7          HPYAFAESTTGLVPNDLSLGQDLSGDNRFALLLTGPNMGGKSTIMRATCLAVVLAQLGCY   1002
BsMSH7          HPYAFAESTTGLVPNDLSLGQDISGDNRFALLLTGPNMGGKSTIMRATCLAVVLAQLGCY   998
HvMSH7          HPYAFAESGTGLVPNDLSLGQDLSGHNRFALLLTGPNMGGKSTIMRATCLAIVLAQLGCY   993
TuMSH7-3A       HPYAFAESGTGLVPNDLSLGQDLSGHNRFALLLTGPNMGGKSTIMRATCLAIVLAQLGCY   995
TaMSH7-3A       HPYAFAESGTGLVPNDLSLGQDLSGHNRFALLLTGPNMGGKSTIMRATCLAIVLAQLGCY   995
TduMSH7-3A      HPYAFAESGTGLVPNDLSLGQDLSGHNRFALLLTGPNMGGKSTIMRATCLAIVLAQLGCY   995
TdiMSH7-3A      HPYAFAESGTGLVPNDLSLGQDLSGHNRFALLLTGPNMGGKSTIMRATCLAIVLAQLGCY   995
ScMSH7          HPYAFAESGTGLVPNDLSLGQDLLGHNRFALLLTGPNMGGKSTIMRATCLAIVLAQLGCY   995
TaMSH7-3B       HPYAFAESGTGLVPNDLSLGQDLLGHNRFALLLTGPNMGGKSTIMRATCLAIVLAQLGCY   995
TdiMSH7-3B      HPYAFAESGTGLVPNDLSLGQDLLGHNRFALLLTGPNMGGKSTIMRATCLAIVLAQLGCY   995
TduMSH7-3B      HPYAFAESGTGLVPNDLSLGQDLLGHNRFALLLTGPNMGGKSTIMRATCLAIVLAQLGCY   995
TaMSH7-3D       HPYAFAESGTGLVPNDLSLGQDLLGHNRFALLLTGPNMGGKSTIMRATCLAIVLAQLGCY   995
AtaMSH7-3D      HPYAFAESGTGLVPNDLSLGQDLLGHNRFALLLTGPNMGGKSTIMRATCLAIVLAQLGCY   995
OtMSH7          HPYAFAESTNGLVPNDLSIGQDLSGLNRFALLLTGPNMGGKSTIMRATCLAVILAQLGCF   994
SbMSH7          HPYAFAENANGLVPNDLTLGQDLSGLNRFALLLTGPNMGGKSTIMRATCLAVVLAQLGCY   997
ZmMSH7          HPYAFAGNANSLVPNDLTLGQDLSGLNRFALLLTGPNMGGKSTIMRATCLAVVLAQLGCY   991
SiMSH7          HPYAFAESANGLVPNDLTLGQDLSGFNRFALLLTGPNMGGKSTMMRATCLTIVLAQLGCY   983
SvMSH7          HPYAFAESANGLVPNDLTLGQDLSGFNRFALLLTGPNMGGKSTMMRATCLTIVLAQLGCY   983
PhMSH7          HPYAFAESANGLVPNDLTLGQDLSGLNRFALLLTGPNMGGKSTIMRATCLAIVLAQLGCY   1000
PvMSH7          HPYAFAGSANGLVPNDLTLGQDLSDLNRFALLLTGPNMGGKSTIMRATCLAIVLAQLGCY   995
                ****** . ..******::**:: . *****************:******:::******:
```

MutS domain V

```
OjMSH7          VPCQSCELTLADAIFTRIGAMDRIMSGESTFLVECTETASILENATEDSLVLLDELGRGT   1057
OiMSH7          VPCQSCELTLADAIFTRIGAMDRIMSGESTFLVECTETASILENATEDSLVLLDELGRGT   1049
BdMSH7          VPCTSCELTLADSIFTRLGAIDRIMSGESTFLVECTETASVLQNATEDSLVLLDELGRGT   1062
BsMSH7          VPCTSCELTLADSIFTRLGATDRIMSGESTFLVECTETASVLQNATEDSLVLLDELGRGT   1058
HvMSH7          VPCISCELTLADSIFTRLGATDRIMSGESTFLVECSETASVLQNATEDSLVLLDELGRGT   1053
TuMSH7-3A       VPCISCELTLADSIFTRLGATDRIMSGESTFLVECSETASVLQNATEDSLVLLDELGRGT   1055
TaMSH7-3A       VPCISCELTLADSIFTRLGATDRIMSGESTFLVECSETASVLQNATEDSLVLLDELGRGT   1055
TduMSH7-3A      VPCISCELTLADSIFTRLGATDRIMSGESTFLVECSETASVLQNATEDSLVLLDELGRGT   1055
TdiMSH7-3A      VPCISCELTLADSIFTRLGATDRIMSGESTFLVECSETASVLQNATEDSLVLLDELGRGT   1055
ScMSH7          VPCISCELTLADSIFTRLGATDRIMSGESTFLVECSETASVLQNATEDSLVLLDELGRGT   1055
TaMSH7-3B       VPCISCELTLADSIFTRLGATDRIMSGESTFLVECSETASVLQNATEDSLVLLDELGRGT   1055
TdiMSH7-3B      VPCISCELTLADSIFTRLGATDRIMSGESTFLVECSETASVLQNATEDSLVLLDELGRGT   1055
TduMSH7-3B      VPCISCELTLADSIFTRLGATDRIMSGESTFLVECSETASVLQNATEDSLVLLDELGRGT   1055
TaMSH7-3D       VPCISCELTLADSIFTRLGATDRIMSGESTFLVECSETASVLQNATEDSLVLLDELGRGT   1055
AtaMSH7-3D      VPCISCELTLADSIFTRLGATDRIMSGESTFLVECSETASVLQNATEDSLVLLDELGRGT   1055
OtMSH7          VPCQSCELTLADSIFTRLGATDRIMSGESTFLVECTETASVLQKATEDSLVLLDELGRGT   1054
SbMSH7          VPCTSCELTLADSIFTRLGATDRIMSGESTFLVECTETASVLQNATVDSLVLLDELGRGT   1057
ZmMSH7          VPCTSCELTLADSIFTRLGATDRIMTGESTFLVECTETASVLQKATVDSLVLLDELGRGT   1051
SiMSH7          VPCTSCELTLADSIFTRLGATDRIMSGESTFLVECTETASVLQNATEDSLVLLDELGRGT   1043
SvMSH7          VPCTSCELTLADSIFTRLGATDRIMSGESTFLVECTETASVLQNATEDSLVLLDELGRGT   1043
PhMSH7          VPCASCELTLADSIFTRLGATDRIMSGESTFLVECTETASVLQNATEDSLVLLDELGRGT   1060
PvMSH7          VPCTSCELTLADSIFTRLGATDRIMSGESTFLVECTETASVLQNATEDSLVLLDELGRGT   1055
                *** ********:****:** ****:*********:****:*::** *************
```

```
                            MutS domain V
OjMSH7            STFDGYAIAYAVFRHLVEAVRCRLLFATHYHPLTKEFASHPHVTLQHMACMLKPRNGGD-   1116
OiMSH7            STFDGYAIAYAVFRHLVEAVRCRLLFATHYHPLTKEFASHPHVTLQHMACMLKPRNGGD-   1108
BdMSH7            STFDGYAIAYAVFRHLVERVRCRLLFATHYRPLTKEFASHPHVILQHMACMLRPKSGSN-   1121
BsMSH7            STFDGYAIAYAVFRHLVERVRCRLLFATHYHPLTKEFASHPHVILQHMACMLRPKGGSN-   1117
HvMSH7            STFDGYAIAYAVFRHLVEQVRCRLLFATHYHPLTKEFASHPHVSLQHMACMLRPRSGGN-   1112
TuMSH7-3A         STFDGYAIAYAVFRHLVEQVRCRLLFATHYHPLTKEFASHPHVSLQHMACMLRPRSGGN-   1114
TaMSH7-3A         STFDGYAIAYAVFRHLVEQVRCRLLFATHYHPLTKEFASHPHVSLQHMACMLRPRSGGN-   1114
TduMSH7-3A        STFDGYAIAYAVFRHLVEQVRCRLLFATHYHPLTKEFASHPHVSLQHMACMLRPRSGGN-   1114
TdiMSH7-3A        STFDGYAIAYAVFRHLVEQVRCRLLFATHYHPLTKEFASHPHVSLQHMACMLRPRSGGN-   1114
ScMSH7            STFDGYAIAYAVFRHLVEQVRCRLLFATHYHPLTKEFASHPHVSLQHMACMQRPRSGGN-   1114
TaMSH7-3B         STFDGYAIAYAVFRHLVEQVRCRLLFATHYHPLTKEFASHPHVSLQHMACMLRPRSVGN-   1114
TdiMSH7-3B        STFDGYAIAYAVFRHLVEQVRCRLLFATHYHPLTKEFASHPHVSLQHMACMLRPRSVGN-   1114
TduMSH7-3B        STFDGYAIAYAVFRHLVEQVRCRLLFATHYHPLTKEFASHPHVSLQHMACMLRPRSVGN-   1114
TaMSH7-3D         STFDGYAIAYAVFRHLVEQVRCRLLFATHYHPLTKEFASHPHVSLQHMACMLRPRSGGN-   1114
AtaMSH7-3D        STFDGYAIAYAVFRHLVEQVRCRLLFATHYHPLTKEFASHPHVSLQHMACMLRPRSGGN-   1114
OtMSH7            STFDGYAIAYAVFRHLVERVGCRLLFATHYHPLTKEFASHPHVSLQHMACIFKPKSGAHG   1114
SbMSH7            STFDGYAIAYAVFRHLVERVRCRQLFATHYHSLTKEFASHPHVSLQHMACMFKPRS---D   1114
ZmMSH7            STFDGYAIAYAVFRHLVERVRCRQLFATHYHSLTKEFASHPHVSLQHMACMFKPRS---D   1108
SiMSH7            STFDGYAIAYAVFRHLVEQVRCRLLFATHYHSLTKEFASHPHVSLQHMACMFRARSGAHD   1103
SvMSH7            STFDGYAIAYAVFRHLVEQVRCRLLFATHYHSLTKEFASHPHVSLQHMACMFRARSGAHD   1103
PhMSH7            STFDGYAIAYAVFRHLVERVQCRLLFATHYHSLTKEFASHPHVSLQHMACMFRPRSGAHD   1120
PvMSH7            STFDGYAIAYAVFRHLVERVRCRLLFATHYHSLTKEFASHPHVSLQHMACMFRPRS----   1111
                  ****************** * ** ******: *********** ******: : :.

                        MutS domain V
OjMSH7            -GGEKELTFLYRLTSGACPESYGLQVATMAGLPRSIVERASAAGEMMRSKIAGNFRSSEE   1175
OiMSH7            -GGEKELTFLYRLTSGACPESYGLQVATMAGLPRSIVERASAAGEMMRSKIAGNFRSSEE   1167
BdMSH7            --GDKEITFLYRLTAGASPESYGLQVATMAGLPKSIVEKASVAGQMMKSKLTRNFKSSEG   1179
BsMSH7            --SDKDITFLYRLTSGASPESYGLQVATMAGLPKSIVEKASVAGQMMKSKITRNFKSSEG   1175
HvMSH7            --GEMELTFLYRLASGSSPESYGLQVATMAGIPKSIVEKAAVAGEMMKSRIAGNFRSSEG   1170
TuMSH7-3A         --GEMELTFLYRLASGASPESYGLQVAAMAGIPKSIVEKAAVAGEMMKSRIAGNFRSSEG   1172
TaMSH7-3A         --GEMELTFLYRLASGASPESYGLQVATMAGIPKSIVEKAAVAGEMMKSRIAGNFRSSEG   1172
TduMSH7-3A        --GEMELTFLYRLASGASPESYGLQVATMAGIPKSIVEKAAVAGEMMKSRIAGNFRSSEG   1172
TdiMSH7-3A        --GEMELTFLYRLASGASPESYGLQVATMAGIPKSIVEKAAVAGEMMKSRIAGNFRSSEG   1172
ScMSH7            --GEMELTFLYRLASGASPESYGLQVATMAGIPKSIVEKAAVAGEMMKSRIAGNFRSSEG   1172
TaMSH7-3B         --GEMELTFLYRLASGASPESYGLQVATMAGIPKSIVEKAAVAGEMMKSRIAGSFRSSEG   1172
TdiMSH7-3B        --GEMELTFLYRLASGASPESYGLQVATMAGIPKSIVEKAAVAGEMMKSRIAGSFRSSEG   1172
TduMSH7-3B        --GEMELTFLYRLASGASPESYGLQVATMAGIPKSIVEKAAVAGEMMKSRIAGSFRSSEG   1172
TaMSH7-3D         --GEMELTFLYRLVSGASPESYGLQVATMAGIPKSIVEKAAVAGEMMKSRIAGNFRSSEG   1172
AtaMSH7-3D        --GEMELTFLYRLVSGASPESYGLQVATMAGIPKSIVEKAAVAGEMMKSRIAGNFRSSEG   1172
OtMSH7            DSSEKELTFLYRLTSGACPESYGLQVATMAGLPKSIVEKASIAGQMMRSKITRNFKSSEE   1174
SbMSH7            GNGQKELTFLYRLTSGACPESYGLQVATMAGIPKSIVENASVAGQVMRSKIAENFRSSEQ   1174
ZmMSH7            GNGQKELTFLYRLTSGACPESYGLQVAAMAGIPKSIVEKASVAGQVMRAKIAGNFKSSEQ   1168
SiMSH7            VNGEKELTFLYRLASGACPESYGLQVATMAGIPKSIVDKASVAGQAMRLKIAGNFKSSEE   1163
SvMSH7            VNGEKELTFLYRLASGACPESYGLQVATMAGIPKSIVDKASVAGQAMRLKIAGNFKSSEE   1163
PhMSH7            SNGEKELTFLYRLTSGACPESYGLQVATMAGIPRSIVEKASVAGQAMRSKIAGNFKSSEE   1180
PvMSH7            -NGEKELTFLYRLASGACPESYGLQVATMAGIPRSIVEKASIAGQAMRSKIAGNFKSSEQ   1170
                     .: ::******.:*:.*********:***:*:***:.*: **: *: ::: .*:***

OjMSH7            RAEFSTLHEEWVRTIVAIGGVKDAHLDEDTMDTLFCVFHELKAHFRKRR*---------   1224
OiMSH7            RAEFSTLHEEWVRTIVAIGGVKDAHLDEDTMDTLFCVFHELKAHFRKRR----------   1216
BdMSH7            RAEFSTIHEEWLRTILAIGGVKDAHLDEDTMDTMFCISHELKAHFRKGGR*--------   1229
BsMSH7            RAEFSTLHEEWLRTILAIGGVKDAHLDEDTMDTMFCVSHELKAHFRKGGR*--------   1225
HvMSH7            RAEFSTLHEDWLQTILAIGGVKDAHLDEDTLDTMFCVAQELKSHFRKEGK*--------   1220
TuMSH7-3A         RAEFSTLHEDWLQTILAIGGVKDAHLDEDTMDTMFCVAQELKSHFRKVGR*--------   1222
TaMSH7-3A         RAEFSTLHEDWLQTILAIGGVKDAHLDEDTMDTMFCVAQELKSHFRKVGR---------   1222
TduMSH7-3A        RAEFSTLHEDWLQTILAIGGVKDAHLDEDTMDTMFCVAQELKSHFRKVGR*--------   1222
TdiMSH7-3A        RAEFSTLHEDWLQTILAIGGVKDAHLDEDTMDTMFCVAQELKSHFRKVGR*--------   1222
ScMSH7            RAEFSTLHEDWLQTILAIGGVKDAHLDEDTMDTMFCVAQELKSHFRKVGS*--------   1222
TaMSH7-3B         RAEFSTLHEDWLQTILAIGGVKDAHLDEDTMDTMFCVAQELKSHFRKVGS*--------   1222
TdiMSH7-3B        RAEFSTLHEDWLQTILAIGGVKDAHLDEDTMDTMFCVAQELKSHFRKVGS*--------   1222
TduMSH7-3B        RAEFSTLHEDWLQTILAIGGVKDAHLDEDTMDTMFCVAQELKSHFRKVGS*--------   1222
TaMSH7-3D         RAEFSTLHEDWLQTILAIGGVKDAHLDEDTMDTMFCVAQELKSHFRKGGS*--------   1222
AtaMSH7-3D        RAEFSTLHEDWLQTILAIGGVKDAHLDEDTMDTMFCVAQELKSHFRKGGS*--------   1222
OtMSH7            RAEFSTLHEEWLRTVIYISGMKEGQLDVDTMDTAFCISQELKAHFRKAR*---------   1223
SbMSH7            RAEFSTLHEERLREALAVSVQDGLL-DDDIMDTLICVRQELKSHFRKSQMSIHFRKAR*   1231
ZmMSH7            RAEFSTLHEEWLRAALAVSAMDGQPDDDDVMDTLFCIQQELKSHFRKAR*---------   1217
SiMSH7            RAAFSTQHEEWLRTAMSVIVKDGHL-DEDIMDTLFCVCQELKFHFRKARRASTATDH*-   1219
SvMSH7            RAAFSTQHEEWLRTAMSVIVKDGHL-DEDIMDTLFCVCQELKFHFRKARRASTATDH*-   1219
PhMSH7            RAEFSTRHEEWLRTAVAVIVKDGHL-DEDIMDTLYCVSQELKAHFKKAR*---------   1228
PvMSH7            RAEFATLHEEWLRTALAVIVKDGHL-DEDIMDTVYCISQELKAHFKKDR*---------   1218
                  ** *:* **: ::  : :   .    * * :**  *: :*** **:*
```

Supplementary Figure 12. Alignment of MSH7 protein sequences from various grasses. Dashes denote gaps. Amino acid positions are shown on the right. Stars below the alignment denote identical amino acids across the three sequences. Boxes highlight the conserved protein domains [3]: (1) MutS domain I (amino acid: 405-515): N-terminal mismatch-recognition domain; (2) MutS domain II (amino acid 525-672): connector domain; (3) MutS domain III (amino acid 689-905): core domain composed of two separate subdomains that join together to form a helical bundle; (4) MutS domain V (amino acid 967-1154): ATPase domain containing a Walker A motif.

Supplementary Table 1. Mutations identified in *ph2b* mutant within the genic regions located on the 121.16 Mb terminal portion of 3DS (deleted *ph2a* region).

**Position on CS Ref v1.0 Chr3D**	**Base in CS**	**SNP in *ph2b***	**Read Depth at SNP**	**Gene Model ID**	**Location of SNP in gene**	**Mutation Type**	**Predicted change**	**Functional Annotation (Ref v1.0)**
2.881.271	G	A	12	TraesCS3D01G008200	**exon**	Missense	H → Y	Transmembrane protein (DUF616)
3.628.871	G	A	23	TraesCS3D01G010300	**exon**	Missense	A → V	Cellulose synthase family protein
4.166.898	C	T	7	TraesCS3D01G011800	3'UTR			Receptor kinase
4.172.823	C	T	21	TraesCS3D01G011900	3'UTR			Receptor kinase
5.759.839	C	T	4	TraesCS3D01G016400	**exon**	Missense	A → T	Protein Detoxification
7.571.586	C	T	4	TraesCS3D02G017900LC	**exon**	Synonymous	G → G	Enhanced disease susceptibility 1 family protein
11.393.624	C	T	8	TraesCS3D01G031000	**exon**	Synonymous	H → H	Cellulose synthase family protein
12.796.433	C	T	31	TraesCS3D01G035000	**exon**	Missense	S → N	F-box domain containing protein
14.556.142	C	T	42	TraesCS3D01G039000	intron			Ethylene responsive transcription factor
15.329.872	C	T	10	TraesCS3D01G040000	**exon**	Synonymous	D → D	Leucine-rich repeat receptor-like protein kinase family protein
19.361.564	C	T	94	TraesCS3D01G050300	intron			D Ala D/L Ala epimerase
22.328.912	C	T	22	TraesCS3D01G054300	**exon**	Missense	D → N	Zinc finger family protein
23.148.830	C	T	18	TraesCS3D01G056000	**exon**	Missense	G → E	Protein Detoxification
24.157.076	T	**G**	7	TraesCS3D01G057600	intron			Photosystem I assembly protein Ycf3
25.521.235	G	A	24	TraesCS3D01G058800	intron			RNA 2' phosphotransferase
26.126.859	G	A	51	TraesCS3D01G059600	intron			Mitochondrial glycoprotein
26.301.225	G	A	10	TraesCS3D01G060500	**exon**	Missense	A → T	Receptor kinase
26.642.674	G	A	16	TraesCS3D01G061300	**exon**	Missense	A → V	Invertase/pectin methylesterase inhibitor family protein
30.299.251	G	A	18	TraesCS3D01G068900	**exon, splice region**	Synonymous	V → V	DNA/RNA helicase protein
30.674.521	G	A	20	TraesCS3D01G069700	intron in 3'UTR			Serine protease HTRA1
31.850.120	G	A	6	TraesCS3D01G071200	**exon**	Synonymous	G → G	F-box protein
32.299.605	G	A	20	TraesCS3D01G072200	**exon**	Missense	A → T	Receptor like kinase
39.391.792	C	T	36	TraesCS3D01G079300	**exon**	**Nonsense**	W → Stop	DNA directed RNA polymerase subunit beta
42.492.733	C	T	9	TraesCS3D01G083800	3'UTR			RNA recognition motif containing family protein

43.789.462	C	T	6	TraesCS3D01G086400	**exon**	Missense	P → S	Monocopper oxidase like protein SKU5
43.801.237	C	T	25	TraesCS3D01G086600	**exon**	Missense	G → E	NBS LRR disease resistance protein
45.068.019	T	C	79	TraesCS3D01G089000	intron			Sec independent protein translocase protein TatB
45.244.964	C	T	32	TraesCS3D01G089300	promoter			Signal peptidase I
46.097.272	C	T	10	TraesCS3D01G090800	**exon**	Missense	R → W	F box family protein
48.321.531	C	T	6	TraesCS3D02G096000LC	**exon**	Missense	P → L	actin binding protein
48.352.894	C	T	5	TraesCS3D01G095000	**exon**	Missense	E → K	receptor kinase 1
51.100.832	C	T	7	TraesCS3D01G099500	**exon**	Missense	V → M	Chromodomain helicase DNA binding family protein
54.697.790	C	T	9	TraesCS3D01G101800	**exon**	**Nonsense**	W → Stop	Leucine-rich repeat receptor-like protein kinase family protein
58.301.099	G	A	76	TraesCS3D01G106200	intron			helicase with zinc finger protein
61.313.146	C	T	11	TraesCS3D01G107700	**exon**	Missense	P → S	Protein IQ DOMAIN 1
61.313.469	C	T	10	TraesCS3D01G107700	**exon**	Synonymous	I → I	Protein IQ DOMAIN 2
61.568.007	C	T	5	TraesCS3D01G107900	**exon**	Synonymous	A → A	EEIG1/EHBP1 N terminal domain containing protein
71.523.376	C	T	7	TraesCS3D01G117900	intron			spatacsin carboxy terminus protein
74.359.312	G	A	17	TraesCS3D01G119400	**Intron / exon junction**	**gains in-frame STOP**	NA	DNA mismatch repair protein mutS
75.545.710	G	A	16	TraesCS3D01G119900	**exon**	Synonymous	G → G	BTB/POZ domain containing family protein
77.362.679	G	A	9	TraesCS3D02G134300LC	promoter			1-aminocyclopropane-1-carboxylate deaminase/D-cysteine desulfhydrase
81.816.245	G	A	22	TraesCS3D01G124200	**exon**	Synonymous	E → E	Protein kinase related protein
82.743.738	G	A	43	TraesCS3D01G124600	**exon**	Missense	P → S	Ubiquitinyl hydrolase 1
86.939.063	G	A	30	TraesCS3D01G128700	**exon**	Missense	A → V	Cytochrome P450
88.091.352	G	A	5	TraesCS3D01G129000	downstream of gene			Chaperone protein DnaK
88.432.345	G	A	25	TraesCS3D01G129200	**exon**	Missense	G → E	Zinc finger CCCH domain protein
89.801.581	G	A	7	TraesCS3D01G129700	**exon**	Missense	D → N	FBD associated F box protein
91.660.059	G	A	19	TraesCS3D01G131500	intron			DUF674 family protein
94.502.419	G	A	10	TraesCS3D01G152500LC	**exon**	Missense	D → N	Cell number regulator 8
96.392.397	G	A	12	TraesCS3D01G137800	**exon**	Synonymous	E → E	Pentatricopeptide repeat containing protein

96.442.973	G	A	12	TraesCS3D01G138200	promoter			Serine threonine kinase receptor associated protein
103.790.112	G	A	5	TraesCS3D01G141700	3' UTR			protein kinase family protein
105.463.143	G	A	14	TraesCS3D01G162600LC	**exon**	Synonymous	K → K	RNA-dependent RNA polymerase
105.704.314	T	A	4	TraesCS3D01G143200	promoter			Cytokinin oxidase/dehydrogenase
109.969.053	C	T	4	TraesCS3D01G145800	promoter			GDSL esterase/lipase
112.960.997	C	T	5	TraesCS3D01G148200	**exon**	Synonymous	F → F	Basic leucine zipper (bZIP) transcription factor family protein
113.519.353	C	T	12	TraesCS3D01G148400	intron			Phospho N acetylmuramoyl pentapeptide transferase like protein
114.081.057	C	T	13	TraesCS3D01G149100	**exon**	Synonymous	D → D	transmembrane protein (DUF616)
118.539.179	C	T	4	TraesCS3D01G152600	intron			Ankyrin repeat containing protein

Note : Comparison between wild-type and ph2b exome capture sequences identified 59 single nucleotide differences within the genic regions, i.e. potential promotors (2kb upstream of CDS), 5’ and 3’ UTR, introns and exons. Exonic mutations are highlighted in bold.

Supplementary Table 2. Meiotic phenotype at metaphase I of wheat / rye hybrids carrying deletions of different length on chromosome-arm 3DS.

**Lines**	**Max breakage position on 3DS** (bp)	**n**	**Univalents** (Mean ± SEM)	**Rod bivalents** (Mean ± SEM)	**Ring bivalents** (Mean ± SEM)	**Chiasma frequency** (Mean ± SEM)
CS / rye	-	32	27.25 ± 0.19	0.38 ± 0.10	0.00 ± 0.00	0.38 ± 0.10
B5L / rye	-	50	26.48 ± 0.14	0.76 ± 0.07	0.00 ± 0.00	0.76 ± 0.07
A12S / rye	19,308,909	50	26.64 ± 0.24	0.66 ± 0.11	0.02 ± 0.02	0.7 ± 0.13
A8S / rye	25,119,750	50	25.48 ± 0.26	1.16 ± 0.12	0.10 ± 0.05	1.36 ± 0.16
B6S / rye	38,035,791	50	25.76 ± 0.22	1.12 ± 0.11	0.00 ± 0.00	1.12 ± 0.11
B5S / rye	43,804,778	50	26.64 ± 0.14	0.68 ± 0.07	0.00 ± 0.00	0.68 ± 0.07
407 / rye	43,804,778	50	26.60 ± 0.14	0.70 ± 0.07	0.00 ± 0.00	0.70 ± 0.07
977 / rye	53,104,805	50	27.56 ± 0.12	0.22 ± 0.06	0.00 ± 0.00	0.22 ± 0.06
1913 / rye	53,104,805	50	26.88 ± 0.18	0.54 ± 0.09	0.02 ± 0.02	0.58 ± 0.10
731 / rye	58,559,105	50	24.28 ± 0.24	1.86 ± 0.12	0.00 ± 0.00	1.86 ± 0.12
303 / rye	62,919,181	50	26.28 ± 0.23	0.86 ± 0.12	0.00 ± 0.00	0.86 ± 0.12
A185 / rye	64,081,522	50	27.08 ± 0.19	0.38 ± 0.09	0.08 ± 0.04	0.54 ± 0.11
A6S / rye	65,616,212	50	27.44 ± 0.15	0.28 ± 0.08	0.00 ± 0.00	0.28 ± 0.08
B8S / rye	79,248,033	50	20.80 ± 0.30	3.28 ± 0.17	0.32 ± 0.08	3.92 ± 0.17
2457 / rye	82,091,343	50	22.56 ± 0.33	2.58 ± 0.17	0.14 ± 0.05	2.86 ± 0.17
2860 / rye	90,115,688	50	21.92 ± 0.25	2.90 ± 0.14	0.14 ± 0.06	3.18 ± 0.14
272 / rye	95,482,853	50	21.20 ± 0.30	3.24 ± 0.16	0.16 ± 0.05	3.56 ± 0.15
A1S / rye	105,420,612	50	20.76 ± 0.30	3.48 ± 0.16	0.14 ± 0.05	3.76 ± 0.16
A14S / rye	116,330,986	50	21.56 ± 0.28	2.98 ± 0.13	0.24 ± 0.06	3.46 ± 0.17
B7S / rye	116,330,986	50	23.32 ± 0.27	2.08 ± 0.15	0.26 ± 0.06	2.60 ± 0.15
194 / rye	116,330,986	50	21.16 ± 0.28	3.22 ± 0.15	0.20 ± 0.06	3.62 ± 0.16
ph2a / rye	125,283,114	50	22.12 ± 0.22	2.78 ± 0.11	0.16 ± 0.05	3.10 ± 0.13
1952 / rye	125,283,114	50	22.84 ± 0.25	2.42 ± 0.13	0.16 ± 0.05	2.74 ± 0.14
A130A / rye	142,617,171	50	20.92 ± 0.36	3.24 ± 0.19	0.30 ± 0.08	3.84 ± 0.20

Note : n = number of cells examined. CS: Chinese Spring wild-type. B5L: wheat deletion line carrying a 357 Mb deletion of the chromosome-arm 3DL (see Svačina et al. 4). Source data are provided as a Source Data file.

Supplementary Table 3. *Tamsh7-3D* TILLING mutants.

**Line**	**Mutation type**	**Codon**	**Position in transcript** (out of 4304)	**Amino acid**	**Position in protein** (out of 1222)
Cadenza0638	missense variant	Gct / Act	1605	A / T	467
	synonymous variant	agG / agA	3692	R	1162
Cadenza1114	missense variant	Gat / Aat	2130	D / N	642
Cadenza1178	missense variant	Ggg / Agg	1594	G / R	463
Cadenza2006	stop gained	Caa / Taa	2019	Q / *	605

Supplementary Table 4. Meiotic phenotype at metaphase I in wild-type wheat cv. Cadenza / *Aegilops variabilis* hybrids and in *Tamsh7-3D* mutant Cadenza / *Aegilops variabilis* hybrids.

**Lines**	**n**	**Univalents**	**Rod bivalents**	**Ring bivalents**	**Multivalents**	**Chiasma frequency**	***p* value**
Cadenza WT / *Ae.variabilis* (1)	57	32.82 ± 0.29 (27-35)	1.09 ± 0.14 (0-4)	0.00 ± 0	0.00 ± 0	1.09 ± 0.14 (0-4)	
Cadenza WT / *Ae.variabilis* (2)	79	32.77 ± 0.23 (27-35)	1.11 ± 0.12 (0-4)	0.00 ± 0	0.00 ± 0	1.11 ± 0.12 (0-4)	
Mean	136	32.79 ± 0.18 (27-35)	1.10 ± 0.09 (0-4)	0.00 ± 0	0.00 ± 0	**1.10 ± 0.09** (0-4)	-
msh7-3D A467T / *Ae.variabilis* (1)	52	29.54 ± 0.48 (21-35)	2.71 ± 0.24 (0-7)	0.02 ± 0.02 (0-1)	0.00 ± 0	2.75 ± 0.24 (0-7)	
msh7-3D A467T / *Ae.variabilis* (2)	50	30.84 ± 0.41 (23-35)	2.06 ± 0.20 (0-5)	0.02 ± 0.02 (0-1)	0.00 ± 0	2.10 ± 0.21 (0-7)	
Mean	102	30.18 ± 0.32 (21-35)	2.39 ± 0.16 (0-7)	0.02 ± 0.01 (0-1)	0.00 ± 0	**2.43 ± 0.16** (0-7)	2.30×10^{-10}
msh7-3D G463R / *Ae.variabilis* (1)	59	30.83 ± 0.39 (23-35)	2.02 ±0.18 (0-5)	0.07 ± 0.04 (0-2)	0.00 ± 0	2.15 ± 0.21 (0-8)	
msh7-3D G463R / *Ae.variabilis* (2)	62	30.97 ± 0.34 (25-35)	2.00 ± 0.17 (0-5)	0.02 ± 0.02 (0-1)	0.00 ± 0	2.03 ± 0.17 (0-5)	
Mean	121	30.90 ± 0.25 (23-35)	2.01 ± 0.12 (0-5)	0.04 ± 0.02 (0-2)	0.00 ± 0	**2.09 ± 0.14** (0-8)	7.15×10^{-8}
msh7-3D D642N / *Ae.variabilis* (1)	57	29.70 ± 0.38 (21-35)	2.61 ± 0.19 (0-7)	0.04 ± 0.02 (0-1)	0.00 ± 0	2.68 ± 0.20 (0-7)	
msh7-3D D642N / *Ae.variabilis* (2)	62	30.13 ± 0.37 (23-35)	2.34 ± 0.18 (0-6)	0.10 ± 0.04 (0-1)	0.00 ± 0	2.53 ± 0.19 (0-6)	
Mean	119	29.92 ± 0.26 (21-35)	2.47 ± 0.13 (0-7)	0.07 ± 0.02 (0-1)	0.00 ± 0	**2.61 ± 0.14** (0-7)	1.25×10^{-15}
msh7-3D Q605* / *Ae.variabilis* (1)	48	23.29 ± 0.54 (15-33)	5.31 ± 0.27 (1-10)	0.35 ± 0.08 (0-2)	0.13 ± 0.05 (0-1)	6.27 ± 0.30 (1-10)	
msh7-3D Q605* / *Ae.variabilis* (2)	73	23.70 ± 0.36 (17-29)	5.27 ± 0.18 (3-9)	0.21 ± 0.05 (0-1)	0.11 ± 0.04 (0-1)	5.93 ± 0.21 (3-10)	
Mean	121	23.54 ± 0.31 (15-33)	5.29 ± 0.15 (1-10)	0.26 ± 0.04 (0-2)	0.12 ± 0.03 (0-1)	**6.07 ± 0.17** (1-10)	7.43×10^{-42}

Note : n = number of cells examined. Two-sided Mann-Whitney tests adjusted for multiple comparisons were performed to test for significant differences between wild-type and Tamsh7-3D mutant hybrids. Mean ± SEM (range). Source data are provided as a Source Data file.

Supplementary Table 5. Meiotic phenotype at metaphase I in wild-type wheat cv. Chinese Spring (CS WT), *ph2b* (CS *ph2b*), B8S 3DS-deletion line (CS B8S), wild-type wheat cv. Cadenza (Cadenza WT), homozygous and heterozygous *Tamsh7-3D* mutants.

**Lines**	**n**	**Univalents**	**Rod bivalents**	**Ring bivalents**	**Multivalents**	**Chiasma frequency**	***p* value**
CS WT	50	0.04 ± 0.04 (0-2)	0.98 ± 0.13 (0-4)	20.00 ± 0.13 (17-21)	0.00 ± 0	**40.98 ± 0.14** (38-42)	-
CS *ph2b*	61	1.75 ± 0.18 (0-6)	2.74 ± 0.16 (0-6)	17.33 ± 0.20 (14-21)	0.03 ± 0.02 (0-1)	**37.48 ± 0.26** (32-42)	3.04×10^{-15}
CS B8S	52	1.73 ± 0.15 (0-4)	2.93 ± 0.15 (0-6)	17.15 ± 0.17 (14-20)	0.04 ± 0.03 (0-1)	**37.31 ± 0.20** (34-40)	1.00×10^{-16}
Cadenza WT	50	0.28 ± 0.10 (0-2)	1.00 ± 0.14 (0-4)	19.86 ± 0.13 (17-21)	0.00 ± 0	40.72 ± 0.15 (38-42)	
Cadenza WT	60	0.10 ± 0.06 (0-2)	0.78 ± 0.10 (0-3)	20.17 ± 0.10 (18-21)	0.00 ± 0	41.12 ± 0.11 (39-42)	
Mean	110	0.18 ± 0.06 (0-2)	0.88 ± 0.08 (0-4)	20.03 ± 0.08 (17-21)	0.00 ± 0	**40.94 ± 0.09** (38-42)	-
msh7-3D A467T -/- (1)	50	0.08 ± 0.06 (0-2)	0.96 ± 0.16 (0-4)	20.00 ± 0.17 (17-21)	0.00 ± 0	40.96 ± 0.18 (37-42)	
msh7-3D A467T -/- (2)	55	0.25 ± 0.10 (0-4)	1.00 ± 0.12 (0-3)	19.87 ± 0.13 (18-21)	0.00 ± 0	40.75 ± 0.15 (37-42)	
Mean	105	0.17 ± 0.06 (0-4)	0.98 ± 0.10 (0-4)	19.93 ± 0.10 (17-21)	0.00 ± 0	**40.85 ± 0.12** (37-42)	1.00
msh7-3D G463R -/- (1)	51	0.16 ± 0.08 (0-2)	1.06 ± 0.14 (0-3)	19.86 ± 0.14 (17-21)	0.00 ± 0	40.78 ± 0.16 (37-42)	
msh7-3D G463R -/- (2)	62	0.23 ± 0.09 (0-4)	1.24 ± 0.14 (0-4)	19.65 ± 0.15 (17-21)	0.00 ± 0	40.53 ± 0.18 (37-42)	
Mean	113	0.19 ± 0.06 (0-4)	1.16 ± 0.10 (0-4)	19.74 ± 0.11 (17-21)	0.00 ± 0	**40.65 ± 0.12** (37-42)	0.99
msh7-3D D642N -/- (1)	55	1.36 ± 0.22 (0-8)	2.05 ± 0.16 (0-4)	18.16 ± 0.20 (15-21)	0.05 ± 0.03 (0-1)	38.56 ± 0.28 (33-42)	
msh7-3D D642N -/- (2)	55	0.58 ± 0.12 (0-2)	1.98 ± 0.14 (0-6)	18.73 ± 0.15 (15-21)	0.00 ± 0	39.44 ± 0.18 (36-42)	
Mean	110	0.97 ± 0.13 (0-8)	2.02 ± 0.11 (0-6)	18.45 ± 0.13 (15-21)	0.03 ± 0.02 (0-1)	**39.00 ± 0.17** (33-42)	1.80×10^{-18}
msh7-3D Q605* -/- (1)	42	2.24 ± 0.29 (0-8)	3.00 ± 0.29 (0-9)	16.83 ± 0.30 (11-20)	0.02 ± 0.02 (0-1)	36.76 ± 0.37 (31-41)	
msh7-3D Q605* -/- (2)	26	1.38 ± 0.33 (0-6)	3.08 ± 0.24 (1-6)	17.15 ± 0.26 (15-20)	0.04 ± 0.04 (0-1)	37.50 ± 0.36 (34-41)	
msh7-3D Q605* -/- (3)	41	1.37 ± 0.17 (0-4)	2.12 ± 0.17 (0-5)	18.12 ± 0.16 (15-20)	0.05 ± 0.03 (0-1)	38.46 ± 0.19 (35-41)	
Mean	109	1.71 ± 0.16 (0-8)	2.69 ± 0.15 (0-9)	17.39 ± 0.15 (11-20)	0.04 ± 0.02 (0-1)	**37.58 ± 0.19** (31-41)	7.66×10^{-31}
msh7-3D Q605* +/-	46	1.65 ± 0.11 (0-2)	1.26 ± 0.16 (0-4)	18.91 ± 0.17 (16-21)	0.00 ± 0	**39.09 ± 0.19** (36-42)	5.63×10^{-13}

Note : n = number of cells examined. Two-sided Mann-Whitney tests adjusted for multiple comparisons were performed to test for significant differences between mutant lines and corresponding wild-type (CS or Cadenza). Mean ± SEM (range). Source data are provided as a Source Data file.

Supplementary Table 6. Fifteen mutated genes in common between *ph2b* and *Tamsh7-3D* Q605*, additional to *TaMSH7-3D.*

Gene ID	Chromosome	in *ph2b*						in *Tamsh7-3D* Q605*						Functional Annotation (Ref v1.0)
		Mutated position	Ref. base	SNP	Zygocity	Mutation Type	Predicted change	Mutated position	Ref. base	SNP	Zygocity	Mutation Type	Predicted change	
TraesCS1B02G068000	chr1B	52 899 611	**C**	T	hom	Missense	A -> V	52 901 362	**C**	T	hom	Missense	P -> S	WEB family protein, chloroplastic
TraesCS1B02G279100	chr1B	487 426 136	**G**	A	hom	Missense	P -> L	487 426 436	**G**	A	hom	Missense	G -> D	Isoflavone reductase-like protein
TraesCS1B02G379800	chr1B	612 987 615	**C**	T	hom	Missense	P -> S	612 989 415	**G**	A	het	Missense	G -> D	Homogentisate phytyltransferase
TraesCS2A02G546300	chr2A	755 119 039	**G**	A	hom	Missense	A -> T	na	**G**	A	het	Missense	G -> E	Cysteine proteinase
TraesCS2B02G304600	chr2B	433 538 918	**G**	A	hom	Missense	A -> T	433 541 213	**G**	A	het	Missense	R -> K	Mei2-like protein
TraesCS2B02G595300	chr2B	779 345 847	**G**	A	hom	Missense	R -> C	779 345 122	**C**	T	het	Stop Gained	W -> *	Allantoinase
TraesCS3A02G224200	chr3A	419 542 022	**G**	A	hom	Missense	A -> V	na	**G**	A	het	Missense	S -> F	Extra-large G-like protein
TraesCS3D02G266400	chr3D	369 800 226	**G**	A	hom	Missense	G -> E	369 792 209	**G**	A	hom	Missense	G -> D	Glutamate synthase
TraesCS4A02G315800	chr4A	605 732 209	**G**	A	hom	Missense	R -> K	605 732 739	**C**	T	het	Missense	L -> F	Mediator of RNA polymerase II transcription subunit 13
TraesCS4A02G394100	chr4A	669 443 368	**G**	A	hom	Missense	P -> L	na	**G**	A	het	Missense	M -> I	Methionine S-methyltransferase
TraesCS5D02G302900	chr5D	398 807 551	**C**	T	hom	Stop Gained	W -> *	398 806 964	**C**	T	het	Missense	G -> D	Beta-glucosidase
TraesCS5D02G451000	chr5D	499 411 259	**G**	A	hom	Missense	A -> T	499 411 666	**C**	T	het	Missense	S -> L	WRKY transcription factor, putative
TraesCS6B02G375500	chr6B	649 914 771	**G**	A	hom	Missense	D -> N	649 919 501	**G**	A	het	Missense	S -> N	Protein TONSOKU
TraesCS7B02G189300	chr7B	323 207 354	**C**	T	hom	Missense	S -> N	323 207 794	**G**	A	hom	Missense	P -> L	Topless-related protein 1
TraesCS7D02G195400	chr7D	151 916 288	**G**	A	hom	Missense	A -> V	151 916 448	**C**	T	hom	Missense	A -> T	Peroxidase

Supplementary Table 7. Pollen viability as determined by Alexander staining in wild-type and *Tamsh7-3D* Q605*.

Gentotype	Anther	Viable	Inviable	Total	% viable	% inviable	*p* value
Cadenza WT	1	606	56	662	91.5	8.5	
	2	668	76	744	89.8	10.2	
	3	992	52	1044	95.0	5.0	
	4	1036	62	1098	94.4	5.6	
	5	1056	31	1087	97.1	2.9	
	6	805	71	876	91.9	8.1	
	7	905	59	964	93.9	6.1	
	8	1071	59	1130	94.8	5.2	n.d.
Tamsh7-3D Q605*	1	933	129	1062	87.9	12.1	
	2	893	138	1031	86.6	13.4	
	3	904	136	1040	86.9	13.1	
	4	897	148	1045	85.8	14.2	
	5	845	185	1030	82.0	18,0	
	6	702	106	808	86.9	13.1	
	7	803	150	953	84.3	15.7	
	8	924	171	1095	84.4	15.6	6×10^{-6}

Note : Pollen viability of 8 anthers per genotype was assessed using Alexander staining for at least 600 pollen grains derived from mature flowers. Viable pollen grains stained purple, whereas non-viable pollen stained green. Pairwise t-test (two-sided) adjusted for multiple testing was used for determining significance.

Supplementary Table 8. Seed set per spike in wild-type versus *Tamsh7-3D* Q605*.

Genotype	Plant	Seed number per spike						Mean	*p* value
		1	2	3	4	5	6		
Cadenza WT	1	19	11	46	42	55	77	41.67	
	2	28	0	6	22	49	52	26.17	
	3	71	75	98	76	86	82	81.33	
	4	77	65	95	85	77	86	80.83	n.d.
Tamsh7-3D Q605*	1	16	32	23	32	27	15	24.17	
	2	37	33	37	58	54	61	46.67	
	3	79	100	86	62	54	59	73.33	
	4	65	77	89	85	82	85	80.50	0.43

Note : The number of seeds were counted for the first 6 spikes of 4 separate plants per genotype. Pairwise t-test (two-sided) adjusted for multiple testing was used for determining significance. Mean values per individual were compared.

Supplementary Table 9. Pairwise percentages of nucleotide and amino acid sequence identities across TaMSH7 homoeologues.

	**TaMSH7-3A**	**TaMSH7-3B**	**TaMSH7-3D**
**TaMSH7-3A**		96.32	96.32
**TaMSH7-3B**	97.77		97.22
**TaMSH7-3D**	97.77	97.96	

Note : Values in the bottom left half of the data field represent nucleotide sequence identity (in blue), and values in the top right half represent amino acid sequence identity (in green).

Supplementary Table 10. Conservation of *TaMSH7-3A, TaMSH7-3B* and *TaMSH7-3D* genes among 436 varieties of hexaploid bread wheat (*Triticum aestivum* L.).

Chromosome	Position	Reference CS	Alternative	Number of varieties alt	Gene	Location	AA change
chr3A	87374581	Ggg	Agg	10	*TaMSH7-3A*	exon	G → R
	87374890	T	C	10	*TaMSH7-3A*	intron	
	87377078	C	A	10	*TaMSH7-3A*	intron	
	87379850	CAAAAAA	CAAAAA	10	*TaMSH7-3A*	intron	
	87380264	A	C	10	*TaMSH7-3A*	exon	synonymous
	87380360	G	A	10	*TaMSH7-3A*	exon	synonymous
	87380643	A	C	10	*TaMSH7-3A*	intron	
	87380842	A	G	10	*TaMSH7-3A*	exon	H → R
	87380881	G	A	10	*TaMSH7-3A*	intron	
	87381023	C	A	10	*TaMSH7-3A*	exon	synonymous
	87381676	ATTTTTT	ATTTTT	10	*TaMSH7-3A*	intron	
	87381902	G	A	10	*TaMSH7-3A*	intron	
chr3B	119464057	T	G	408	*TaMSH7-3B*	upstream	
	119464143	G	A	56	*TaMSH7-3B*	upstream	
	119464235	C	T	408	*TaMSH7-3B*	upstream	
	119464328	A	G	56	*TaMSH7-3B*	upstream	
	119464332	T	G	325	*TaMSH7-3B*	upstream	
	119464503	A	T	408	*TaMSH7-3B*	upstream	
	119464734	A	G	57	*TaMSH7-3B*	upstream	
	119464803	G	C	57	*TaMSH7-3B*	upstream	
	119464814	A	G	408	*TaMSH7-3B*	upstream	
	119464816	C	T	74	*TaMSH7-3B*	upstream	
	119464868	G	A	45	*TaMSH7-3B*	upstream	
	119464893	C	T	32	*TaMSH7-3B*	upstream	
	119464934	AC	A	383	*TaMSH7-3B*	upstream	
	119465355	G	A	324	*TaMSH7-3B*	intron	
	119465526	C	T	407	*TaMSH7-3B*	intron	
	119465609	A	T	348	*TaMSH7-3B*	intron	
	119465722	G	A	57	*TaMSH7-3B*	intron	
	119469762	T	G	57	*TaMSH7-3B*	intron	
	119470792	C	A	348	*TaMSH7-3B*	intron	
	119470795	T	C	407	*TaMSH7-3B*	intron	
	119471556	G	A	407	*TaMSH7-3B*	intron	
	119467131	C	T	407	*TaMSH7-3B*	exon	synonymous
	119469971	A	G	321	*TaMSH7-3B*	exon	synonymous
	119472974	C	T	31	*TaMSH7-3B*	exon	synonymous
	119473306	A	G	57	*TaMSH7-3B*	intron	
	119473676	A	C	407	*TaMSH7-3B*	intron	
	119473707	C	G	57	*TaMSH7-3B*	intron	
	119473747	G	T	57	*TaMSH7-3B*	intron	
	119473786	C	T	57	*TaMSH7-3B*	intron	
	119473800	T	C	411	*TaMSH7-3B*	intron	

chr3D	74363290	G	A	3	*TaMSH7-3D*	intron	
	74363630	A	C	3	*TaMSH7-3D*	intron	
	74363715	A	G	3	*TaMSH7-3D*	intron	

Note : Genetic variants identified within the three genes (CDS ± 1 kb) are reported. Chinese Spring (CS) variety is used as a reference. Data is accessible at https://urgi.versailles.inra.fr/jbrowseiwgsc/gmod_jbrowse/ (IWGSC RefSeq v1.0; track “SNPs/Whealbi SNPs/Imputed”).

Supplementary Table 11. Conservation of the 28 bp deletion identified within *TaMSH7-3A* gene in wheat varieties. Among the varieties for which genomic data are available, 14 carry the same 28 bp deletion at position 87.374.014 on chromosome 3A.

**Accession**	**Deletion**	**Detected in resource**	**Pedigree**	**Source**
AC-Barrie	yes	DAWN	NEEPAWA/COLUMBUS(CID-188385)//BW-90	GRIS
Austro-Bankut	yes	SRA	(S)BANKUTI-1201; BANKUTI/MARQUIS; THEISS/MARQUIS; (S)BANKUTI-5	GRIS
Bankuti 1201	yes	SRA	MARQUIS/BANKUTI-5; BANKUTI-5/MARQUIS	GRIS
Cadenza	yes	10+ genomes	Axona x Tonic	a
CDC Landmark	yes	10+ genomes	Unity//Alsen/Superb	GRIS
CDC Stanley	yes	10+ genomes	W-95132/AC-BARRIE	GRIS
Coronation	yes	SRA	Marquis x Pentad	GRIS
Gladius	yes	DAWN	(DH)RAC-875/KRICHAUFF//EXCALIBUR/KUKRI/3/RAC-875/KRICHAUFF/4/RAC-875//EXCALIBUR/KUKRI;	GRIS
Julius	yes	10+ genomes	Asketis/Drifter	b
Kolben 3	yes	SRA	unknown; landrace from Sweden	
Mocho de Espiga Quadrada	yes	SRA	unknown; Portugal	
Paragon	yes	10+ genomes	CSW-1724-19-5-68//AXONA/TONIC	GRIS
RAC875	yes	DAWN	RAC-655//SR21/4*LANCE/3/4*BAYONET	GRIS
Rongotea	yes	SRA	RAVEN/(MEX)1966-ISWRN-430	GRIS
ArinaLrFor	no	10+ genomes	Arina (MOISSON/ZENITH,CHE) x Forno, BC2F5	c
Baxter	no	DAWN	INIA-66/GAMUT//COOK/4/JUPATECO/3/LERMA-ROJO-64/SONORA-64-A//(SIB)TIMGALEN [2854][3766]; QT-2327/COOK//QT-2804	GRIS
Chara	no	DAWN	BD-225/CD-87	GRIS
Claire	no	10+ genomes	WASP/FLAME	GRIS
Drysdale	no	DAWN	HARTOG*3/QUARRION	GRIS
Excalibur	no	DAWN	RAC-177(Sr26)/UNICULM-492//RAC-311-S	GRIS
H-45	no	DAWN	KALYANSONA/BLUEBIRD//ANZA*3/WW-80/3/OLYMPIC*2/CIANO-67; B-1814//WW-15/QT-7605	GRIS
Jagger	no	10+ genomes	KS-82-W-418/STEPHENS	GRIS
Kukri	no	DAWN	MADDEN/6*RAC-177//GRAJO/76-ECN-44; SR13*3//76-ECN-44/76-ECN-36; CO-1213/RAC-549; DRP((FRONTANA/KENYA-58//N10B/G55)NAINARI-60)/(TOBARI-66/CIANO-67//TOBARI-66/8156/3/CALIDAD//BLUEBIRD/CIANO-67)/2/MADDEN/6*RAC-177	GRIS
Lancer	no	10+ genomes	IMPROVED-FIFE/ROMA	GRIS
Mace	no	10+ genomes	WYALKATCHEM/STYLET//WYALKATCHEM	GRIS
Norin61	no	10+ genomes	FUKUOKAKOMUGI-18/SHINCHUNAGA; FUKUOKA-18/SHINCHUCHO	GRIS
Pastor	no	DAWN	PFAU/SERI-82(CID-93300//BOBWHITE	GRIS
Robigus	no	10+ genomes	Z-836/1366; Z-836/PUTCH	GRIS
SY-Mattis	no	10+ genomes	*not available*	
Volcani-DD	no	DAWN	V-761-28-J4-B2-N28	GRIS

Westonia	no	DAWN	SPICA/TIMGALEN//TOSCA/3/CRANBROOK//BOB-WHITE*2/JACUP	GRIS
Wyalkatchem	no	DAWN	MACHETE/3/(84-W-129-504)GUTHA//JACUP*2/11th-ISEPTON-135	GRIS
Xiaoyan-54	no	DAWN	(S)XIAOYAN-6; ST-2422-464/XIAOYAN-96	GRIS
Yitpi	no	DAWN	C-8-MMC-8-HMM/FRAME	GRIS

a: http://organicresearchcentre.com/manage/authincludes/article_uploads/Research/Plant%20breeding/parents.pdf
b: https://link.springer.com/content/pdf/10.1556/0806.44.2016.012.pdf
c: https://www.zora.uzh.ch/id/eprint/129349/1/complete_thesis_V4.pdf (page 21)

Supplementary Table 12. MSH7 sequences used in this study.

Name	Species	Gene ID	CDS length	Protein length	Genome assembly	DB source	Comment	Weblink
TaMSH7-3A	*Triticum aestivum* cv. Chinese Spring	*TraesCS3A01G117500*	3669	1222	Cs Ref v1.0	IWGSC		http://www.wheatgenome.org/
TaMSH7-3B	*Triticum aestivum* cv. Chinese Spring	*TraesCS3B01G136600*	3669	1222	Cs Ref v1.0	IWGSC		http://www.wheatgenome.org/
TaMSH7-3D	*Triticum aestivum* cv. Chinese Spring	*TraesCS3D01G119400*	3669	1222	Cs Ref v1.0	IWGSC		http://www.wheatgenome.org/
TdiMSH7-3A	*Triticum turgidum ssp. dicoccoides* cv. Zavitan	*n/a*	3669	1222	Zavitan WEWSeq v2.0 assembly	Graingenes	gene model was derived from genomic sequence	https://wheat.pw.usda.gov/GG3/
TdiMSH7-3B	*Triticum turgidum ssp. dicoccoides* cv. Zavitan	*n/a*	3669	1222	Zavitan WEWSeq v2.0 assembly	Graingenes	gene model was derived from genomic sequence	https://wheat.pw.usda.gov/GG3/
TduMSH7-3A	*Triticum turgidum ssp. durum* cv. Svevo	*n/a*	3669	1222	Svevo v1.0, 2019	Graingenes	gene model was derived from genomic sequence	https://wheat.pw.usda.gov/GG3/
TduMSH7-3B	*Triticum turgidum ssp. durum* cv. Svevo	*n/a*	3669	1222	Svevo v1.0, 2019	Graingenes	gene model was derived from genomic sequence	https://wheat.pw.usda.gov/GG3/
TuMSH7-3A	*Triticum urartu*	*n/a*	3669	1222	*T.urartu*, assembly v1.0	MBKBASE	gene model was derived from genomic sequence	http://www.mbkbase.org/Tu/
AtaMSH7-3D	*Aegilops tauschii*	*n/a*	3669	1222	BGI assembly, v1.0	Graingenes	gene model was derived from genomic sequence	https://wheat.pw.usda.gov/GG3/
BdMSH7	*Brachypodium distachyon*	*2g05160.1*	3690	1229	*Brachypodium distachyon* annotation v3.1, assemlby 3.0 (strain Bd21)	Phytozome 12		
BsMSH7	*Brachypodium stacei*	*01G360800.1*	3678	1225	*Brachypodium stacei* annotation v1.1, assembly v1.0	Phytozome 12		
HvMSH7	*Hordeum vulgare* cv. Morex	*r2.3HG0198110.1*	3663	1220	*Hordeum sativa*, cv. Morex assembly v2	IPK Gatersleben	sequence was truncated at 5'end	https://doi.ipk-gatersleben.de/DOI/83e8e186-dc4b-47f7-a820-28ad37cb176b/d1067eba-1d08-42e2-85ec-66bfd5112cd8/2
OtMSH7	*Oropetium thomaeum*	*20150105_07306A*	3672	1223	*Oropetium thomaeum* v1.0	Phytozome 12		
OiMSH7	*Oryza sativa ssp. indica*	*B8ADK6_ORYSI*	3651	1216	*Oryza sativa ssp. indica*, ASM465 v1	Ensembl/Uniprot		
OjMSH7	*Oryza sativa ssp. japanica*	*Os01g08540.1*	3675	1224	*Oryza sativa* MSU Release 7	Phytozome 12		
PhMSH7	*Panicum hallii*	*E04211.1*	3687	1228	*Panicum hallii* assembly v2.0	Phytozome 12		
PvMSH7	*Panicum virgatum*	*Ea00315.1*	3657	1218	*Panicum virgatum* annotation v1.1; assembly v1.0	Phytozome 12		
ScMSH7	*Secale cereale*	*n/a*	3669	1222	*Secale cereale* cv. Lo7 v2.0	Graingenes	gene model was derived from genomic sequence	https://wheat.pw.usda.gov/GG3/
SiMSH7	*Setaria italica*	*5G128200.1*	3660	1219	*Setaria italica* annotation v2.2, assembly v2	Phytozome 12		
SvMSH7	*Setaria viridis*	*5G125400.1*	3660	1219	*Setaria viridis* annotation v1.1, assembly v1.0	Phytozome 12		
SbMSH7	*Sorghum bicolor*	*003G041700.1*	3696	1231	*Sorghum bicolor* annotation v3.1.1, assembly v3.0.1	Phytozome 12		
ZmMSH7	*Zea mays*	*Zm00008a010917*	3654	1217	*Zea mays* PH207 v1.1, assembly v1.0	Phytozome 12	gene model was manually corrected	http://plants.ensembl.org/Oryza_indica/Info/Index

Supplementary Table 13. Primers used in this study.

**Primer name**	**Sequence (5' → 3')**
Cad_0638_wt	tttcagagagtgggattgacAatG
Cad_0638_mut	tttcagagagtgggattgacAatA
Cad_0638_common	actgtgcatgaacttgctgG
Cad_1114_wt	agattcttggCtatctgcattgG
Cad_1114_mut	agattcttggCtatctgcattgA
Cad_1114_common	agtgcaCagataactgcatctT
Cad_1178_wt	tgaaggttggtatttcagagagtG
Cad_1178_mut	tgaaggttggtatttcagagagtA
Cad_1178_common	gctaaaagcttttcaacagcatT
Cad_2006_wt	tgcaggctctgtgaaaatgC
Cad_2006_mut	tgcaggctctgtgaaaatgT
Cad_2006_common	gaatttgtgaggcatcagagaaaT
MSH7-3A-F1	ctgttccctgaacttggtta
MSH7-3A-F2	acgttcaagctaattcagtg
MSH7-3A-R1	gcttcaaacactgaattagc
MSH7-3A-R2	taacggatcctgggacggt

Supplementary references

1. Lamers, M. H. *et al.* The crystal structure of DNA mismatch repair protein MutS binding to a G·T mismatch. *Nature* **407**, 711–717 (2000).

2. Martín, A. C. *et al.* Genome-wide transcription during early wheat meiosis is independent of synapsis, ploidy level, and the *Ph1* locus. *Front. Plant Sci.* **9**, 1791 (2018).

3. Obmolova, G., Ban, C., Hsieh, P. & Yang, W. Crystal structures of mismatch repair protein MutS and its complex with a substrate DNA. *Nature* **407**, 703–710 (2000).

4. Svačina, R. *et al.* Development of deletion lines for chromosome 3D of bread wheat. *Front. Plant Sci.* **10**, 1–6 (2020).
