# Supplementary material for: Ph2 encodes the mismatch repair protein MSH7-3D that inhibits wheat homoeologous recombination
Source: Nat Commun. 2021 Feb 5;12:803. doi: 10.1038/s41467-021-21127-1 (PMC7865012; doi:10.1038/s41467-021-21127-1)
Supplement: Supplementary file 3 — Reporting Summary [file 41467_2021_21127_MOESM3_ESM.pdf]

nature research

DBPR NCOMMS-20-30516
Corresponding author(s): Heidi Serra
Pierre Sourdille

Last updated by author(s): Jan 8, 2021

Reporting Summary

Nature Research wishes to improve the reproducibility of the work that we publish. This form provides structure for consistency and transparency in reporting. For further information on Nature Research policies, see our Editorial Policies and the Editorial Policy Checklist.

Statistics

For all statistical analyses, confirm that the following items are present in the figure legend, table legend, main text, or Methods section.

n/a	Confirmed	
☐	☒	The exact sample size (*n*) for each experimental group/condition, given as a discrete number and unit of measurement
☐	☒	A statement on whether measurements were taken from distinct samples or whether the same sample was measured repeatedly
☐	☒	The statistical test(s) used AND whether they are one- or two-sided<br>*Only common tests should be described solely by name; describe more complex techniques in the Methods section.*
☒	☐	A description of all covariates tested
☒	☐	A description of any assumptions or corrections, such as tests of normality and adjustment for multiple comparisons
☐	☒	A full description of the statistical parameters including central tendency (e.g. means) or other basic estimates (e.g. regression coefficient) AND variation (e.g. standard deviation) or associated estimates of uncertainty (e.g. confidence intervals)
☐	☒	For null hypothesis testing, the test statistic (e.g. F, t, r) with confidence intervals, effect sizes, degrees of freedom and P value noted<br>*Give P values as exact values whenever suitable.*
☒	☐	For Bayesian analysis, information on the choice of priors and Markov chain Monte Carlo settings
☒	☐	For hierarchical and complex designs, identification of the appropriate level for tests and full reporting of outcomes
☒	☐	Estimates of effect sizes (e.g. Cohen's d, Pearson's r), indicating how they were calculated

Our web collection on statistics for biologists contains articles on many of the points above.

Software and code

Policy information about availability of computer code

Data collection

• Wheat 10+ Genomes sequences
BLASTN: https://webblast.ipk-gatersleben.de/wheat_ten_genomes/ and https://wheatis.earlham.ac.uk//grassroots-portal/blast
• Bioplatforms Australia WGS sequence data of wheat cultivars:
https://data.bioplatforms.com/organization/bpa-wheat-cultivars
Visualized in DAWN: http://crobiad.agwine.adelaide.edu.au/dawn/jbrowse/
• Tilling mutants of Chinese Spring: http://www.wheat-tilling.com/
• Wheat pedigree database GRIS: http://wheatpedigree.net/
• Previously generated RNASeq data: http://wheat-urgi.versailles.inra.fr/Seq-Repository/Expression
• Genetic variation in TaMSH7 genes was identified at https://urgi.versailles.inra.fr/jbrowseiwgsc/gmod_jbrowse/
• TaMSH7 homologs were retrieved at Graingenes (https://wheat.pw.usda.gov/GG3/), MBKBase (http://www.mbkbase.org/Tu/), IPK (https://doi.ipk-gatersleben.de/DOI/83e8e186-dc4b-47f7-a820-28ad37cb176b/d1067eba-1d08-42e2-85ec-66bfd5112cd8/2), IWGSC (http://www.wheatgenome.org/) and Phytozome (https://phytozome.jgi.doe.gov/pz/portal.html)

Data analysis

• Processing of NGS data – Exome data
o Quality: FASTQC version 0.11.4
o Adapter Trimming: Trimmomatic version 0.36
o Read alignment: Bowtie2 version 2.3.0
o Removal of PCR duplicates in exome capture data: Wheatbio.jar [https://github.com/CroBiAd/TILLinG-mutants]
o SNP/Indel calling: Samtools version 1.4.1
o Mutation prediction: SNPeff version 4.3
• Processing of NGS data – RNASeq
o Quality: FASTQC version 0.11.4

o Adapter Trimming: fastp version 0.19.7
o Read alignment: STAR version 2.5.3
• Protein modelling: i-TASSER at https://zhanglab.ccmb.med.umich.edu/I-TASSER/
• Protein model visualization: PyMOL version 2.3.4

For manuscripts utilizing custom algorithms or software that are central to the research but not yet described in published literature, software must be made available to editors and reviewers. We strongly encourage code deposition in a community repository (e.g. GitHub). See the Nature Research guidelines for submitting code & software for further information.

Data

Policy information about availability of data

All manuscripts must include a data availability statement. This statement should provide the following information, where applicable:

- Accession codes, unique identifiers, or web links for publicly available datasets
- A list of figures that have associated raw data
- A description of any restrictions on data availability

Data availability
• Bioproject PRJNA648242 is available at https://www.ncbi.nlm.nih.gov/sra/PRJNA648242
• Exome capture data: SRR12315376 and SRR12315377
• RNASeq data: SRR13364312 for ph2b and SRR13364311 for Chinese Spring wildtype
• TaMSH7-3D genomic sequence from ph2b : Supplementary_Data_1
• TaMSH7-3A genomic sequence from Cadenza: Supplementary_Data_1

Code availability
• Wheatbio.jar at https://github.com/CroBiAd/TILLinG-mutants

Field-specific reporting

Please select the one below that is the best fit for your research. If you are not sure, read the appropriate sections before making your selection.

[x] Life sciences   [ ] Behavioural & social sciences   [ ] Ecological, evolutionary & environmental sciences

For a reference copy of the document with all sections, see nature.com/documents/nr-reporting-summary-flat.pdf

Life sciences study design

All studies must disclose on these points even when the disclosure is negative.

Sample size	Sample size was chosen in agreement with standard methodology in the field.
Data exclusions	No data exclusions
Replication	Chiasma number was scored in ~ 50 independent meiocytes par individual, and in two individuals per genotype, when possible. Replication always gave similar results.
Randomization	All plant material was randomized spatially within growth facilities (greenhouse).
Blinding	Independent rescoring of chiasma frequency in a set of meiocytes gave similar results.

Reporting for specific materials, systems and methods

We require information from authors about some types of materials, experimental systems and methods used in many studies. Here, indicate whether each material, system or method listed is relevant to your study. If you are not sure if a list item applies to your research, read the appropriate section before selecting a response.

Materials & experimental systems

n/a	Involved in the study	
[x]	[ ]	Antibodies
[x]	[ ]	Eukaryotic cell lines
[x]	[ ]	Palaeontology and archaeology
[x]	[ ]	Animals and other organisms
[x]	[ ]	Human research participants
[x]	[ ]	Clinical data
[x]	[ ]	Dual use research of concern

Methods

n/a	Involved in the study	
[x]	[ ]	ChIP-seq
[x]	[ ]	Flow cytometry
[x]	[ ]	MRI-based neuroimaging
