# Supplementary material for: Ph2 encodes the mismatch repair protein MSH7-3D that inhibits wheat homoeologous recombination
Source: Nat Commun. 2021 Feb 5;12:803. doi: 10.1038/s41467-021-21127-1 (PMC7865012; doi:10.1038/s41467-021-21127-1)
Supplement: Supplementary file 4 — Description of Additional Supplementary Files [file 41467_2021_21127_MOESM4_ESM.pdf]

Description of Additional Supplementary Files

Supplementary Data 1: Genetic sequences of TaMSH7-3D from ph2b mutant and TaMSH7-3A from Cadenza.
