# Supplementary material for: Ph2 encodes the mismatch repair protein MSH7-3D that inhibits wheat homoeologous recombination
Source: Nat Commun. 2021 Feb 5;12:803. doi: 10.1038/s41467-021-21127-1 (PMC7865012; doi:10.1038/s41467-021-21127-1)
Supplement: Supplementary file 5 — Supplementary Data 1 [file 41467_2021_21127_MOESM5_ESM.docx]

Genomic sequences of *TaMSH7-3D* from *ph2b* mutant and *TaMSH7-3A* from Cadenza.

Intron start and end are highlighted in light blue and pink, respectively. ATG start codons are highlighted in yellow, mutations in red. The deletion in comparison to the TaMSH7-3A wildtype is indicated by dashes.

>ph2b-mutant [organism=*Triticum aestivum*], Chinese Spring, genomic sequence

GCACGCAGGGCGCGAGACGCGAGACGGCGGGGAAAGCGGAAAACCCCCACCCATTTCCCCTCTCCACCGCGCTCGCATTTCCCGCTCGGAAATTCCCCCAAATAATTTCATCTCCCTCCCATTCCCCTCTCCGCTCCCCACCTCCCAGAGAACCCCGCTCCTCCCGCCCGGACGGAGCCACCTCGAGTCGAATCCCGGCCACCGCG**ATG**CAGCCGCGGCGGCAGCAGCAGTCCATCCGCTCCTTCCTCCACCCGCGCCCGAGCCCGGCGCAGGAGGCGTCCGGCGCCGGCACGACCCCCGAGAGGCCCCCGCGGCCCCCCGCCGCGTCGTCGGTCGACGGCATCATGGAGCGGCTCGTGCGTCCGCCGTCGCAGGGGAGGTACGCGACCAATGCAGCTCCCGCCCGTCCTAGGGTTTTGTCTGGGTTTGGGCGTAGTTTGATTGGGCCAGCTGCCGCAGTATCTAGCTAACGGGTCCGCGCGCGCATTCGCTTAGGTGGTTGCCGCTTGACAGTGAATGCCGATTCGGTCGATTTATCGTTGGCTTGGTCTCCGCGTGTATGCTGCTGGAATTGCTTGAACCCATATTCTGGCGTGGCTTCGTTTGCTGCGGGTAGCGCTATTGTGTGGCCAGTAGCAGCAGGGATGAGGTCAATGCGAGTTTGCAGTTACGCTGTCTTTTGACGGGCTTAGCTCCTGACCGCGCTTGGTGTTTGGCCACTGCATGTGTTGTGCCTGATAGTTTACGGGCCGCATTGTCATGTTCAGTTAGTCAGTTTGTTTTTGCTATTGCGTTGTCCTCATCAGTCGCCATGCTTGCTGTTAAATCCGTTTCTCTTAACTCCAATTTGCTTACTGCCCGTGTCTCTTGGCTGTTGGATGGTGTTTTATGTGGTCTATCTGTGTTTCGATTCACATGTTTGTAGTTTTTGAGCATGAATAGATGATCCACCATAATTTTACGAACCAAAGTAAACTTCAGGTTGGAAGCTGTTCCATACAATCGAAAGGCAGAATCTGGGCTAATACATATTGTCAGTGATTGCAGATTCGTAATATGCATGTGGTTCATTCCATCTACATTGCAACTAGCATTAAGTTCATTGGATTTTGGGTCCAGTGTTTAACTGTGCTAATTGTCCTTCACTTTAGTGCAAGCTTGGATTGCTGTAATGGACTATGGATTATTTGGGTTGGATGCATTTGTTTCTGTGTTATTGGGTGTATGACAGGTGTATGTTATTGCCTTCTCGGTCCCTAAAGTGTCTTGTTTGTTGGTTTGCATCGGCAGATTTTCCGCTTACTTTGGAAAACACTTTCTTGTTTTTTTTTGCTGTGCCATCATGGTGATGCTCTCTGTGTTTGTTGCAGAAACAAAGATGCCACTCAGATTAGAAATGCTGAGAGAGCCTTACCTGGTAAAAATGAAGATACCTCAAATGAGCAGCCAAGTGCATCATTCCCTGTACGGTACAACGGCAAATATAGCAGAGGAACTGTGTTATTTGCAGAACATAGCACAGATACCACTCCGCCACAGGAGCCATTGAAGTTCTCTGCAAGGTCTTCCACAGATGAATTTGTTAGAGCAAGCACGCTGTTCCCTGAAATTGGTTCAGATCAAACTCTGCTTCAGGAGTGTCCGAAGAAGTTATCCTCAGAGTGCCCCAGCAACCAGTACGTTCAAGCTAATTCAGTGTTTGAAGCATTTGATGTACAAACTCCATCCCAGGATCCGTTAAAGAGAATCTTTTCTGGGCCTTTTCATGGAGCAGATACACCTTTATCAGAGTATCGTTCATATCCAATTCCTTTGCAGCATCCATCGAAAAAGTTGTCATCGGGCTCTTCTAGTGGTGAATACCTTAGAGCAGTGACACCGCTTGGACTTGATTCGAATGATACTCCTACAGCGAAACACTCAAAGAAGCTATTCTCTGGGTCTTCAGACCATTCATACATTAAAGCAACTAATTTGTTTCCGGAATTTGATTCAAATGGAACTCCGCTGCAGAACCACTCCAATAAGTTCTCAGTATCTATGAACAGTAAGAATATTGGAGCACCTGCTATGAACAGTAAGAATATTGGAGCACCTGCTACACTGTTTCCGGAACTTGATTCTGTTCTTCTGAAACCAGAAACTCCAGTGACACAAGCAGTGGCTCCTCGTGGGAAGAGAGTTCAACAGGATCAATGCATGACTGCCAATAACTGTCAGTCTCCTTTGTGGGGTTCAAATAAGAAGGTGAAATCAGCTCATTGTTCTCCAGCTGGGAAAATGGTTCATGATGAAATGGCTGAAAGTGCACGTAGTAAATTTGAATGGCTGAATCCTTTGAATATCAGGGATGCAAATAAAAGGCGGCCAGATGACCCACTTTATGACAAGAGAACTCTTTTTATTCCACCTGATGCACTGAGAAAGATGTCAACATCTCAAAAGCAATACTGGACTATTAAGTGTAAATATATGGATGTTCTCCTCTTCTTCAAAGTGGTAAATTCCATCATCATTGACTCATACACTGCATATATGCAGTATACTATCTGTAAGATTATCACGTTAAAGATTACTGCAATGCTAACCAGTATCTCTCATACCTTGGTGTCATACTGAGCTATGGCCTAACCCTATAATTATGAACTAGATGCATTTTTCTCATGGGACTACTGCACAATTCTTTGTTTACTTCTCATACTTAAACAGTTCTAACTCAATTACTCTATGCATCAAAAGTTAGACATTTATGAATGATGTTTGTTCTGGTTAATAATCCAAAATGTTATTTGCTTTTATTTTGGGTTCCTTCATTTGTTCTTATCCTCTCAGTCTGCTTACACCTCACAGTTTTGAATGCTGCTTTGATCCCTCTCCCAGACTCCCCTGACCTCCAAAATTATCTAAGTTGACCAGTTCTTTTCTGCTACTTGAGATTTATAACCTACAGAAGTGTTTTTCCTGTATTTGTAGGGGAAGTTTTATGAGCTCTATGAAGTAGATGCTGAGATCGGCCAAAAGGAACTTGATTGGAAAATGACTATTAGTGGGGTGGGAAAATGCCGACAGGTAACCTAAGTCATAAACCGTGACCATTCTTATTTTTCCACTGGAATGCTTGGTTCTGACGTCTGAATGTCTGCTGTGCTGACCCTGCTAATTTATACTCCCTCATGGTTGCTTGTTGGTTGAGTAATAAGTATCATTTGCTAGATCATTTGTCCTATAATATCATGTGTACGCTATATTATTTCGTATTATTTAGACAATACTCTCGGTAATGTAGTTCTATTGGCATTTTGGAGATGATTGGGTAAATTGCTTCAAGCTTTTGGCAATACACCACTTCCTCTAGGGTCTATCCGCAGCAGCTTGTGTTTTTCATATTTTGTTGGCCACTATTGTAGCATTTCATGTTTTGTTTCAACTAATGCTTTGCTCTTGAAGAAAAGGGAGTTTTATACAGCCTTTTAGTAAACACTCTTGCTGTGGTACATGAGGCTGACTTGTTCATCTTATGTGAAGGTTGGTATTTCAGAGAGTGGGATTGACAATGCTGTTGAAAAGCTTTTAGCTCGGGGGTAAACTTCTTAGTCTCCTTATGGTTTGCTCATTTTCAGCATTTTGATCTTAAAATTATCTTGAACACCAGCAAGTTCATGCACAGTTCTTCTGGTTGGTATTGATAGGCCATATGTTTGTTTCTATCGGTATTGTTGTTGATTATAATATGAGAAATGCACTTAAAATTGATTCCTTATTGGTTTCTGTGCCAGTGTTGTTGTGAAAAAAACTATATCTGTTCAGCTGCAAAATGCAATTAGTTTCCTTCTGAATGCTATATTTGATTACTCCTCCATTTTTGTACGCAAGGCCACTTTGGTTTTTCGTGTCAAACTTTGACTTATAATTTTGGTCAACAAAATACGGGTTATATGTCATAAAAAATGTATCGGAAAGTTCTTTCCAATGTGTATACATTGACATACTTTTTGTGGCATATAACTGACTTTTTGTTGGTAAAACTAATGGTCAAAATTAGACATAAAAAAAATGAAGTGGCCTTGTATACTGAAATGTATATGATATGATTTGTGATGTGACACATCCATTATAATGCTGGATATAAATTACTTCTTGCTCTACTTTTATTTCTTTTGGTGGTTTGACTGCTCATCTATCTAGCATTTCCCTCCAGATATAAAGTTGGAAGGATAGAACAAATGGAATCTGCAGCACAGGCGAAAGCTAGAGGACCAAATTCAATAAGTTTTCCACTTCCATTCTTTTTTGGATGCCCTTTCAGAATGTGTGTAGTCAACACTTTGAAACTTTGACCACTAATATATTTATTCCCTCCGTTCCTAAATATAAGCCCTTTAGAGATTCCAATATGGACTACATACGGAGCAAAATGAGTGAATCCACACACTAAAATATGTCTATATACATCCGCATGTAGTCTATATTGAAATCTCTAAAAAGTCTTGTATTTAGATTTTAGAAACGGAGGGAGTATATACTAAATAGTATTTTGATTAATCTCGTGCAAATGTTACTACTAGATTGTCATAAAAAAACTTAGAGGTCAGTACAGTTTTAAGAACTTACATTGACATGTGACTGCAAAACTAATGGTGAAGCACACACATTAGACACTCTCTTGGTCTAAAACATGTAGTAGAATTGATCAAGGTAGTAGGTACATAACCTGTGGTCTGTGCAGCAGCACTTGAATGCATTGCATATATTAATATGTACCCTGGCATCTCCATTGACGGATGCATAAAGCTTGTCCTTTCAAGTTACAAGAGATTGACTGTATATAAGTGTGACATTTTTTTTCGTACTTCTGCAAGGTTATTGAAAGAAAGTTAGCTCATGTATCCACACCGTCAACTGCAGCTGACAGCAACATAGGGCCTGATGCTGTTCATCTTCTTGCATTGAAAGAGGTTTGCCTTTTTTAACAAAGCAATACATTGCATTTGGCAATATGATTTGGTATAATGTTTTGTATGTTCATCTGACGCACATGAGAGGACACAATACTACCAGAAAAGCTAACCATCTGATAATAGAAAGAACACTTTCCTCAAAAGACAGGAAAGAACTACAAACTCGTGGTCTTTGGACACAATGACCAAATAAATTTACATAGAAACATAGTGATAAATCTAAATCTGGTTGATCACTGTGTATAAACATGTAAAATATTGTGGCAATAATGTAGAAATATATGTAACTTGCAGAAGACAATCTTGATACTTAATTCAGCTATTGTTTTAGTAAAATGATATTTTCAGTGTCCCTGTGAAACATGCAGTGTTATGTATCCTAGTTTTTACGAGTCAGTTTTGTCCTGCAATTTGTTCACAAATTCTGTAATTCATCTTGAATTAGTTTTATCAACATTTTTCATAGGTGATGTTGACCTAATGTAGCCTGTCCTCTTTTTTGTTGTTGGAATTTTCAGGTTACTCTAGCTTCTAATGGTTCTCGGGTCTACGGATTTGCTTTTCTAGATTATGCTGCACTTAAAATCTGGGTTGGTTCACTTCAAGATGATGATTCTTCTGCAGCTTTGGGGGCTTTGCTGGTGCAGGTAGGCTCTTTGCTGTAGCAATTTATTTGCTTTGGTTCAACAGATCAGAAATGATCATTCATCAGTATGCTTTTTCAGGTTTCCCCGAGGGAGATAATCTATGAATCCTCAGGTCAGTACATCTTTTATCTTGTAATCTCGATGTATTAAGAACTAATATTTGTTTTCTTTTGTCAGGCCTCTCAAGAGAAAGTCGTAAATCAATGATAAAATATGCCTCAGCAGGTAAAATGTTGTAATCTTTTGCTCTTTTAGTTTCTATTCTCATCTGGTTTCTTTGTTAAACATCTTTCTTCTAACTGCAGGCTCTGTGAAAATGCAACTGACCCCACTACCTGGGACAGATTTCTCTGATGCCTCACAAATTCAAATGCTAGTACATTCTAAAGGATACTTTAAAGCATCAACAGATTCTTGGCTATCTGCATTGGATTATTCAGTGAATCAAGATGCAGTTATCTGTGCACTTGGTGGACTTATTGGTCATTTGACTAGACTTATGGTATACTTATGTTAATCCAACTCTCTCTCTCTCTCTCTCTCCGAGTGCCTCTGTTGCAACATTTTCCTTCTCAGCAACACTTGCAAATTGCTTCAGAACACTGTCATTAGACATCACTTTCCAGTTTCCTAGATACAACTAATTAGTATGTAGTTTCTTTAATATATAAATTTTTCTCATTTTCTATAATGACCATATCAACTTCACAAATTATATGTGCAAAGGTGTTCTAGTTTTAAACTAGTATAATGTCAAAAAATTGTTTGCACAAATCATAGCATAAATGATGTGCTTGCCTTTCTTTTTTTATTTGCTTGGGAAACGATTGTTTTCATAAGACTGCCTGTTCACGTTCTGATAAGCTACAATCTGGAAACAGCTAGACGATGCTCTAAAAAATGGGGAAGTCTTACCTTACAATGTGTACCAAACTTGTTTAAGGATGGATGGTCAGACTCTTGTGAACCTGGAGATTTTCGGCAATAACTTTGATGGTGGCTCATCAGGTAGGAGTTTGCCTTCTACCTATTTTATTTTAGCAGCCTGCCGTGACAATTTCCTAATATATCCAAACATAAAAACACTGATAAACTTTGTATTATATTTACTTTGTTTTAGGTACTCTGTACAAGCACCTCAATCACTGCATAACCGCATCTGGTAAGCGGCTTTTAAGAAGATGGATATGCCATCCACTAAAAGATGTCGATGCTATAAATAGAAGGCTTGATATTGTTGAGGGTTTCATTCAACATTGTGGGGTAGGCTCTGTTACACTTGAGCATCTCCGGAAAATTCCTGACCTTGAGAGGTTACTTGGGCGAGTCAGATCTACTGTTGGGCTAACATCTGCTGTCCTGTTGCCTTTTGTTGGTGAAAAGATATTAAAGAGGCGGGTTAGTGGTTGTTCTTTCTTTTTCATTCCCAGTCCTAAGCTTATCGTAGCTTAAAGTTCTCTTTTGTATGGATTAGATTCAATCAATGAGATGCCCATTTTTGCAAATTATGAAGTTTGCCTTTCTTGTATCTACACTACTGCTAATGACATGCAAATTTTCTGCATTTTCCAGATTAAAACGTTTTGCATGCTTATCAAGGGCCTCCGGGTTGCAATTGACTTATTAAGTGCCTTGCGTAGAGAGGACCATGGCATTCCAGCGCTGTCAAAATCAGTTGATATTCCAACCCTGAGTTCTCTTGATGAATCAGTCCATCAGTTTGAAGAGGCTATACGCATTGACTTTGAACAGTACCAGGTATGAATATGCTGGGAATTTCTTCGGACGCTATCTATCTAAGAAGCGGTTATTTTTAGTATTTGCTGTCTGTGTTTTGGACTTCCACAGTACTGATTGTGTGTTTTGAGTTTTTGACAGGATCATGATATCAAAGACCATGATGCTACCACCTTGGCTAATTTAGTGGAACATTTTGTTGGAAAAGCTACCGAATGGTCTTTGGTAATCAATGCCATCAGCACTGTTGATGTCCTTAGGTCCTTTGCAGCAATGGCATTGTCATCATTTGGCACCATGTGCAGACCACGTATTCTGTTGAAAGACAAATCGCCTATACTTCGGATGAAGGGTCTATGGCATCCATATGCTTTTGCAGAAAGTGGAACTGGGCTTGTGCCAAACGATTTGTCTCTTGGCCAGGATTTATTGGGTCATAATCGCTTTGCATTGTTGTTGACTGGTCCAAATATGGGAGGAAAATCTACAATAATGCGCGCCACCTGCTTGGCTATCGTGCTTGCCCAGGTATGTATAAGTAAATCTGGTTATACCCTTTTTCTTGTTAACTCCTGGGCTCCCTATTTGGATGTATAAATGGAATTTCTTGTCTGCAGCTTGGCTGTTATGTCCCCTGCATATCATGTGAATTGACCCTTGCAGACTCCATCTTTACACGGCTAGGCGCAACGGATCGGATTATGTCTGGAGAAAGTAAGTAATCAGTGTTGCAAATCCTATTGCTTTTGGCGCAAAACAGGAGTTAAAAACGGATAAGGTCTTTATATGGGTTGTCCTACAACTGGATCTGGTCAGACATGGGTTTATTTTTTATTGTTCTGCATAAATAATATATTCTGACACTGGTTCCCAATAAAATGATACTTGTATAGTCAAATATGCATTTGCACATAAATTATATAGCTATTTTTACATGGTATATCTGTTGAGTAAATCCATGGGTTGTCAATATTAACTTCTAAAGCACTTGTCAATTTTCTTTTGGCCGTACCATGAATCATGAACAGGGTATGAGATGACAATGACCCTAACTTGGATTGTTACAACATTATTTGCGGTCTTGCAGGTACTTTTCTTGTCGAATGTAGTGAGACTGCATCTGTTCTTCAGAATGCAACTGAGGATTCTCTTGTCTTGCTTGATGAACTTGGCAGAGGAACTAGCACATTTGATGGATACGCGATTGCATATGCAGTAAGTCTTCCTTCAATCCATTACATAACCATGCTTGCCATACCCTTCAGTATGCCTTTTTCATGGTAGATGCTGTCGCCGTGTCTTTTAAGGTCATTGTCCGTATCAGTAAGCTACATAGCTTCAAGAACGATCTTATATTATGGGACGGAAGGAGTAGTTTTGCATATCAGCCATTGATAGGACAGTCAGAAAACCGATCAATTTCGTACGTTCAACATGGCATATCAGCCATTGCCGTGTGCTTTCTCATTGTTTTCACATCGTATCCATTTTCTGACAAACAATTTCGTGCTTCTGATGACTGTATTTTGCCTCAGGTATTCCGCCACCTGGTGGAACAGGTGCGATGCCGTCTGCTCTTTGCCACCCACTACCACCCTCTCACCAAGGAGTTCGCCTCCCACCCCCACGTGAGCCTCCAGCACATGGCCTGCATGCTGAGGCCAAGGAGCGGCGGCAACGGCGAGATGGAGCTCACCTTCCTCTACCGTCTTGTGTCAGGCGCCTCTCCGGAGAGCTACGGCCTGCAGGTCGCCACGATGGCGGGGATCCCAAAGTCCATAGTGGAGAAGGCGGCGGTCGCGGGCGAGATGATGAAGTCGAGGATCGCAGGGAACTTCAGGTCGAGCGAAGGGCGAGCGGAGTTCTCCACCCTCCACGAGGACTGGCTGCAGACGATCCTGGCGATCGGCGGCGTCAAGGACGCGCACCTGGACGAGGACACCATGGACACGATGTTCTGCGTCGCCCAGGAGCTCAAGTCTCATTTCAGGAAAGGAGGAAGCTGAGCGCTGAGAAGTCGCCACCGGTAATTATGCGTGGCACCATTAGATGTAGGTAGTCTGAAGGAGGAAGATGAGCACCGAGAAAGTCGCCGCTCACCATTAATCATCAGTGTTTTAATCCGTCCCAGTCGACGGCTTTGTATATATTTACCTCGCGTTTGT

>*TaMSH7-3A*_deletion_mutant [organism=*Triticum aestivum*], Cadenza, genomic sequence

CCACCGCGCTCGCATTTCCCGCTCGGAAATTCCCCCAAATAATTTCATCTCCCTCCCATTCCCCTCTCCGCTACCACCTCCCAGAGAACCCCGCTCCTCCCGCCCGGACGGAGCCACCTCGAATCGAATCCCGGCCACCGCG**ATG**CAGCCGCGGCGGCAGCAGCAGCAGTCCATCCTCCCCTTCCTCCACCCGCGCCAGGGCCCGGCGCAGGAGGCGCCCGGCGCCGGCAGGACCCCCGAGAGGCCCCCGCGCCCCCCCGCCGCGTCGTCGGTCGACGGCATCATGGAGCGTCTCGTGCGGCCGCCGTCGCAGGGGAGGTACGCGACCAATGCAGCTCCCGCCGTCCTAGGGTTTTGTCCGGGTTTGGGTGCCGTTTGATTGGGCTAGCTGCCGCGGTATCTGGCTAACGGGTCCGCGCGCGCATTCGCTTAGGTGGTTGCCGCTTGATAGTGGATGCCGATTCGGCCGATTTATCGTTCCATTGGTCTCCACATGTATTCTGCTGGAATTGCTTGAACCCCAAATTCTGGCGTGGCTTCATTTGTTGCGGGTAGCGCTATTGTGTGGCCGGTAGCAGCAGGGATGAGATCGATGCGAGTTTGCAGTTGCGCTGTCTTTTGACGGGCTTAGCTGCTGACCGCGACTGGTGTTTGGCCACTGCATGTGTTGTGCCTGATAGTTTACGGGCCGCGTTGTCATCCTCAGTTAGTCAGTTTGTTTTTGCTATTGCGTTGTCCTCATCAGTCGCCATGCTTGCTGCTAAATCCGTTTCTCTTAACTCCAATTTGCTTACTGACCGTGTCTCCTGGCGAACCAAAGTAAACTTCAGGTTGGAAGCTGTTCCGTACAATCGAAAGGAAGAATCTGGTCTAATACACATCACCATTGATTGCAGATTCATATTTTGCATGTGGTTCACTCCATCTACATTGCAACTAGCCTTAAGCTTATTGGATTTTGGGTCCAGTGTTTAACTGTGCTAATCGTCCTTCACTTCAGTGCAAGCTTGGATTGCTGTAATGGACTATGGATTGTTTGGGTTGGATGCATTTGTTTCTGTGTTATTGGGTGTATGACAGGTGTATGTTATTGCCTTCTTGGTCCCTAAAGTTTCTTGTTTGTTTGTTTGCATCGGCAGATTTTCTGCTTATTTTGGAGAACACTTTTTTTTGCTGTGCCATCATGGTGATGCTCTCTGTGTTTGTTGCAGAAACAAAGATGCCGCTCAGATTAGAAATGCTGAGAGAGCCTTACCTGGTAGAAATGAAGATACCTCAAATGAGCAGCCAAGTGCATCATTCCCTGTACGGCACAATGGCAAATATAGCAGAGGAACTGTGTTATTTGCAGAACATAGCACGGATACCACTCCGCCACAGGAGCCATTGAAGTTCTCTGCAAGGTCTTCCACCGATGAATTTGTTAGAGCAAGCACGCTGTTCCCTGAACTTGGTTATCCAACTCTGCTTCAGGAGTGTCCGAAGAAGTTATCCTCAGAGTGCCCCAGCAACCAG**----------------------------**CATTTGATGTACAAACACCGTCCCAGGATCCGTTAAAGAGAATCTTTTCTGGGCCTTTTCATGGAGCAGATACACCTTTATCAGAGTATCGTTCATATCCAATTCCTTTGCAGCATCCATCGAAAAAGTCGTCATCGGGCTCTTCTAGTGGTGAATACCTTAGAGCAGTGACACCGCTTGGACTTGATTCGAATGATACTCCTATAGCGAAACACTCAAAGAAGCTATTCTCTGGGTCTTCAGACCATTCATACATTAAAGCAACTAATTTGTTTCCGGAATTTGATTCAAATGGAACTCCGCTGCAGAACCACTTGAATAAGTTCTCAGTATCTATGAATGGTAAGCATATTGGAGCACCTGCTACACTGTTTCCGGAACTTGATTCTGTTCTTCTGAAACCAGAAACTCCAGTGACACGAGCAGTGGCTCCTCGTGGGAAGAGAGTTCAACAGGATCAATGCATGACTGCCAATAACAGTCAGTCTCCTTTGTGGGGTTCAAATAAGAAGGTGAAATCAGCTCATTGTTCTCCAGCTGGGAAAATGGCTCATGATGAAATGGCTGAAAGTGCACGTAGTAAATTTGAATGGCTGAATCCTTTGAATATCAGGGATGCAAATAAAAGGCGGCCAGATGACCCACTTTATGACAAGAGAACTCTTTTTATTCCACCTGATGCACTGAGAAAGATGTCAACATCTCAAAAGCAATACTGGACTATTAAGTGCAAATATATGGATGTTCTCCTCTTCTTCAAAGTGGTAAATTCCATCATCATTGACTCATACACTGCATATATGCAGTATACTATCTGTAAGATTATCACGTTAAAGATTACTGCAATGTTAACTAGTATCTCTCATACCTTGGTGTCATACTGAGCTATTGCCTAACCCTATAATTATGAACTAGATGCATTTTTCTCATGGGACTACCGCACAATTCTTTGTTTACTTCTCATACTTAGACAGTTCTACTCAATTACTCCATGCATATTGACATATGCATCAGAAGTTAGACATTTATGAATGATGTTTGTTCTGGTTAATAATCCAAAATGTTATTTGCTTTTATTTTGGGTTCCTTCATTTGTTCTTATCCTCTCAGTCTGCTTACACCTCACAGTTTTGAATGCTGCTTTGATCCCTCTCCCAGACTCCCCTGACCTCCAAAATTATCTAAGTTGACCAGTTATTTTCTGCTACTTGAGATATATAACCTACAGAAGTGTTTTTCCTGTATGTGTAGGGGAAGTTTTATGAGCTCTATGAAGTAGATGCTGAGATCGGCCAAAAGGAACTTGATTGGAAAATGACTATTAGTGGGGTTGGAAAATGCCGACAGGTAACTTAAGTCACAAACTGTGACCATTCTTATTTTGCCACTGGAATGCTTGGTTCTGATGTCTGAATGTCTGCTGTGCTGACCCTGCTAATATATACTCCCTCATGGTTGCTTGTTGGTTGAGTAATAAGTATCATTTGCTAGATCATTTGTCCTATAATATCATGTGTACACTATATTATTTTGTATTATTTAGACAATACTCTCGGTAATGTAGTTCTATTGGCATTTTGGAGATGATTGGGTACATTGCTTCAAGCTTTTGGCAATACACCACTTCCTCCAGGGTCTATCCGCAGCAGCTTGTCTTTTTCATATTTTGTTGGCCACTATAGTAGCATTTCATGTTCTGTTTCAACTAATGCTTTTGTATTAAACACTCTTGAAGAAAAGGGAGTTTTATACAGCCTTTTAGTAAACATTCTCGCTGTGGTACATGAGGCTGACTTGTTCATCTTATGTGAAGGTTGGTATTTCAGAGAGTGGGATTGACGATGCTGTTGAAAAGCTTTTAGCTCGGGGGTAAACTTCTTAGTCTCCTTATGCTTTGCTCATTTTCAGCATTTTGATCTTAAAATTATCTTGAACATCAGCAAGTTCATGCACAGTTCTTCCGGTTGGTATTGATAGGCCATATGTTTGTTTCTATCGGTATTGTTGTTGATTATAATATGAGAAATGCACTCAAGATTGATTCCTTATTGGTTTCTGTGCCAGTGTCGTTGTGAAAAAAAAACTATATCTGTTCAGCTGCAAAATGCATTTAGTTCCATCTGAACGCTATATTTGAGTACTCCCTCCATTTCTGTATGCAAGGCCACTTTGGTTTTTCGTGTCAAACTTTGACTTATAATTTTGGTCAACAAAATACGGGTTATGTCATAAAAAATATATCATTGGAAAGTTCTTTCCAATGTGTATACATTGACATACTTTTTGTGGCATATAACTCACTTTTTGTTGGTAAATTTAATGGTCAAAATTAGACATAAAAAAATATGAAGTGGCCTTGTATACTGAAATGTATATGATATGATTTGTGATGTGACACATCCATTATAATACTGGATATAAATTACTTCTTGCTCTACTTTTATTTCTTTTGGTGGTTTGACTGCTGATCAATCTAGCATTTCCCTCCAGATATAAAGTTGGAAGGATAGAACAAATGGAATCTGCAGCACAGGCGAAATCTAGAGGACCAAATTCAGTAAGTTTTCCACTTCCATTCTTTTTTGGATGCCCTTTCAGAATGTGTGTAGTCAACACTTTGAAACTTTGACCACTAATATATTATTCCCTCCGTTCCTAAATATAAGCCCTTTAGAGATTCCAATGTGGACTACATACGGAGCAACATGAGTGAATCCACACACTAAAATACGTCTATATACATCCGTATGTAGTCTATATTGAAATCTCTAAAAAGTCATATATTTAGATTTTAGAAACGGAGGTATATACGCAATAGTATTTTGATTAATCTCGTGTAAATGTTATTACTAGATTGTCATAAAAAAGCTTAGAGGTCAGTACAGTTTTAAGAACTTACATTGACATGTGACTGCAAAACTAATGGTGAAGCACACACATTAGACACTCTCATGGTCTAAAACATGTAGTAGAATTGATCAAGGTAGTAGTTACATAACCTGTGGTCTGTGCAGCAGCACTTGAATGCATATATTACTATGTACCCTGGCATCTCCATTGACGGATGCACGTCCTTTCAAGTTACAAGAGATTGACTGTATATAAGTGTGACTTTTTTTTTGTACTCCTGCAAGGTTATCGAAAGAAAGTTAGCTCATGTATCCACACCGTCAACTGCAGCTGACAGCAACATAGGGCCTGATGCTGTTCATCTTCTTGCATTGAAAGAGGTTTGCCTTTTTTAACAAAGCAATACATTGCATTTGGCAATATGATTTGGTATAATGTTTTGTATGTTCATCTGACGCACATGAGAAGACACAATACTACCACAAAAGCTAACCATCTGATAATAGAAAGAACACTATTTCCTCAAAAGACAGGAAAGAACTACAAACTCGTGGTCTTTGGACACAATGACCAAATAAATTTACATAGAAACATAGTGATAAATCTAAGTCTAGTTGATCACTGTGTATAAACATGTAAAATATTGTGGCAGTAGAAATATATGTAACTTGCAGAAGACAATCTTGATACTTAATTCAGCTATTGTTTTAGTAAAATGATATTTTCACTGTCCCTGTGAAACATGCAGTGTTATGTATCCTAGATTTTACGAGTCAGTTTTGTCCTGCAATTTGTTCACAAATTCTGTAATTCATCTTGAATTACTTTTATCAACATTTTTCATAGGTGATGTTGACCTAATGTAGCCAGTCCTTTTTTTTTTGGGAATTTTCAGGTTACTCTAGCTTCTAATGGTTCTCGGGTCTACGGATTTGCTTTTCTAGATTATGCTGCACTTAAAATCTGGGTTGGTTCACTTCAAGATGATGATTCGTCTGCAGCTTTGGGGGCTTTGCTGGTGCAGGTAGGCGCTTTGCTGTAGCAATTTATTTGCTTTGGTTCAACAGATCAGAAATGATCATTCATCAGCATGCTTTTTCAGGTTTCCCCGAGGGAGATAATCTATGAATCCTCAGGTCAGTACATCTTTTATCTTGTAATCTCGATGTATTAAGAACTAATATTTGTTTTCTTTTGCCAGGCCTCTCAAGAGAAAGTCGTAAATCAATGATAAAATATGCCTCAGCAGGTAAAATGTTGTAATCTTTTGCTCTTTTAGTTTCTATTTTCACCTGGTTTCTTTGTTAAACATCTTTCTTCTAACTGCAGGCTCTGTGAAAATGCAACTGACCCCACTACCTGGGACAGGTTTCTCTGATGCCTCACAAATTCAAATGCTAGTACATTCTAAAGGATACTTTAAAGCATCAACAGATTCTTGGTTATCTGCATTGGATTATTCAGTGAATCGAGATGCAGTTATCTTTGCACTTGGTGGACTTATTGGTCATTTGACTAGACTTATGGTATACTTATCTTAATCCAACTCTCTCTCTCCGAGTGCCTCTGTTGCAACATTTTCCTTTTCAGCAACACTTCAAATTGCTTCAGAACCTATGTTTTCAAGGCGTCCAGGCGCTTTAAGGCGCTGGGGGGGCGCCTCAACGCCTAGGCGCTCAAAGCGTGAGGCGAGGCGACGCCTTAGACACTTATTATTAGGCGCTAGGGGTACTAATATGGCAGCCATGAGTGGACATGTAACTGATTCATACCTTGAATTTCCTGGTTGTTGAGCCCACATAGCATCCATTCCTCCCGTCTTGTTTCCCATTTCTGCCTTCCACAATAGAAATCTGCATAAAGATGAACATATATCATAGTATATGGTTAAAACTAGACTTAATTAATAGATCACCATTGGTAAAAAACAGAGAGTAAATGAGGGGGAGGCAAAGCAGTGAGGCACAAGCAGCAGCACCATCAGAAAACATGGGCAGGTGAGGGGAAAAGAGAGGCAGAGAGGAGCCGTACCTTGGGAGAGGGCTGCCGGCGGCCGAGGAGCTTGGCCGGAGAGGGGGATGGCCGGCGGAGATGGATCTTGGCCGGAGAGGGGAAGGGCCTGCGGAGGAGGAGCTTGGCCGGACAGGGGGAGGCCGGCGGAGGAGGAACGGATCTGGGCCGGCGGCGTGAGAGACTGAGAGGAGAGAGCTCCTCTCTCTTTTCCCCTCGATTTGGAGTCACAGGTGTGTTCCCCTCTCTCTCCTGCCTCTGTTTACCCCCTCTCTCCCGCCCAATTTCTCTTCCCTCCCGCCAATTCCTCTTTCTGGCGCGCGCCAGCTGCTGGTTCCCGCTCAACCTGCCCGCGCATGTCCAGGGCGTCCAAAATCAAGCAAAGCGGTGCCTTGGACGCCTAGGCGACGCCTTGGACGCCTCAACAGCACAAGGCGGACGCCTTAGACAAAAAACTAAAGCGACGGCGACGCCTTGACGTTCGCGAGCGCTCTGACGCCTAGGCGTCGCCTAGGCGACGCCTAGGCGACGCCTTGAAAACATAGTTCAGAACACTGTCATTAGACATCACTTTCCTGTTTCCTAGATACAACTAATTAGTACTCCCTCCATTCCACAATGTAGTGCTTCCTCTATCCACGTGCTCCAACTTTGACCGTAAATTTAACTACCAAGACCGATTGCGGCGGGAGCAAAAATTATATCAGTGAATTCGTATTCGAAAAAAGTTTTCAATTATATAATTTTTTTTCTCCCGCCGCAATTGGTCTCATTGGTTAAATTTATGGTCAAAGTTGGACCTCGGGAAGCGCGGGCGCACTATATTTTGGAATGGAGGGAGTATGTAATTTCTTTAATATATAATTTTTTTCTCATTTTCTATGCTGACCATATCAACTTCACAAGTTATATGTGCAAAGGTGTTCTAGTTCTAAACTAGTATAATGTCAAAAAATTGTTTGCACAAATCATAGAGTAAATGATGTGCTTGCCTTTCTTTTTTTATTTGCTTGGGAAACGATTGTTTTCATAAGACTGCCTGTTTACGTTCTGATAAGCTACAATCTGGAAACAGCTAGACGATGCTCTAAAAAATGGGGAAGTCTTACCTTACAATGTGTACCAAACTTGTTTAAGGATGGATGGTCAGACTCTTGTGAACCTGGAGATTTTCGGCAATAACTTTGATGGTGGCTCATCAGGTAGGAGTTTGCCTTCTACCTATTTTCTTCTCTACTTATTTTTGCAGCCTGCTGTGACAATTTCCTAACATATCCAAACATAAAAACACAGATAAACTTTGTATTATATTTACTTTGTTTTAGGTACTCTGTACAAGCACCTCAATCACTGCATAACCGCATCTGGTAAGCGGCTTTTAAGAAGATGGATATGCCATCCACTAAAAGATGTCGATGCTATAAATAGAAGGCTTGATGTTGTTGAGGGTTTCATCCAGCATTGTGGGGTAGGCTCTATTACACTTTATTATCTCCGGAAAATTCCTGACCTTGAGAGGTTACTTGGGCGAATCAGATCTACTGTTGGGCTAACATCTGCTGTCCTGTTGCCTTTTGTTGGTGAAAAGATATTAAAGAGGCGGGTTAGTGGTTGTTCTTTCTTTTTCATTCCCAGTCCTAAGCTTATCGTAGCTTAAAGTTCTCTTTTGTATGGATTAGATTCAATCAATGAGATGCCCATTTTTGCAAATTATGAAGTTTGCCTTTCTTGTATCTACACTACTGCTAATGACATGCAAATTTTCTGCATTTTCCAGATTAAAACGTTTGGCATGCTTATCAAGGGCCTCCGGGTTGGAATTGACTTATTAAGTGCCTTGCGTAGAGATGACCATGGCATCCCAGCGCTGTCAAAATCAGTTGATATTCCAACCCTGAGTTCTCTTGATGAATTAGTTCATCAGTTTGAAGAGGATATACACAATGACTTTGAACAGTACCAGGTATGAATATGCTGGGGATTTCTTCGGACGCTATCTATCTAAGAAGCGGTTATTTCTTTTTCTAGTATTTGCTGTCTGTGTTTTGGACTTCCACAGTACTGATTGTGTGTTTTGAGTTTTTGACAGGATCATGATATCAAAGACGGTGATGCTACCACCTTGGCTAATTTAGTGGAACATTTTGTTGGAAAAGCTACCGAATGGTCTTTGGTAATCAATGCCATCAGCACTGTTGATGTCCTTAGGTCCTTTGCAGCAATGGCATTGTCATCATTTGGCACCATGTGCAGACCATGTATTCTGTTGAAAGACAAATCGCCTATACTTCGGATGAAGGGTCTATGGCATCCATATGCTTTTGCAGAAAGTGGAACTGGGCTTGTACCAAACGATTTGTCTCTTGGCCAGGATTTATCGGGTCATAATCGCTTTGCATTGTTGTTGACTGGTCCAAATATGGGAGGAAAATCTACAATAATGCGCGCTACCTGCTTGGCTATCGTGCTTGCCCAGGTATGTATAAATAAATCTGGTTATACCCTTTTGCTTGTTAACTCCTGGGCTCCCTATTTGGATGTATAAATGGAATTTCTTGTCTGCAGCTTGGCTGTTATGTCCCCTGCATATCATGTGAATTGACCCTTGCAGACTCCATCTTTACACGGCTAGGCGCAACGGATCGGATTATGTCTGGAGAAAGTAAGTAATCAGTGTTGCAAATCCTATTGCTTTTGGCGCAAAACAGGAGTTAAAAATGGATAAGGTCTTTATATGGGTTGTCCTACAACTGGATCTGGTCAGACATGGGTTTATTTTTTATTGTTCTGCATAAATAATATATTCTGACACTGGTTCCCAATAAAATGATACTTGTATAGTCAAATATACATTTGTAAATTATATAGCTATTTTTACATGGTATATCTGTTGAGTAAATCCATGGGTTCTCAATATTAACTTCTAAAGCACTTGTCAATTTTCTTTTGGCCGTACCATGAATCATGAGGTATGAGATGACAATGACCCTAACTTGGATTGTTACAACATTATTTGCGGTCTTGCAGGTACTTTTCTTGTCGAATGTAGTGAGACTGCATCTGTTCTTCAGAATGCAACTGAGGATTCTCTTGTCTTGCTTGATGAACTTGGCAGAGGAACTAGCACATTTGATGGATATGCGATTGCATATGCTGTAAGTCTTCCTTCAATCCATTACATCACCATGCTTGCCATACCCTTCAGTATGCCTTTTTCATGGTAGATGCTACATAGCTTCAAGAACGATCTTATATTATGGGACGGAAGGAATAGTTTTGCATATCAGCCATTGATAGGACAGTCAGAAAACCGATCAATTTCGTACGTTCAACATGGCATATCAGCCATTACCGTGTGCTTTCTCATTGTTTTCACATCGTATCCATTTTCTGACAAACAATTTCGTGCTTCTGATGACTGTATTTTGCCTCAGGTATTCCGGCACCTGGTGGAACAGGTGCGATGCCGCCTGCTCTTCGCCACACACTACCACCCTCTCACCAAGGAGTTCGCCTCCCACCCCCACGTGAGCCTCCAGCACATGGCCTGCATGCTGAGGCCAAGGAGCGGCGGCAACGGCGAGATGGAGCTCACCTTCCTCTACCGGCTCGCGTCAGGCGCCTCCCCGGAGAGCTACGGCCTGCAGGTCGCCACGATGGCGGGGATCCCAAAGTCCATAGTGGAGAAGGCGGCGGTCGCGGGCGAGATGATGAAGTCGAGGATCGCGGGGAACTTCAGGTCGAGCGAAGGGCGAGCGGAGTTCTCCACCCTCCACGAGGACTGGCTGCAGACGATCCTGGCGATCGGCGGCGTCAAGGACGCGCACCTGGACGAGGACACCATGGACACGATGTTCTGCGTCGCCCAGGAGCTCAAGTCCCATTTCAGGAAAGTAGGAAGATGAGCGCTGAGAAGAGTCGCCACCAGTAATTATGTGTGGCATATCATTAGATGTAGCTAGTCTGCAGGAAGAAGATGAGCAACGAGAAAGTCGCTGCTCACCACTAATCATCAGTGTTTTAATCCGTCCCAGTCGACGGCTTGTACATATTCACCTCGCGTTTGTCATCGCAACCGCACCTGGGCCTGAGTTCATCTGAACTGTCAAAAATCAT
