# Supplementary material for: Ph2 encodes the mismatch repair protein MSH7-3D that inhibits wheat homoeologous recombination
Source: Nat Commun. 2021 Feb 5;12:803. doi: 10.1038/s41467-021-21127-1 (PMC7865012; doi:10.1038/s41467-021-21127-1)
Supplement: Supplementary file 2 — Peer Review File [file 41467_2021_21127_MOESM2_ESM.pdf]

Reviewer #1 (Remarks to the Author):

Why Ph2 is important?
On wheat's polyploidisation, Ph1 arose to stabilise tetraploid wheat meiosis through promoting homologous pairing and suppressing homoeologous crossover, so preventing to unbalanced gametes and infertility in this polyploid. The subsequent hybridisation creating hexaploid wheat, brought in an additional locus Ph2 on its D genome, which further helped stabilise the meiosis of this hexaploid by also suppressing homoeologous crossover. Thus, these two loci contribute to stabilising bread wheat as a major global crop, making them of key importance. Moreover, the introgression of traits from wild relatives into hexaploid (bread) wheat exploiting homoeologous recombination has saved the US several billion dollars over the years. Thus, the Ph1 and Ph2 loci in wheat are of key economic importance. Although the introductory information for Gordon and EMBO meiosis meetings state that meiotic research will contribute to breeding, and unpin human health as meiosis can give rise to human aneuploidy. In terms of breeding, exploitation of homoeologous recombination in wheat breeding is still the only actually practical use of meiotic research. Therefore, it was important to identify the genes responsible for Ph1 and Ph2.

The Results-
The homoeologous crossover phenotypes are not trivial to score as they need to be scored through crossing with a wild relative and then scoring meiosis in the hybrids. Thus, unlike most plant traits, Ph2 (or Ph1) can't in practical terms be identified through segregating populations. Instead they have to be initially mapped through deletion analysis, which is a significant undertaking. After an enormous amount work presented in a series of publications, Ph1 has recently been defined to a duplicated copy of the meiotic gene ZIP4 (5B), the papers cited in this MS. The authors now present a significant amount of work as a single publication which clearly defines Ph2 to the meiotic plant gene MSH7-3D. The work required to define this gene involved generating and scoring deletions for the presence, or loss of the Ph2 phenotype, exome capture to show that MSH7 3D copy was indeed mutated in ph2 EMS (ph2b) mutant, expression profiling, exploitation and screening further MSH7 3D EMS mutants and their scoring. The results are clear-cut. The ph2b MSH7 3D ems mutant has a similar phenotype as a 121Mb deletion line ph2a when crossed wild relatives, and the ph2b mutant has a similar level as the Cadenza2006 ems mutant also when crossed to wild relatives. The cadenza2006 mutant has an early stop codon in MSH7 3D, and the authors now show that the ph2b mutant also carries a mutation in its MSH7-3D resulting in a frameshift and early stop codon. Thus, the authors have two MSH7 3D ems mutants (both producing early stop codons) which give the full homoeologous crossover phenotype. Moreover, when homologous crossover is scored, both mutants have a reduction in this crossover.

I really enjoyed reading this very nice MS.
I have some comments which I hope that the authors find useful.

The crossover effect-
The results/discussion on the scoring of the other MSH7-3D mutants ought to be de-emphasised. They only yield a partial phenotype, both in terms of both homologous crossover reduction in the mutants themselves, and the level of homoeologous crossover when crossed with wild relatives. It detracts from the clear message for somebody who may not have a detailed knowledge of this area, namely that the authors have MSH7 3D mutants with full Ph2 phenotype when compared to a mutant carrying a 121Mb deletion covering of Ph2 gene. There could be a debate about whether increasing homoeologous crossover by just over one is significant, when this study does not need to go there. The emphasis should be on the candenza2006 and ph2b results. These two MSH7 3D mutants, both have the full phenotype. If the authors are worried that they just have two mutants, they don't need to be as the chance that there is another gene mutated in the 14Mb region giving the same effect in both lines (where one line, ph2b has been grown over a number of generations) would be in the 100's of millions. Thus, in the same vain, it is unclear to me what the cross between 1114 and 2006 tells us or adds, when 1114 only has a partial phenotype? The increase in multivalents in the resulting cross in

contrast to rare multivalents (0 or 0.04) in individual mutants themselves probably suggests something else going in the background. It simply complicates. I would suggest removing.

I know what the authors are trying to say, but as written the sentence and the word 'pairing' is not correct in ‘In contrast to MMR proteins there is no ZIP4 involvement in the inhibition of pairing between homoeologous (divergent) DNA sequences.’ When there are no homologues present, related chromosomes (homoeologues) pair and synapse in wheat hybrids after the telomere bouquet with both MSH7D and ZIP4 5B present (Nat communs 2014, Chromosoma 2017 papers both cited), and the recombination machinery appears to load. Two barley homoeologues still paired in wheat itself when ZIP4 5B or MSH7 3D is present (Prieto group Frontiers 2018 study). Thus, neither MSH7 3D nor ZIP4 5B can prevent homoeologues pairing and synapsing, instead, they both prevent successful crossovers from occurring between such paired diverged chromosomes. This ought to reworded.

The mutation of MSH7 3D (or ZIP4 5B) also both lead to a similar reduction in homologous crossover. Thus in terms of crossover, both the mutation of MSH7 3D or ZIP4 5B lead to a similar reduction in homologous crossover, and an increase in homoeologous crossover. The only slight difference in crossover is that mutation of MSH7 3D induces two fold less homoeologous crossovers than ZIP4 5B. Thus, Ph2 and Ph1 in terms of crossover are essentially very similar. ZIP4 is a tetratricopepticide repeat protein, which basically can form folds for binding proteins and assembling protein complexes, akin to a docking station. Given the similarity in their crossover effect , it raises the question as to whether MSH7-3D is part of one of these complexes?

Pairing
The difference between MSH7-3D and ZIP4 5B, and therefore Ph2 and Ph1, comes in the effect on pairing and synapsis, and the presence of multivalents. In duplicating and diverging from ZIP4 3B, ZIP4 5B also gained a pairing function similar to that of Sordaria’ s ZIP4 in terms of promoting homologous pairing. As described above, ZIP4 5B (as well as MSH7 3D) does not prevent homoeologous pairing. ZIP4 5B/Ph1 mutants have over 30 multivalents per 100 meiocytes, 50% pollen viable and 50% seedset. In contrast, the authors are reporting rare multivalents, perhaps four in over 100 meiocytes and in some plants exhibit no multivalents. Martinez et 2001 study cited in the MS actually reported no multivalents in 120Mb Ph2 deletion (ph1a) mutant meiocytes scored at metaphase I. Consistent with this, there is little reduction in pollen viability or seedset with the ph2 mutant , which suggests little disruption of pairing. This makes ph2 mutants very attractive in wheat breeding as the authors comment. I would suggest that this part of discussion needs rewording.

Finally, in the discussion, the authors are implying that ZIP4 would not have predicted from studies of other species to suppress homoeologous crossover in contrast to MSH7, a mismatch repair protein. This is indeed true. In fact, Ph1 would not have been predicted to be ZIP4 from studies on other species. ZIP4 was required for: homologous pairing, synapsis and homologous crossover in Sordaria; synapsis and homologous crossover in budding yeast; and homologous crossover, but not pairing and synapsis in Arabidopsis and rice. So, yes as the authors are indicating, ZIP4 had not previously been shown to suppress homoeologous crossover in any species. Yet ZIP4 is required for processing 85% of homologous crossovers in plants, so it is likely that ZIP4s (on homoeologous group 3 chromosomes) also can normally process homoeologous crossovers as well. The duplicated and diverged ZIP4 5B copy lost this activity. Thus, the loading of both ‘active’ and non-functional’ copies onto the chromosomes could lead to homologous crossovers but suppression of homoeologous crossovers. It will be interesting to see what the activities of MSH7 3A and 3B are in relation to MSH7 3D?. Moreover, the reduction in homologues crossover by MSH7 3D mutant or with the ZIP4 5B mutant contrasts with the recent study by Desjardins et al reporting no reduction in homologous crossover in MSH4 and MSH5 mutants in wheat, only when mutate all copies is homologous crossover reduced and then by 85%. A similar observation was made in polyploid Brassica. This study from the Higgins group suggested ZIP4 activity in wheat might be dosage dependent in contrast to other meiotic crossover genes. A recent talk from the Moore group commented that the group 3 ZIP4s also reduced

homologous crossover. The authors might wish to comment on their thoughts on the suggestion in the Higgins study. given the similarity in crossover effect of MSH7-3D and ZIp4 5B, Is MSH7 also likely to be dosage dependent in terms of its homologous crossover activity and therefore the likely activities of MSH7 3A and 3B?

Reviewer #2 (Remarks to the Author):

The manuscript reports identification of MSH7-3D as the gene controlling chromosome pairing regulation function associated with the Ph2 gene. The presented data is not conclusive to support their claim. While encouraging, the study is not yet ready for publication and additional evidence is needed before any claim for MSH7-3D can be made as the one controlling the Ph2 gene function. I do not support publication of this work because of the following reasons:

1. It is critical to show that a single gene, MSH7-3D is responsible and not other genes, for the Ph2 gene function. Their own deletion mapping data support presence of multiple genes even on 3D for the chromosome pairing function associated with Ph2. The deletion mapping in Figure 1 localizes the gene function between breakpoints of deletions A6S and B 8S as the increase in chromosome pairing is significantly higher in lines with deletions larger than that present in A6S. Yet, in deletion 731, which has much smaller deletion than that present in A6S, increase in chromosome pairing is more than 4.5 fold. The same is the case with A8S and B6S. On the other hand, the deletions with much larger deletions (B7S and 1952) show chromosome pairing significantly less than that in the mutant. These results clearly show involvement of other genes, some increasing and some decreasing chromosome pairing, all present on chromosome 3D.

2. Characterization of Cadenza mutants is a useful approach but the obtained results do not support their claim of MSH7-3D being responsible for the Ph2 function. Of the five lines with mutations in MSH7-3D gene, three show chromosome pairing phenotype similar to the wild-type CS, one shows slight increase in chiasma frequency and another one shows frequency similar to that of the mutant control. Considering that average each mutant plant has mutations in 2000 to 5000 genes thus just one of the five plants with mutations in MSH7-3D gene is troublesome. The phenotype observed in line 2006 could easily be because of mutations in one or more genes with role in chromosome pairing.

3. Ph2 gene function was shown to be present on 3D and not on 3A or 3B. The MSH7 gene has three copies, one each on the three group 3 chromosomes. The three copies show every high level of sequence similarity and the sequence differences in 3D are not responsible for the phenotype (Llyod et al. BMC Plant Biol. 2007; 7: 67). If the sequence difference is not responsible for the Ph2 phenotype then the next possibility is the expression level difference among the three copies. As shown in figure 4 (although this data should not have been presented as there are no error bars for the expression and without error bars, no expression data can be trusted), the 3A copy shows higher expression than the 3B and 3D copies that show very similar expression pattern especially for meiotic stages. So it is clear that neither sequence difference nor expression difference explain the specific function of Ph2 on 3D.

In summary, stable transformation based complementation with the MSH7-3D, multiple independent mutants in the gene showing the phenotype, segregation analysis of the line 2006 on a large population linking the sequence change with the phenotype are some of the acceptable ways to support their claim without which the study should not be published.

Some of the other comments about the manuscript are:
1. The abstract has mostly introduction but very little about what was done and what evidence is being presented to make the case. It needs to be revised accordingly.
2. Introduction: The first paragraph is too general and not really directly related to the study. Perhaps need to be deleted or drastically reduced in size.

3. The authors citation of the literature is troublesome. On many occasions, either they have ignored the original citations (for example, the size of the Ph1 deletion was shown much earlier than 2008 citation. They have also mis-represented the literature about Ph1 gene identification. Reference 13 in the manuscript clearly showed that the claimed 3B duplication on 5B doesn't exist and the statement where the citation 13 is given, is not what was reported in that manuscript. Also, their claims about the importance of Ph2 relative to Ph1 is also misleading. As compared to Ph1, the effect of Ph2 on chromosome pairing regulation is dramatically weaker and they need to give an accurate picture. Such an effort to mislead readers about the literature should never be supported.
4. The claim to use Ph2 for alien introgression is far-fetched and there is no evidence of that in the literature. While it is important to clone this gene to know its function, arguments for its use in alien introgression will have to be clearly documented before this claim can be made.
5. The authors have used chromosome pairing and recombination interchangeably but those are very different processes. They must show recombination before they can claim that recombination has been increased. Reference to recombination should be removed and only chromosome pairing should be mentioned.

Reviewer #3 (Remarks to the Author):

In This manuscript, Serra et al unambigously identified MSH7-3D as the causal gene in the Ph2 mutant. This tour-de-force represents an significant advance in the field of meiotic recombination and in our understandig of the prevention of homeologous recombination in polyploids. The article is well written and the demonstration very clear. I have only a couple of minor suggestions

- L51. ZIP4 is a key protein of the recombination machinery. This role should be presented here.
- L148-190/Figure 3. It would be easier for the reader if the mutants were called by their mutation rather than by the line number (e.g. Tamsh7-3D-D642N rather than Cadenza1114)

Title: "*Pairing homoeologous 2 (Ph2)* encodes the mismatch repair protein MSH7-3D that inhibits homoeologous recombination in wheat"

Manuscript NCOMMS-20-30516

Authors: Serra *et al.*

Dear Nature Communications,

Below we provide a point-by-point response to the reviewer's comments (shown in black), with our text shown in blue.

Sincerely,

Heïdi Serra & Pierre Sourdille

****

Reviewer #1 (Remarks to the Author):

Why Ph2 is important?
On wheat's polyploidisation, Ph1 arose to stabilise tetraploid wheat meiosis through promoting homologous pairing and suppressing homoeologous crossover, so preventing to unbalanced gametes and infertility in this polyploid. The subsequent hybridisation creating hexaploid wheat, brought in an additional locus Ph2 on its D genome, which further helped stabilise the meiosis of this hexaploid by also suppressing homoeologous crossover. Thus, these two loci contribute to stabilising bread wheat as a major global crop, making them of key importance. Moreover, the introgression of traits from wild relatives into hexaploid (bread) wheat exploiting homoeologous recombination has saved the US several billion dollars over the years. Thus, the Ph1 and Ph2 loci in wheat are of key economic importance. Although the introductory information for Gordon and EMBO meiosis meetings state that meiotic research will contribute to breeding, and unpin human health as meiosis can give rise to human aneuploidy. In terms of breeding, exploitation of homoeologous recombination in wheat breeding is still the only actually practical use of meiotic research. Therefore, it was important to identify the genes responsible for Ph1 and Ph2.

The Results-
The homoeologous crossover phenotypes are not trivial to score as they need to be scored through crossing with a wild relative and then scoring meiosis in the hybrids. Thus, unlike most plant traits, Ph2 (or Ph1) can't in practical terms be identified through segregating populations. Instead they have to be initially mapped through deletion analysis, which is a significant undertaking. After an enormous amount work presented in a series of publications, Ph1 has recently been defined to a duplicated copy of the meiotic gene ZIP4 (5B), the papers cited in this MS. The authors now present a significant amount of work as a single publication which clearly defines Ph2 to the meiotic plant gene MSH7-3D. The work required to define this gene involved generating and scoring deletions for the presence, or loss of the Ph2 phenotype, exome capture to show that MSH7 3D copy was indeed mutated in ph2 EMS (ph2b) mutant, expression profiling, exploitation and screening further MSH7 3D EMS mutants and their scoring. The results are clear-cut. The ph2b MSH7 3D ems mutant has a similar phenotype as a 121Mb deletion line ph2a when crossed wild relatives, and the ph2b mutant has a similar level as the Cadenza2006 ems mutant also when crossed to wild relatives. The cadenza2006 mutant has an early stop codon in MSH7 3D, and the authors now show that the ph2b mutant also carries a mutation in its MSH7-3D resulting in a frameshift and early stop codon. Thus, the authors have two MSH7 3D

ems mutants (both producing early stop codons) which give the full homoeologous crossover phenotype. Moreover, when homologous crossover is scored, both mutants have a reduction in this crossover.

I really enjoyed reading this very nice MS.
I have some comments which I hope that the authors find useful.

We thank Reviewer 1 for his interest and recognition of the importance of this study.

The crossover effect-
The results/discussion on the scoring of the other MSH7-3D mutants ought to be de-emphasised. They only yield a partial phenotype, both in terms of both homologous crossover reduction in the mutants themselves, and the level of homoeologous crossover when crossed with wild relatives. It detracts from the clear message for somebody who may not have a detailed knowledge of this area, namely that the authors have MSH7 3D mutants with full Ph2 phenotype when compared to a mutant carrying a 121Mb deletion covering of Ph2 gene. There could be a debate about whether increasing homoeologous crossover by just over one is significant, when this study does not need to go there. The emphasis should be on the candenza2006 and ph2b results. These two MSH7 3D mutants, both have the full phenotype. If the authors are worried that they just have two mutants, they don't need to be as the chance that there is another gene mutated in the 14Mb region giving the same effect in both lines (where one line, ph2b has been grown over a number of generations) would be in the 100's of millions.

We thank the reviewer for this suggestion towards improving message clarity. We agree that presenting several mutants with limited effect on the phenotype does not bring anything essential and could distract from the main message of this paper, i.e. the identification of Ph2. We have accordingly removed data from the independent msh7-3D mutants showing partial phenotypes from the main text and main figures of the manuscript and now placed it in the supporting information. We believe this amendment now contributes to improved ease of interpretation for the reader. We also agree with the reviewers point regarding the low probability of another suppressor gene being located within the 14 Mb region, considering the ph2b mutant has been propagated globally over many generations. In our reply to Reviewer 2, we address this comment directly by documenting and comparing every gene sequence containing an EMS-derived SNP for both ph2b and Cadenza2006 (Tamsh7-3D G605). Please see reply to point 2 of Reviewer 2 below for a detailed explanation of our analysis.*

Thus, in the same vain, it is unclear to me what the cross between 1114 and 2006 tells us or adds, when 1114 only has a partial phenotype? The increase in multivalents in the resulting cross in contrast to rare multivalents (0 or 0.04) in individual mutants themselves probably suggests something else going in the background. It simply complicates. I would suggest removing.

For the purpose of improving clarity for the reader, we agree with the reviewer's comment to remove these data from the manuscript.

I know what the authors are trying to say, but as written the sentence and the word 'pairing' is not correct in 'In contrast to MMR proteins there is no ZIP4 involvement in the inhibition of pairing between homoeologous (divergent) DNA sequences.' When there are no homologues present, related chromosomes (homoeologues) pair and synapse in wheat hybrids after the telomere bouquet with both MSH7D and ZIP4 5B present (Nat communs 2014, Chromosoma 2017 papers both cited), and the recombination machinery appears to load. Two barley homoeologues still paired in wheat itself when ZIP4 5B or MSH7 3D is present (Prieto group Frontiers 2018 study). Thus, neither MSH7 3D nor ZIP4

5B can prevent homoeologues pairing and synapsing, instead, they both prevent successful crossovers from occurring between such paired diverged chromosomes. This ought to reworded.

We agree with Reviewer 1's comment regarding the terminology used and have amended the text accordingly. Lines 347-349 have now been amended from "In contrast to MMR proteins there is no ZIP4 involvement in the inhibition of pairing between homoeologous (divergent) DNA sequences." to "In contrast to MMR proteins, there is no indication of ZIP4 involvement in promoting heteroduplex rejection following DNA-strand exchange between divergent sequences."

The mutation of MSH7 3D (or ZIP4 5B) also both lead to a similar reduction in homologous crossover. Thus in terms of crossover, both the mutation of MSH7 3D or ZIP4 5B lead to a similar reduction in homologous crossover, and an increase in homoeologous crossover. The only slight difference in crossover is that mutation of MSH7 3D induces two fold less homoeologous crossovers than ZIP4 5B. Thus, Ph2 and Ph1 in terms of crossover are essentially very similar. ZIP4 is a tetratricopepticide repeat protein, which basically can form folds for binding proteins and assembling protein complexes, akin to a docking station. Given the similarity in their crossover effect, it raises the question as to whether MSH7-3D is part of one of these complexes?

We thank the Reviewer 1 for raising this excellent point regarding a potential interaction between MSH7-3D / Ph2 and ZIP4-B2 / Ph1. It is indeed plausible that both proteins are part of the same complex (considering same induced phenotype, irrespective of strength). However, one must take into account likely differences in the timing of events. MMR complexes are known to recognize mismatches formed during strand invasion and induce subsequent dissociation of the invading strand (heteroduplex rejection), as this would be indicative of MSH7-3D acting in the first steps of the recombination process (Chakraborty et al, 2016). Comparatively, recent data highlighted a role for ZIP4-B2 / Ph1 later in meiosis – once Double Holiday junctions have been established between divergent sequences – in inhibiting these recombination intermediates to become crossovers (Martin et al. 2014, 2017). Albeit speculative, one could envisage that different modes of action and timing of Ph1 relative to Ph2, could result in stronger homoeologous recombination suppression. Additional experiments to immunohistochemically detect MSH7-3D and ZIP4-B2 over the course of meiotic prophase I, would go some way to resolving possible temporal differences and co-locality in the same complex.

Pairing

The difference between MSH7-3D and ZIP4 5B, and therefore Ph2 and Ph1, comes in the effect on pairing and synapsis, and the presence of multivalents. In duplicating and diverging from ZIP4 3B, ZIP4 5B also gained a pairing function similar to that of Sordaria' s ZIP4 in terms of promoting homologous pairing. As described above, ZIP4 5B (as well as MSH7 3D) does not prevent homoeologous pairing. ZIP4 5B/Ph1 mutants have over 30 multivalents per 100 meiocytes, 50% pollen viable and 50% seedset. In contrast, the authors are reporting rare multivalents, perhaps four in over 100 meiocytes and in some plants exhibit no multivalents. Martinez et 2001 study cited in the MS actually reported no multivalents in 120Mb Ph2 deletion (ph1a) mutant meiocytes scored at metaphase I. Consistent with this, there is little reduction in pollen viability or seedset with the ph2 mutant , which suggests little disruption of pairing. This makes ph2 mutants very attractive in wheat breeding as the authors comment. I would suggest that this part of discussion needs rewording.

We agree with Reviewer 1 that an interesting difference observed between msh7-3D and *zip4-B2 meiocytes is the frequency of multivalent associations (involving homoeologous chromosomes) at metaphase I (0 to 0.04 vs 0.3 multivalent / meiocyte, respectively). This is indeed consistent with the difference observed in msh7-3D vs zip4-B2 fertility (0 vs 50% of reduction in seed set compared to wild-type, respectively). This could be indicative of a role for ZIP4-B2, but not MSH7-3D, in promoting homologous pairing.*

We are somewhat unsure exactly what Reviewer 1 would like re-worded, but we infer that we should draw attention to the fact that multivalent associations observed in msh7-3D are infrequent (as opposed to the substantial increase in multivalent frequency observed in zip4-B2 compared to wild-type). This clarification has been provided at Line 326: "In some Tamsh7-3D / ph2 wheat meiocytes, such a reduction in homologous recombination is associated with the presence of rare multivalents resulting from homoeologous recombination"

Finally, in the discussion, the authors are implying that ZIP4 would not have predicted from studies of other species to suppress homoeologous crossover in contrast to MSH7, a mismatch repair protein. This is indeed true. In fact, Ph1 would not have been predicted to be ZIP4 from studies on other species. ZIP4 was required for: homologous pairing, synapsis and homologous crossover in Sordaria; synapsis and homologous crossover in budding yeast; and homologous crossover, but not pairing and synapsis in Arabidopsis and rice. So, yes as the authors are indicating, ZIP4 had not previously been shown to suppress homoeologous crossover in any species. Yet ZIP4 is required for processing 85% of homologous crossovers in plants, so it is likely that ZIP4s (on homoeologous group 3 chromosomes) also can normally process homoeologous crossovers as well. The duplicated and diverged ZIP4 5B copy lost this activity. Thus, the loading of both 'active' and non-functional' copies onto the chromosomes could lead to homologous crossovers but suppression of homoeologous crossovers. It will be interesting to see what the activities of MSH7 3A and 3B are in relation to MSH7 3D?

We fully agree with Reviewer 1 that characterizing the activities of MSH7-3A and 3B in relation with MSH7-3D is of great interest. Ongoing experiments are aimed at investigating potential roles for MSH7-3A and 3B in both homologous and homoeologous recombination, and the functional interactions between all three homoeologous proteins.

Moreover, the reduction in homologues crossover by MSH7 3D mutant or with the ZIP4 5B mutant contrasts with the recent study by Desjardins et al reporting no reduction in homologous crossover in MSH4 and MSH5 mutants in wheat, only when mutate all copies is homologous crossover reduced and then by 85%. A similar observation was made in polyploid Brassica. This study from the Higgins group suggested ZIP4 activity in wheat might be dosage dependent in contrast to other meiotic crossover genes. A recent talk from the Moore group commented that the group 3 ZIP4s also reduced homologous crossover. The authors might wish to comment on their thoughts on the suggestion in the Higgins study. given the similarity in crossover effect of MSH7-3D and ZIp4 5B, Is MSH7 also likely to be dosage dependent in terms of its homologous crossover activity and therefore the likely activities of MSH7 3A and 3B?

Reviewer 1 raises an important point regarding the effect of MSH gene dosage on homologous recombination. Both the Higgins and Jenczewski groups have shown that only one gene copy for MSH4 and MSH5 is enough to maintain homologous crossovers in wheat and Brassica, whereas single gene mutations of ZIP4-5B (Rey et al., 2017) and MSH7-3D each induce a slight but significant reduction (-8%) in homologous crossovers, indicative of ZIP4 and MSH7 being dosage dependent in wheat. We tested the hypothesis of MSH7-3D dosage dependence by quantifying chiasma frequency when only one copy of MSH7-3D is present (ie. msh7-3D heterozygote) relative to two. We observed an intermediate phenotype between wild-type and msh7-3D homozygotes (table 1 and figure 1 below), consistent with MSH7 being dosage dependent. These data have been added to the manuscript (Lines 191-195; Supplementary Table S5 and Supplementary Figure S5). In the context of its homoeologues (MSH7-3A, MSH7-3B), which do not show major differences in expression nor protein coding, one could expect a degree of functional redundancy, a research line we are actively investigating. We are particularly interested in the effect of a triple knock-out on homologous crossover frequency.

Table 1. *Meiotic phenotype at metaphase I in wild-type wheat cv. Cadenza (TaMSH7-3D +/+) and homozygote (TaMSH7-3D -/-) and heterozygote (TaMSH7-3D +/-) Cadenza2006 mutants. n = number of cells examined. Mean ± SEM (range).*

Lines	n	Univalents	Rod bivalents	Ring bivalents	Multivalents	Chiasma frequency
TaMSH7-3D -/-	109	1.71 ± 0.16 (0-8)	2.69 ± 0.15 (0-9)	17.39 ± 0.15 (11-20)	0.04 ± 0.02 (0-1)	**37.58 ± 0.19** (31-41)
TaMSH7-3D +/+	110	0.18 ± 0.06 (0-2)	0.88 ± 0.08 (0-4)	20.03 ± 0.08 (17-21)	0.00 ± 0	**40.94 ± 0.09** (38-42)
TaMSH7-3D +/-	46	1.65 ± 0.11 (0-2)	1.26 ± 0.16 (0-4)	18.91 ± 0.17 (16-21)	0.00 ± 0	**39.09 ± 0.19** (36-42)

Figure 1. *Meiotic phenotype at metaphase I in TaMSH7-3D -/-, TaMSH7-3D +/+ and TaMSH7-3D +/-. Box plots showing minimum, first quantile, median (horizontal middle line), third quantile and maximum count of chiasma frequency per meiocyte. Mean values are represented by a red cross.*

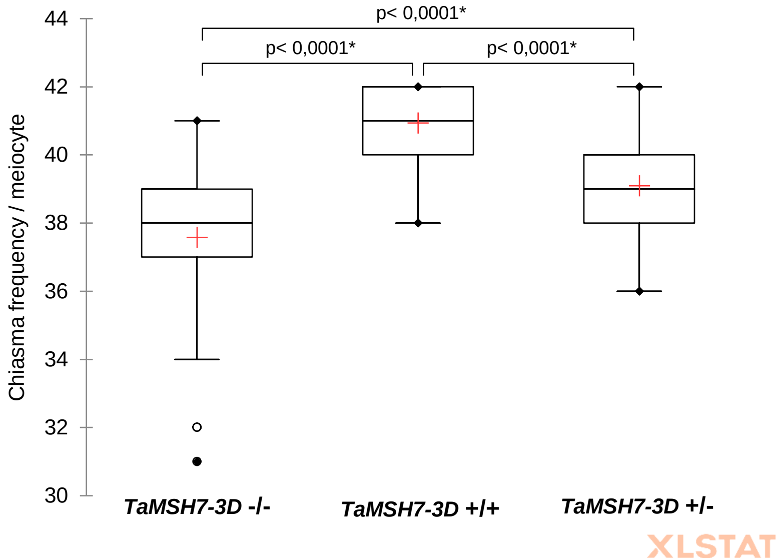

* : *significance level* α = 0.05 (Mann Whitney tests with Bonferroni adjustment for multiple testing)

Reviewer #2 (Remarks to the Author):

The manuscript reports identification of MSH7-3D as the gene controlling chromosome pairing regulation function associated with the Ph2 gene. The presented data is not conclusive to support their claim. While encouraging, the study is not yet ready for publication and additional evidence is needed before any claim for MSH7-3D can be made as the one controlling the Ph2 gene function. I do not support publication of this work because of the following reasons:

1. It is critical to show that a single gene, MSH7-3D is responsible and not other genes, for the Ph2 gene function. Their own deletion mapping data support presence of multiple genes even on 3D for the chromosome pairing function associated with Ph2. The deletion mapping in Figure 1 localizes the gene function between breakpoints of deletions A6S and B 8S as the increase in chromosome pairing is significantly higher in lines with deletions larger than that present in A6S. Yet, in deletion 731, which has much smaller deletion than that present in A6S, increase in chromosome pairing is more than 4.5 fold. The same is the case with A8S and B6S. On the other hand, the deletions with much larger deletions (B7S and 1952) show chromosome pairing significantly less than that in the mutant. These results clearly show involvement of other genes, some increasing and some decreasing chromosome pairing, all present on chromosome 3D.

We agree with Reviewer 2 that demonstrating MSH7-3D is the unique causative gene for ph2 is essential. At the beginning of our project, only two reference mutants existed: ph2a, carrying a 80 Mb deletion (we showed that deletion size is actually 121 Mb) and ph2b, an EMS mutant. We first applied a commonly used deletion mapping approach to reduce the size of the deletion containing Ph2 (as it was done for Ph1 (Griffith et al., 2006)). Reviewer 2 is drawing attention to the variability inherent of assessing chiasma frequency in interspecific hybrids using terminal deletions of varying sizes; this is a challenging task in itself based on the number of cytological examinations required for each hybrid combination. Despite the interpretation of finer differences between lines 731 versus CS and B7S/1952 versus ph2a, it does not negate the strong effect of B8S and larger terminal deletions that are all within the same range as ph2a. This therefore demonstrates that the major effect on increase of homoeologous chiasma frequency is located between breakpoints of deletion A6S and B8S, placing the major effect of "Ph2" directly within this region. Exome capture analysis of ph2b identified only one meiotically expressed gene with a loss-of-function EMS derived SNP (TaMSH7-3D) in this interval.

We recognise it is somewhat debatable whether the difference between lines 1913 and 731 (for example) represents the position of an additional suppressor of homoeologous recombination. As the subsequent deletion lines (303, A185) exhibit reduced chiasma frequency compared to 731, it would also imply the existence of a promotor of homoeologous recombination between breakpoints of deletion 731 and 303. It is therefore necessary to assess this data and the speculation of additional promoters/suppressors raised by Reviewer 2 in light of the genome-wide exome capture analysis of ph2b. As an example, the region of the speculative suppressor between 1913 and 731 breakpoints only contains an LRR-family member with a putative loss-of-function (W → stop) that is not expressed in meiotic tissues of CS nor ph2b; and a helicase with an intronic SNP with no consequence on protein coding (see Annex 1, DeletionLines). Considering ph2b and the deletion mutant series are all in Chinese Spring backgrounds, the conclusion is that there is no evidence for an additional gene contributing to the ph2 phenotype. An important aspect also relates to the "2C gametocidal system" (Endo and Gill, 1996; Svacina et al, 2020) that was used to generate the terminal deletion series. Deletion size and chromatin state changes between lines, as well as potential off-target deletions, could inadvertently contribute to some slight variability in genome-wide chiasma frequency, and therefore be responsible for the observed variation.

We agree that the deletion line mapping data alone do not formally provide proof that MSH7-3D is the only gene contributing to Ph2 function. Our conclusion is however drawn from the in-depth analysis of three independent mutants (ph2a, ph2b & Cadenza2006) exhibiting the full ph2 homoeologous crossover phenotype (see response to comment 2 below).

2. Characterization of Cadenza mutants is a useful approach but the obtained results do not support their claim of MSH7-3D being responsible for the Ph2 function. Of the five lines with mutations in MSH7-3D gene, three show chromosome pairing phenotype similar to the wild-type CS, one shows slight increase in chiasma frequency and another one shows frequency similar to that of the mutant control. Considering that average each mutant plant has mutations in 2000 to 5000 genes thus just one of the five plants with mutations in MSH7-3D gene is troublesome. The phenotype observed in line 2006 could easily be because of mutations in one or more genes with role in chromosome pairing.

Reviewer 2 indicates that other genes affecting chiasma frequency could also be mutated in Cadenza2006 and is therefore not enough evidence towards our claim that MSH7-3D represents Ph2. This point is divergent with Reviewer 1's ("the results are clear cut" and "the authors have two MSH7-3D EMS mutants (both producing early stop codons) which give the full homoeologous crossover phenotype") and Reviewer 3's ("Serra et al unambiguously identified MSH7-3D as the causal gene in the Ph2 mutant") statements. In response to Reviewer 2's claim that there could be other gene(s) mutated in Cadenza2006 that would contribute to the phenotype, we have now documented all genes containing EMS-derived SNPs in this particular line and directly compared these with all those we had previously identified in our genome wide exome capture of ph2b. Cadenza2006 contains 3342 SNPs (both het and homozygote) in the genome-wide gene space (5'UTR, 3'UTR, exons, introns, splice regions and promoters) for which 963 are missense, 494 are synonymous, 90 are splice variants and 40 result in a likely loss-of-function due to loss of the start codon or contain premature stops. Comparatively, ph2b contains 2370 unique homozygous SNPs in the genome-wide gene space for which 1223 are missense, 713 are synonymous, and 43 either effect the start codon or contain premature stops. Only 16 mutated genes show commonality between these two lines (Annex 1, CommonMutations). Ph2 gene function was previously shown to be present on 3DS based on ph2a mutant analysis, and we confirm this data using 10 independent 3DS deletion lines. Among the 16 genes mutated in both ph2b and Cadenza2006 lines, only MSH7-3D is located on 3DS and none of the 15 other genes carry mutation in ph2a. One should also point out that none of the weaker Cadenza mutant alleles we present in this study contain mutations in either of these 16 gene sequences, except for MSH7-3D. Collectively, these data demonstrate that the only possible gene that could be responsible for the ph2 phenotype in these three independent mutants (ph2a, ph2b & Cadenza2006) is MSH7-3D.

These additional data have been added to the manuscript (Lines 209-213; Supplementary Table S6).

3. Ph2 gene function was shown to be present on 3D and not on 3A or 3B. The MSH7 gene has three copies, one each on the three group 3 chromosomes. The three copies show every high level of sequence similarity and the sequence differences in 3D are not responsible for the phenotype (Llyod et al. BMC Plant Biol. 2007; 7: 67). If the sequence difference is not responsible for the Ph2 phenotype then the next possibility is the expression level difference among the three copies. As shown in figure 4 (although this data should not have been presented as there are no error bars for the expression and without error bars, no expression data can be trusted), the 3A copy shows higher expression than the 3B and 3D copies that show very similar expression pattern especially for meiotic stages. So it is clear that neither sequence difference nor expression difference explain the specific function of Ph2 on 3D.

We agree with points Reviewer 2 raises, however we would like to draw Reviewer 2's attention to several discrepancies identified in the Lloyd paper during the course of our investigations. This has implication on Reviewer 2's interpretation of the data. Details are outlined in point 1, below. We recognize the deficiency in the presented expression data and would like to draw on an independent and extensive dataset that presents the same conclusion on expression analysis we presented in Figure 4, outlined in point 2, below.

1. *The sequencing of TaMSH7-3D by Lloyd et al. (2007), and submitted to NCBI as AF354709.1 contains several errors as a consequence of homoeologue amplicon chimeras, an artifact of non-*

specific PCR based amplification. Alignment of this sequence against genomic sequences of the three TaMSH7 homoeologues clearly shows the chimeric nature of Lloyd's sequence (see Annex 2). Consequently, they did not identify the causative SNP we demonstrate in this study.

2. *Alabdullah et al. (2009) analysed 130 RNAseq datasets for meiotic gene co-expression. This analysis revealed that TaMSH7 is present as an expression balanced triad exhibiting no expression dominance. This finding is the same as can be concluded from Figure 4.*

According to point 2, we amend Line 242 to incorporate reference to Alubdullah et al., 2019.

In summary, stable transformation based complementation with the MSH7-3D, multiple independent mutants in the gene showing the phenotype, segregation analysis of the line 2006 on a large population linking the sequence change with the phenotype are some of the acceptable ways to support their claim without which the study should not be published.

We acknowledge the proposal by reviewer 2 that many different approaches are typically used to demonstrate gene functionality, however we believe reviewer 2 is disregarding the added complexities of such analysis for this particularly challenging phenotype, a point confirmed by Review 1. We believe the evidence we provide, through the 'collective' analysis of multiple mutant alleles and as argued in the points above, is substantive enough to draw the conclusion of TaMSH7 being the causative gene for Ph2. Our view of the collective evidence provided is fully endorsed by both reviewers 1 & 3.

Some of the other comments about the manuscript are:
1. The abstract has mostly introduction but very little about what was done and what evidence is being presented to make the case. It needs to be revised accordingly.

2. Introduction: The first paragraph is too general and not really directly related to the study. Perhaps need to be deleted or drastically reduced in size.

We have followed the Nature Communications editorial guidelines and additionally followed style of many published Nat Comm papers to ensure that the Abstract and Introduction sections are within scope of the journal, specifically to allow broad readership. As such we believe we articulated the content accordingly. We are however open to modification at the editor's request.

3. The authors citation of the literature is troublesome. On many occasions, either they have ignored the original citations (for example, the size of the Ph1 deletion was shown much earlier than 2008 citation. They have also mis-represented the literature about Ph1 gene identification. Reference 13 in the manuscript clearly showed that the claimed 3B duplication on 5B doesn't exist and the statement where the citation 13 is given, is not what was reported in that manuscript. Also, their claims about the importance of Ph2 relative to Ph1 is also misleading. As compared to Ph1, the effect of Ph2 on chromosome pairing regulation is dramatically weaker and they need to give an accurate picture. Such an effort to mislead readers about the literature should never be supported.

We acknowledge Reviewer 2's intention to draw our attention to the citations used in the manuscript. Rather than assembling a citation list that accommodates the research of all authors who have published in the field, we have instead and appropriately focused citations to those works that provide the most robust and conclusive evidence related to the points being made. For example, in Line 50 we state "Ph1 mutant analysis (ph1b) has identified a 2.5 Mbp region deleted on chromosome 5BL" referencing Griffith's et al., 2006 Nature paper, whom determined the physical size based on chromosome walking utilizing a tiled array of isolated wheat BAC clones. Whereas much earlier studies, as pointed out by Reviewer 2, were only estimates of the deletion size, based on a combination of optical analysis by

chromatin ISH/C-banding and genetic mapping (Gill et al., 1991; Gill et al., 1993). We believe comparatively the more accurate methodology to determine the physical size in Mbp is chromosome walking using BAC clones.

In our referencing attempt to appropriately acknowledge several competing groups Ph1 mapping efforts and candidate gene testing over many years, we inadvertently included Bhullar et al., 2014, which should have been Sidhu et al., 2008 of the same group. We additionally recognize this paper identifies an issue of contention in the literature about the presence of the "duplicated 3B-chromosome segment containing ZIP4" therefore we have amended this sentence to "This region contains a meiotic gene ZIP4 and a heterochromatin tandem repeat block, inserted within a cluster of CDK2-like genes" (Lines 50-52). The follow-up study by Rey et al., 2017 isolated the ZIP4 sequence from chromosome 5B, a point disputed by Sidhu et al., 2008.

Reviewer 2's insinuation that we are intentionally misleading readers about the literature is unwarranted, as we accurately state in Line 351 that ph2 phenotype is half as strong as ph1. This is evidenced from Mello-Sampayo & Canas, 1973 then reported by Sear's in his 1976 review. Furthermore, it is self-evident that the high phenotype of ph1 has resulted in its broader use for introgression breeding.

4. The claim to use Ph2 for alien introgression is far-fetched and there is no evidence of that in the literature. While it is important to clone this gene to know its function, arguments for its use in alien introgression will have to be clearly documented before this claim can be made.

We would like to point Reviewer 2 to the following research articles that provide evidence for ph2b's demonstrated use in introgression breeding, keeping in mind introgression is defined as the transfer of genetic information from one species to another as a result of hybridisation between them and repeated backcrossing.

Wei et al., 2015, utilised ph2a and ph2b mutants for introgressing Aegilops sharonensis chromatin containing HMW-GSs, whilst Benavente et al., 1998 confirmed a wheat/rye translocation event by GISH using ph2b. However, more importantly, Marais et al., 1992 utilised ph2b to break linkage drag between yellow seed pigment and leaf rust resistance on an existing introgression fragment from Thinopyrum distichum. Therefore, collectively, this demonstrates ph2 has been used for introgression breeding.

We also recognise that proportionately there are many more examples for ph1's use, but this is a consequence of its stronger effect on homoeologous recombination. This does not however, negate the inherent value of ph2, particularly regarding its potential for combinatorial use with ph1 and/or Hpp-5Mg ph1 inducer, as Reviewer 1 has articulated clearly.

5. The authors have used chromosome pairing and recombination interchangeably but those are very different processes. They must show recombination before they can claim that recombination has been increased. Reference to recombination should be removed and only chromosome pairing should be mentioned.

In recent reviews from Naranjo & Benavente (2015), Zickler & Kleckner (2015) and Da Ines & White (2015), the term 'chromosome pairing' stricto sensu refers to the alignment of chromosomes prior to synapsis during meiotic prophase I, although it has been historically employed to describe chromosome behaviour at metaphase I [1]. The chromosome associations (bivalents, multivalents) observed at metaphase I correspond to chromosomes linked by (one or more) chiasma(ta), the cytological visualisation of meiotic crossovers. The term 'recombination' should thus be employed when referring to chromosome configuration at metaphase I (in agreement with all recent publications in the field).

We paid special attention to accurately refer to 'recombination' instead of the old term 'chromosome pairing' when preparing the manuscript, except when we were directly referring to old literature data. We have changed the text to be fully consistent with the 'new' terminology.

[1] *« In the strict sense, the term chromosome pairing refers to the alignment of homologous chromosomes prior to synapsis. However, the terms homologous pairing and homoeologous pairing, which appear repetitively along the text (and in the literature), have been employed in the wide sense that they had at the time of the discovery of the pairing regulating genes, i.e., the association of homologous or homoeologous chromosomes at metaphase I. » (Naranjo & Benavente, 2015)*

Reviewer #3 (Remarks to the Author):

In This manuscript, Serra et al unambigously identified MSH7-3D as the causal gene in the Ph2 mutant. This tour-de-force represents an significant advance in the field of meiotic recombination and in our understandig of the prevention of homeologous recombination in polyploids. The article is well written and the demonstration very clear. I have only a couple of minor suggestions

We are pleased the Reviewer appreciates the significance of our work.

- L51. ZIP4 is a key protein of the recombination machinery. This role should be presented here.

We have added a description of ZIP4 in the introduction (Lines 52-54): "ZIP4 is a ZMM protein involved in homologous recombination and may act as a hub through physical interactions with components of the chromosome axis and other ZMMs (De Muyt et al. 2018)."

- L148-190/Figure 3. It would be easier for the reader if the mutants were called by their mutation rather than by the line number (e.g. Tamsh7-3D-D642N rather than Cadenza1114)

We thank Reviewer 3 for this suggestion. We have changed the line numbers by corresponding mutations in the text and figures.

Reviewer #1 (Remarks to the Author):

The authors have addressed all the issues which I raised. I also believe that they have addressed referees 2 comments in their response. The simplification of the results focusing on the key mutants showing the full phenotype makes the story easier to follow and improves clarity. I believe that the MS should be published.

With respect to Referee 2's comment in their review 'clearly showed that the claimed 3B duplication on 5B doesn't exist'.

Actually, this duplication was confirmed by the international wheat genome sequencing consortium with the publication of their high quality wheat genome sequence. Their Science 2018 paper states ' Two of the genes in this category were granule bound starch synthase (GBSS) on chromosome 4A (1:0:0, a gene that is a key determinant of udon noodle quality) and ZIP4 within the pairing homeologous 1 (Ph1) locus on chromosome 5B (0:1:0, a locus critical for the diploid meiotic behavior of the wheat homeologous chromosomes (25). The phylogenomic analysis indicated that the GBSS on 4A is a divergent translocated homeolog originally located on chromosome 7B (fig. S16), whereas ZIP4 is a transduplication of a chromosome 3B locus (table S26).'

Thus, Ensembl Plants wheat has 4 annotated ZIP4 genes for hexaploid wheat, a copy on 3A, 3B, 3D, and 5B (ZIP4 5B being TraesCS5B02G255100), and 3 annotated ZIP4 genes for tetraploid wheat on 3A, 3B and 5B (ZIP4 5B being TRITD5Bv1G235560 and TRIDC5BG042320 for turgidum and dicoccoides respectively).

CRISPR deletion of ZIP4 5B reveals that it controls pairing, homologous and homoeologous crossover in wheat and wheat-wild relative hybrids (Rey et al., 2018).

Reviewer #2 (Remarks to the Author):

Many of my original comments are still unaddressed so my original review stands.

Reviewer #3 (Remarks to the Author):

The revission of the manuscript added further clarity to the story. I believe that the authors have satisfactory adressed all the points raised by the three reviewers.

Title: “*Pairing homoeologous 2 (Ph2)* encodes the mismatch repair protein MSH7-3D that inhibits wheat homoeologous recombination”

Manuscript NCOMMS-20-30516

Authors: Serra *et al.*

Dear Nature Communications,

Please find below a response to the reviewer’s comments (shown in black), with our text shown in blue.

Yours sincerely,

Heïdi Serra & Pierre Sourdille

****

Reviewer #1 (Remarks to the Author):

The authors have addressed all the issues which I raised. I also believe that they have addressed referees 2 comments in their response. The simplification of the results focusing on the key mutants showing the full phenotype makes the story easier to follow and improves clarity. I believe that the MS should be published.

With respect to Referee 2’s comment in their review ‘clearly showed that the claimed 3B duplication on 5B doesn’t exist’.

Actually, this duplication was confirmed by the international wheat genome sequencing consortium with the publication of their high quality wheat genome sequence. Their Science 2018 paper states ‘ Two of the genes in this category were granule bound starch synthase (GBSS) on chromosome 4A (1:0:0, a gene that is a key determinant of udon noodle quality) and ZIP4 within the pairing homeologous 1 (Ph1) locus on chromosome 5B (0:1:0, a locus critical for the diploid meiotic behavior of the wheat homeologous chromosomes (25). The phylogenomic analysis indicated that the GBSS on 4A is a divergent translocated homeolog originally located on chromosome 7B (fig. S16), whereas ZIP4 is a transduplication of a chromosome 3B locus (table S26).’

Thus, Ensembl Plants wheat has 4 annotated ZIP4 genes for hexaploid wheat, a copy on 3A, 3B, 3D, and 5B (ZIP4 5B being TraesCS5B02G255100), and 3 annotated ZIP4 genes for tetraploid wheat on 3A, 3B and 5B (ZIP4 5B being TRITD5Bv1G235560 and TRIDC5BG042320 for turgidum and dicoccoides respectively).

CRISPR deletion of ZIP4 5B reveals that it controls pairing, homologous and homoeologous crossover in wheat and wheat-wild relative hybrids (Rey et al., 2018).

Reviewer #2 (Remarks to the Author):

Many of my original comments are still unaddressed so my original review stands.

Reviewer #3 (Remarks to the Author):

The revission of the manuscript added further clarity to the story. I believe that the authors have satisfactory adressed all the points raised by the three reviewers.

We greatly thank the Reviewers for their positive inputs on our manuscript.
